# Supplementary material for: Prehospital stratification in acute chest pain patient into high risk and low risk by emergency medical service: a prospective cohort study
Source: BMJ Open. 2021 Apr 15;11(4):e044938. doi: 10.1136/bmjopen-2020-044938 (PMC8055143; doi:10.1136/bmjopen-2020-044938)
Supplement: Supplementary data [file bmjopen-2020-044938supp001.pdf]

Supplemental material 1 - Analog version of study questionnaire

1. Patient is (several options possible)?
□Pale □Clammy □Neither □ Can't tell

2. Patient is nauseas, is vomiting or has been vomiting (several options possible)?
□Nausea □Vomiting/has been vomiting □Neither □ Can't tell

3. The patient experiences his/her breathing as affected?
□Affected □Unaffected □ Can't tell

4. Pain intensity (when resting) on EMS arrival according to NRS (0-10, 0 = "no pain", 10 = "worst imaginable pain").
NRS: 0 1 2 3 4 5 6 7 8 9 10

If NRS is not possible to use, ask the patient to rate his/her pain as follows:
□No pain □Mild pain □Moderate pain □Severe pain □Unbearable pain

5. At what time and date did the pain, inducing the EMS contact, start?
Time: □ Can't tell
Date:

6. How did the pain start?
□Suddenly (seconds) □Quickly (minutes) □Slowly (hours) □ Can't tell

7. Did the pain start in conjunction with (several options possible)?
□Activity □While resting □In pain when waking up □ Can't tell

8. Development of pain (several options possible).
□Pain is constant □Pain come and go □Pain increases gradually □ Can't tell

9. How does the patient describe his/her pain (several options possible)?
Do the patient describe his/her pain as pressuring, cramping, burning, cutting, stabbing, dull, discomfort, swaying, bursting etc. Please write below.

10. Chest pain localisation (several options possible)?
□Central □Left side of chest □Right side of chest □Upper half of chest
□Lower half of chest □Entire chest □ Can't tell

11. Do the patient have pain somewhere else other than in their chest (several different areas/body parts can be specified).
Please write below which area/body part that hurts or if the chest pain is radiating somewhere. If no other pain than chest pain, write: "No other pain".

12. Size of area affected by pain?
□Size of a 5 sek coin □Size of a palm □Entire chest □ Can't tell

13. Chest pain affected by palpation?
□Yes □No □ Can't tell

14. Is pain affected by movement?
□Yes □No □ Can't tell

15. Is pain affected by breathing?
□Yes □No □ Can't tell
