# Supplementary material for: Prehospital stratification in acute chest pain patient into high risk and low risk by emergency medical service: a prospective cohort study
Source: BMJ Open. 2021 Apr 15;11(4):e044938. doi: 10.1136/bmjopen-2020-044938 (PMC8055143; doi:10.1136/bmjopen-2020-044938)
Supplement: Supplementary data [file bmjopen-2020-044938supp002.pdf]

Supplemental material 2 - ECG interpretation manual

1. Heart rate: Provided by computer.
2. Sinus rhythm: Positive P wave in lead II with a constant morphology, rate between 50 and 100/min, constant PQ interval and normal QRS morphology.(2)
3. Sinus Bradycardia: Positive P-wave in lead II with a constant morphology, rate <50/min, constant PQ interval and normal QRS morphology.(2)
4. Sinus Tachycardia: Positive P wave in lead II with a constant morphology, rate >100/min, constant PQ interval and normal QRS morphology.(2)
5. Supraventricular Tachycardia: Fast and regular ventricular rhythm usually between 150-200/min. Episodic presentation with sudden onset. Normal QRS-complexes.(2)
6. Atrial Fibrillation: No evident P-waves instead the ECG reveals low-amplitude baseline oscillations (fibrillary or f waves from the fibrillating atria) and an irregular ventricular rhythm. Normal QRS complex duration during AV conduction.(2, 5)
7. Atrial Flutter: In typical atrial flutter, the ECG reveals identically recurring, regular, sawtooth flutter waves, and evidence of continual electrical activity (lack of an isoelectric interval between flutter waves), often best visualized in leads II, III, aVF, or V1. The atrial rate during typical atrial flutter is usually 250 to 350 beats/min with variations in the degree of ventricular conduction, usually 2:1 conduction. Normal QRS complex duration during AV conduction.(2, 5)
8. Atrial pacing: Pacing spike precedes the p wave. Morphology of p wave dependent of lead placement but may appear normal.
9. Ventricular pacing: Pacing spike precedes the QRS complex.
10. Second Degree AV block – II type 1: Progressive prolongation of the PR interval culminating in a non-conducted P wave. The PR interval is longest immediately before the dropped beat. The PR interval is shortest immediately after the dropped beat.(5)
11. Second Degree AV block – II type 2: Intermittent non-conducted P waves without progressive prolongation of the PR interval. The PR interval in the conducted beats remains constant. The P waves continue at a constant rate. The RR interval surrounding the dropped beat(s) is an exact multiple of the preceding RR interval.(5)
12. Third Degree AV block – III: Normal P waves with regular rhythm. Complete absence of AV conduction – none of the supraventricular impulses are conducted to the ventricles. Perfusing rhythm is maintained by a junctional or ventricular escape rhythm.(5)
13. Suspected STEMI: ST-elevation of the J-point in two contiguous leads with ≥ 2.5 mm in men < 40 years, ≥ 2 mm in men > 40 years, or ≥ 1,5 mm in women in V2-V3 and/or ≥ 1 mm in the other leads.(1)
14. ST-Depression: ST-depression ≥ 0.5 mm in at least two contiguous leads. (3)
15. T-wave inversion: Negative electrical axis of the T wave. Positive T wave is always expected at least in the precordial leads V2-V6 and in AVL where the R wave is at least 5mm, deviation from this rule is pathologic.
16. Q-wave: Pathologic q-wave ≥ 25% of R-wave or duration of ≥0,03 s in two contiguous leads. Q-wave or QS-komplex in V2 or V3 is always pathologic. (2)
17. QRS-duration: Provided by computer.
18. Premature Ventricular Complex-PVC: Characterized by the premature occurrence of a QRS complex that is abnormal in shape and has a duration usually exceeding the dominant QRS complex, generally longer than 120 milliseconds. The T wave is usually large and opposite in direction to the major deflection of the QRS. The QRS complex is not preceded by a premature P wave but can be preceded by a nonconducted sinus P wave occurring at its expected time.(5)
19. PAC-SVES: Premature Atrial Complexes: Premature beat with or without a premature P wave that generally differs from a normal sinus P wave. Normal QRS complex. (2,5)

1. Left Bundle Branch Block – LBBB: QRS duration of ≥ 120 ms. Dominant S wave in V1-V3. Broad M-shaped QRS complexes in lateral leads (V5-V6), ST and T waves usually opposite in direction to QRS. Absence of Q waves in lateral leads (I, V5-V6; small Q waves are still allowed in aVL). Prolonged R wave peak time > 60ms in left precordial leads (V5-6).(5,6)
2. Right Bundle Branch Block – RBBB: QRS duration ≥ 120 msec. rsr′, rsR′, or rSR′, patterns in leads V1 and V2. S waves in leads I and V6 ≥ 40 msec wide. Normal time to peak R wave in leads V5 and V6 but > 50 msec in V1.(5)
3. Ventricular Tachycardia: VT is suggested by the occurrence of a series of three or more consecutive, abnormally shaped QRS complexes longer than 120 milliseconds, with the ST-T vector pointing opposite the major QRS deflection. The R-R interval can be regular or varying. Atrial activity can be independent of ventricular activity (AV dissociation), or the atria can be depolarized retrogradely (ventriculoatrial [VA] association). Depending on the particular type of VT, rates range from 70 to 250 beats/min, usually > 100 beats/min.(2,5)
4. Uninterpretable ECG: Meaningful ECG interpretation not possible due to low ECG quality as judged by interpreter.

References

1. Ibanez B, James S, Agewall S, Antunes MJ, Bucciarelli-Ducci C, Bueno H, et al. 2017 ESC Guidelines for the management of acute myocardial infarction in patients presenting with ST-segment elevationThe Task Force for the management of acute myocardial infarction in patients presenting with ST-segment elevation of the European Society. Eur Heart J. 2018 Jan 7;39(2):119–77.
2. Jern Sverker. Klinisk EKG-Diagnostik. 2010;
3. Roffi M, Patrono C, Collet J-P, Mueller C, Valgimigli M, Andreotti F, et al. 2015 ESC Guidelines for the management of acute coronary syndromes in patients presenting without persistent ST-segment elevationTask Force for the Management of Acute Coronary Syndromes in Patients Presenting without Persistent ST-Segment Elevation of th. Eur Heart J. 2016 Jan 14;37(3):267–315.
4. Rhinehardt J, Brady WJ, Perron AD, Mattu A. Electrocardiographic manifestations of Wellens' syndrome. Am J Emerg Med. 2002 Nov 1;20(7):638–43.
5. Douglas P. Zipes, Peter Libby, Robert O. Bonow, Douglas L. Mann, Gordon F. Tomaselli and EB. Braunwald's Heart Disease: A Textbook of Cardiovascular Medicine 11th edition. 2019;
6. Surawicz B, Childers R, Deal BJ, Gettes LS. AHA/ACCF/HRS Recommendations for the Standardization and Interpretation of the Electrocardiogram. Part III: Intraventricular Conduction Disturbances A Scientific Statement From the American Heart Association Electrocardiography and Arrhythmias Committee, . J Am Coll Cardiol. 2009 Mar 17;53(11):976–81.
