# Supplementary material for: Prehospital stratification in acute chest pain patient into high risk and low risk by emergency medical service: a prospective cohort study
Source: BMJ Open. 2021 Apr 15;11(4):e044938. doi: 10.1136/bmjopen-2020-044938 (PMC8055143; doi:10.1136/bmjopen-2020-044938)
Supplement: Supplementary data [file bmjopen-2020-044938supp003.pdf]

Supplemental material 3 - Description of study sample

| | | Sex | | Age group | | |
|---|---|---|---|---|---|---|
| | All n (%) | Men n (%) | Women n (%) | ≤50 n (%) | 51-64 n (%) | ≥65 n (%) |
| | 2917 (100) | 1465 (50.2) | 1452 (49.8) | 476 (16.3) | 539 (18.5) | 1902 (65.2) |
| Mean age (SD) | 69 (17) | 67 (18) | 70 (17) | 39 (9) | 58 (4) | 79 (8) |
| Median age (Q1-Q3) | 72 (58-82) | 70 (56-80) | 73 (60-83) | 41 (31-47) | 58 (55-62) | 79 (73-85) |
| Priority by EMD[a] | | | | | | |
| Priority 1 | 1836 (62.9) | 936 (63.9) | 900 (62.0) | 280 (55.5) | 232 (59.9) | 1233 (64.8) |
| Priority 2 | 1049 (36.0) | 513 (35) | 536 (36.9) | 189 (39.7) | 208 (38.6) | 652 (34.3) |
| Priority 3 | 32 (1.1) | 16 (1.1) | 16 (1.1) | 7 (1.5) | 8 (1.5) | 17 (0.9) |
| Priority by EMS-personnel[b] | | | | | | |
| Priority 1 | 311 (10.7) | 208 (14.2) | 103 (7.1) | 37 (7.8) | 68 (12.3) | 206 (10.8) |
| Priority 2 | 1969 (67.5) | 981 (67.0) | 988 (68) | 286 (60.1) | 355 (65.9) | 1328 (69.8) |
| Priority 3 | 325 (11.1) | 139 (9.5) | 186 (12.8) | 68 (14.3) | 63 (11.7) | 194 (10.2) |
| Priority according to RETTS[c] by EMS-personnel[b] | | | | | | |
| Red | 290 (9.9) | 187 (12.8) | 103 (7.1) | 32 (6.7) | 59 (10.9) | 199 (10.5) |
| Orange | 1628 (55.8) | 819 (55.9) | 809 (55.7) | 239 (50.2) | 291 (54.0) | 1098 (57.7) |
| Yellow | 887 (30.4) | 419 (28.6) | 468 (32.2) | 170 (35.7) | 170 (31.5) | 547 (28.8) |
| Green | 112 (3.8) | 40 (2.7) | 72 (5.0) | 35 (7.4) | 19 (3.5) | 58 (3.0) |
| Transport from primary care centre | 508 (17.4) | 286 (19.5) | 222 (15.3) | 77 (16.2) | 146 (27.1) | 285 (15.0) |
| Transport to primary care centre | 15 (0.5) | 8 (0.5) | 7 (0.5) | 3 (0.6) | 0 (0.0) | 12 (0.6) |
| Transported to hospital | 2600 (89.1) | 1324 (90.4) | 1276 (87.9) | 388 (81.5) | 488 (90.5) | 1724 (90.6) |
| Admitted to hospital (missing = 1) | 1409 (48.3) | 812 (55.4) | 597 (41.1) | 123 (25.8) | 235 (43.6) | 1051 (55.3) |
| Did not convey | 302 (10.4) | 133 (9.1) | 169 (11.6) | 85 (17.9) | 51 (9.5) | 166 (8.7) |
| EMS response time (minutes) | | | | | | |
| Median EMS dispatch to scene arrival (Q1-Q3) | 10 (6-15) | 9 (6-15) | 10 (6-16) | 10 (7-16) | 10 (6-17) | 9 (6-15) |
| Median EMS time at scene (Q1-Q3) | 25 (18-31) | 24 (18-30) | 25 (19-32) | 22 (16-27) | 22 (15-27) | 26 20-33) |
| Median transportation time (Q1-Q3) | 25 (13-34) | 25 (14-33) | 25 (12-35) | 25 (13-35) | 24 (13-32) | 25 13-34) |
| Median total EMS time (Q1-Q3) | 95 (73-118) | 95 (73-118) | 95 (73-118) | 89 (67-115) | 92 (70-113) | 97 75-120) |

[a]Emergency dispatch centre

[b]Emergency medical services

[c]Rapid Emergency Triage and Treatment System
