# Supplementary material for: Prehospital stratification in acute chest pain patient into high risk and low risk by emergency medical service: a prospective cohort study
Source: BMJ Open. 2021 Apr 15;11(4):e044938. doi: 10.1136/bmjopen-2020-044938 (PMC8055143; doi:10.1136/bmjopen-2020-044938)
Supplement: Supplementary data [file bmjopen-2020-044938supp004.pdf]

Supplemental material 4 - Prediction of high-risk conditions, univariate analyses

		Total with variable % (n)	Patients with high-risk condition % (n)	Patients without high-risk condition % (n)	High-risk vs without high-risk, p-value[a]	Odds Ratio	Confidence Interval, 95 %
All		2917 (100)	16.0 (467)	84.0 (2450)			
	Men	50.2 (1465)	63.8 (298)	47.6 (1167)	<0.001***	1.93	1.58-2.38
	Women	49.8 (1452)	36.2 (169)	52.4 (1283)			
Age		72 (median)	75 (median)	71 (median)	<0.001***	1.02	1.01-1.03
Age-group							
	Age ≤50	16.3 (476)	6.2 (29)	18.2 (447)			
	Age 51-64	18.5 (539)	17.1 (80)	18.7 (459)	<0.001***	2.68	1.72-4.19
	Age ≥65	65.2 (1902)	76.7 (358)	63.0 (1544)	<0.001***	3.57	2.41-5.29
Previous history of the following according to medical record							
Acute coronary syndrome (AMI, unstable angina)		29.3 (856)	27.8 (130)	29.6 (726)	0.435	0.91	0.74-1.14
Chronic obstructive pulmonary disease		10.5 (307)	7.5 (35)	11.2 (272)	0.021*	0.64	0.45-0.94
Angina pectoris		20.0 (582)	16.9 (79)	20.5 (503)	0.074	0.78	0.61-1.02
Hypertension		58.1 (1696)	63.2 (295)	57.2 (1401)	0.016*	1.28	1.05-1.58
Heart failure		20.6 (600)	18.0 (84)	21.1 (516)	0.132	0.82	0.64-1.06
Diabetes mellitus (type I and II)		19.8 (578)	23.6 (110)	19.1 (468)	0.027*	1.30	1.03-1.65
Stroke		8.2 (240)	7.9 (37)	8.3 (203)	0.794	0.95	0.66-1.37
Kidney disease		12.0 (350)	12.6 (59)	11.9 (291)	0.645	1.07	0.80-1.45
Atrial fibrillation/flutter		26.3 (767)	15.8 (74)	28.3 (693)	<0.001***	0.47	0.37-0.62
Rheumatism		2.3 (66)	1.5 (7)	2.4 (59)	0.230	0.61	0.28-1.36
Cancer (any type)		17.2 (502)	18.6 (87)	16.9 (415)	0.375	1.12	0.87-1.45
Psychiatric diagnosis (any type)		38.9 (1136)	27.4 (128)	41.1 (1008)	<0.001***	0.54	0.43-0.67

**Supplemental material 4 (continues) - Prediction of high-risk conditions, univariate analyses**						
	Total with variable % (n)	Patients with high-risk condition % (n)	Patients without high-risk condition % (n)	High-risk vs without high-risk, p-value[a]	Odds Ratio	Confidence Interval, 95 %
Vital signs on EMS arrival (missing)						
Breathing rate ≤8 breaths/min (22)	0 (0)	0 (0)	0 (0)	–	–	–
Breathing rate ≥25 breaths/min (22)	10.2 (297)	14.7 (68)	9.4 (229)	0.001**	1.65	1.24-2.22
Oxygen saturation ≤91 % (8)	3.8 (110)	6.4 (30)	3.3 (80)	0.001**	2.02	1.32-3.12
Heart rate ≤40 beats/min (18)	0.2 (7)	0.9 (4)	0.1 (3)	–	–	–
Heart rate ≥131 beats/min (18)	3.1 (91)	2.4 (11)	3.3 (80)	0.295	0.71	0.38-1.35
Systolic blood pressure ≤90 mmHg (22)	1.0 (29)	1.5 (7)	0.9 (22)	0.230	1.69	0.72-3.98
Systolic blood pressure ≥220 mmHg (22)	1.0 (28)	0.4 (2)	1.1 (26)	–	–	–
Decreased level of consciousness or new confusion (68)	0.9 (25)	1.5 (7)	0.7 (18)	0.114	2.03	0.84-4.89
Body temperature ≤35.0 (110)	0.2 (6)	0.5 (2)	0.2 (4)	–	–	–
Body temperature >38.0 (110)	2.3 (67)	2.0 (9)	2.5 (58)	0.599	0.82	0.41-1.68
Symptoms on EMS arrival (missing)						
Pale (565)	16.4 (386)	29.1 (111)	14.0 (275)	<0.001***	2.52	1.96-3.26
Clammy (565)	8.7 (204)	14.4 (55)	7.6 (149)	<0.001***	2.05	1.48-2.86
Nausea (576)	27.1 (635)	26.8 (101)	27.2 (534)	0.873	0.98	0.76-1.26
Vomiting (576)	7.0 (165)	7.4 (28)	7.0 (137)	0.754	1.07	0.70-1.63
Affected breathing according to patient (596)	44.6 (1040)	41.9 (158)	45.1 (882)	0.248	0.87	0.70-1.10
Pain intensity according to Numeric Rating Scale. NRS 0-10 (415)						
0	20.3 (509)	17.4 (71)	20.9 (438)	<0.001***		
1	3.8 (94)	3.2 (13)	3.9 (81)	0.976	0.99	0.52-1.87
2	11.5 (287)	9.8 (40)	11.8 (247)	0.996	0.99	0.66-1.52
3	7.6 (189)	8.6 (35)	7.4 (154)	0.136	1.40	0.90-2.19
4	8.1 (202)	6.8 (28)	8.3 (174)	0.976	0.99	0.62-1.59
5	16.7 (418)	15.4 (63)	17.0 (355)	0.629	1.09	0.76-1.58
6	8.3 (207)	6.8 (28)	8.6 (179)	0.882	0.96	0.60-1.56
7	10.5 (263)	12.2 (50)	10.2 (213)	0.068	1.44	0.97-2.15
8	8.2 (206)	9.8 (40)	7.9 (166)	0.069	1.48	0.97-2.28
9	2.6 (64)	4.9 (20)	2.1 (44)	0.001**	2.80	1.56-5.03
10	2.5 (63)	5.1 (21)	2.4 (42)	<0.001***	3.08	1.73-5.51

Supplemental material 4 (continues) - Prediction of high-risk conditions, univariate analyses

	Total with variable % (n)	Patients with high-risk condition % (n)	Patients without high-risk condition % (n)	High-risk vs without high-risk, p-value[a]	Odds Ratio	Confidence Interval, 95 %
Time elapsed since pain onset >3 hours (1007)	45.2 (863)	40.3 (128)	46.2 (735)	0.053	0.78	0.62-1.00
Debut						
Debut during activity (752)	22.1 (479)	32.8 (116)	20.0 (363)	<0.001***	1.94	1.51-2.50
Debut while resting (752)	65.5 (1419)	59.6 (211)	66.7 (1208)	0.010*	0.73	0.58-0.93
Debut while sleeping (752)	15.8 (342)	11.3 (40)	16.7 (302)	0.012*	0.63	0.45-0.91
Sudden debut, within seconds (875)	35.7 (729)	33.0 (115)	36.3 (614)	0.239	0.86	0.68-1.10
Quick debut, within minutes (875)	35.2 (718)	42.7 (149)	33.6 (569)	0.001**	1.47	1.16-1.86
Slow debut, within hours (875)	29.1 (595)	24.4 (85)	30.1 (510)	0.031*	0.74	0.57-0.97
Constant pain (732)	55.5 (1212)	61.8 (235)	49.9 (977)	<0.001***	1.66	1.31-2.11
Fluctuating pain (732)	40.4 (883)	30.5 (116)	39.2 (767)	0.001**	0.66	0.52-0.84
Pain aggravating over time (732)	10.8 (237)	8.7 (33)	10.4 (204)	0.279	0.80	0.55-1.19
Pain in other parts of the body (1197)						
Head	2.5 (43)	0.7 (2)	2.9 (41)	–	–	–
Throat	10.3 (177)	8.2 (24)	10.7 (153)	0.202	0.74	0.48-1.17
Jaw	5.3 (92)	7.5 (22)	4.9 (70)	0.071	1.58	0.96-2.58
Neck	2.5 (43)	2.7 (8)	2.5 (35)	0.773	1.12	0.56-2.44
Between scapulars	2.2 (37)	4.1 (12)	1.8 (25)	0.014*	2.40	1.19-4.84
Back	15.2 (261)	15.4 (45)	15.1 (216)	0.902	1.02	0.72-1.45
Left shoulder	8.5 (147)	7.9 (23)	8.7 (124)	0.653	0.89	0.57-1.43
Right shoulder	4.2 (72)	6.2 (18)	3.8 (54)	0.067	1.67	0.97-2.89
Left arm	24.0 (412)	33.6 (98)	22.0 (314)	<0.001***	1.79	1.36-2.36
Right arm	8.4 (145)	16.4 (48)	6.8 (97)	<0.001***	2.69	1.86-3.92
Left hand	1.0 (17)	0.7 (2)	1.1 (15)	–	–	–
Right hand	0.3 (6)	0.3 (1)	0.4 (5)	–	–	–
Stomach	7.0 (121)	4.8 (14)	7.5 (107)	0.103	0.62	0.35-1.10
Left leg	1.7 (29)	0.7 (2)	1.9 (27)	–	–	–
Right leg	1.4 (24)	0.0 (0)	1.7 (24)	–	–	–
No other pain	39.3 (676)	36.3 (106)	39.9 (570)	0.249	0.85	0.66-1.11

Supplemental material 4 (continues) - Prediction of high-risk conditions, univariate analyses

		Total with variable % (n)	Patients with high-risk condition % (n)	Patients without high-risk condition % (n)	High-risk vs without high-risk, p-value[a]	Odds Ratio	Confidence Interval, 95 %
Pain quality (1175)							
	Band-shaped	3.3 (58)	5.2 (15)	3.0 (43)	0.051	1.81	1.00-3.32
	Burning	4.4 (76)	4.5 (13)	4.3 (63)	0.869	1.05	0.57-1.94
	Stabbing	9.7 (169)	4.2 (12)	10.8 (157)	0.001**	0.36	0.20-0.66
	Cramping	8.7 (151)	9.8 (28)	8.4 (123)	0.461	1.17	0.76-1.81
	Dull pain	13.9 (242)	13.3 (38)	14.0 (204)	0.746	0.94	0.65-1.36
	Fells like something is on the chest	0.7 (12)	1.0 (3)	0.6 (9)	–	–	–
	Discomfort	10.2 (178)	8.7 (25)	10.5 (153)	0.368	0.81	0.52-1.27
	Tingling/Stinging	5.7 (99)	1.4(4)	6.5 (95)	–	–	–
	Swaying	1.7 (30)	1.4 (4)	1.8 (26)	–	–	–
	Pressuring	57.9 (1008)	63.6 (182)	56.7 (826)	0.031*	1.33	1.03-1.74
	Heaviness	1.0 (17)	1.4 (4)	0.9 (13)	–	–	–
	Aching	2.4 (41)	3.1 (9)	2.2 (32)	0.336	1.44	0.68-3.06
Chest pain localization (640)							
	Central pain	53.4 (1215)	64.5 (240)	51.2 (975)	<0.001***	1.73	1.38-2.18
	Left side of chest	35.5 (809)	28.0 (104)	37.0 (705)	0.001**	0.66	0.52-0.84
	Right side of chest	5.1 (116)	2.4 (9)	5.6 (107)	0.013*	0.41	0.21-0.83
	Upper part of chest	6.7 (152)	7.3 (27)	6.6 (125)	0.623	1.11	0.72-1.72
	Lower part of chest	9.0 (204)	5.9 (22)	9.6 (182)	0.026*	0.59	0.38-0.94
	All over the chest	11.8 (269)	12.6 (47)	11.7 (222)	0.592	1.09	0.78-1.54
Size of area affected by pain (794)							
	Two inch diameter	10.7 (228)	5.1 (18)	11.9 (210)	<0.001***	0.39	0.24-0.65
	Size of patient's palm	58.4 (1240)	61.0 (216)	57.9 (1024)	0.275	1.13	0.90-1.44
	Entire chest	30.9 (655)	33.9 (120)	30.2 (535)	0.174	1.18	0.93-1.51
Palpation tenderness (655)		22.3 (505)	15.8 (58)	23.6 (447)	0.001**	0.60	0.45-0.82
Pain affected by movement (719)		17.0 (373)	11.5 (41)	18.0 (332)	0.003**	0.59	0.42-0.83
Pain affected by breathing (692)		25.8 (573)	17.3 (63)	27.4 (510)	<0.001***	0.55	0.41-0.74

Supplemental material 4 (continues) - Prediction of high-risk conditions, univariate analyses

	Total with variable % (n)	Patients with high-risk condition % (n)	Patients without high-risk condition % (n)	High-risk vs without high-risk, p-value[a]	Odds Ratio	Confidence Interval, 95 %
Troponin T (missing)						
High-sensitive Troponin T, cut-off >14 ng/L (1416)	12 (median)	33 (median)	11 (median)	<0.001***	1.01	1.01-1.02
Troponin T adjusted to Roche Cobas h232 (1416)						
Tnt <50 ng/L	88.1 (1323)	62.9 (161)	93.3 (1162)	<0.001***		
Tnt 51-100 ng/L	6.6 (99)	15.6 (40)	4.7 (59)	<0.001***	4.89	3.17-7.55
Tnt 101-1000 ng/L	4.5 (67)	17.2 (44)	1.8 (23)	<0.001***	13.8	8.12-23.47
Tnt >1000 ng/L	0.8 (12)	4.3 (11)	0.1 (1)	–	–	–
ECG (missing[b])						
ECG transmitted to hospital	95.5 (2785)	97.2 (454)	95.1 (2331)	0.051	1.78	1.00-3.19
Sinus Rhythm, SR (235)	83.8 (2248)	84.7 (377)	83.6 (1871)	0.572	1.08	0.82-1.44
Sinus Bradycardia (235)	1.3 (34)	1.1 (5)	1.3 (29)	0.766	0.86	0.33-2.25
Sinus Tachycardia (235)	8.9 (240)	12.4 (55)	8.3 (185)	0.006	1.56	1.14-2.15
Supraventricular Tachycardia, SVT (235)	0.5 (14)	0.2 (1)	0.6 (3)	–	–	–
Atrial Fibrillation/Flutter, AF (235)	14.3 (383)	13.9 (62)	14.3 (321)	0.818	0.96	0.72-1.30
Ventricular Tachycardia, VT (235)	0.0 (1)	0.0 (0)	0.2 (1)	–	–	–
Atrial Pacing (235)	1.0 (29)	0.4 (2)	1.2 (27)	–	–	–
Ventricular Pacing (150)	3.1 (85)	1.8 (8)	3.3 (77)	0.083	0.52	0.25-1.09
AV-block II type 1 (235)	0.0 (1)	0.2 (1)	0.0 (0)	–	–	–
AV-block II type 2 (235)	0.0 (1)	0.0 (0)	0.0 (1)	–	–	–
AV-Block III (235)	0.1 (2)	0.4 (2)	0.0 (0)	–	–	–
ST-Elevation (235)	6.1 (164)	22.9 (102)	2.8 (62)	<0.001***	10.4	7.46-14.59
ST-Depression (235)	7.6 (204)	23.8 (106)	4.4 (98)	<0.001***	5.82	5.07-9.19
T-wave Inversion (235)	14.5 (388)	22.7 (101)	12.8 (287)	<0.001***	1.99	1.55-2.57
Q-wave (235)	4.8 (130)	6.5 (29)	4.5 (101)	0.074	1.47	0.96-2.26
Premature Ventricular Contraction, PVC (235)	5.5 (147)	7.9 (35)	5.0 (112)	0.016*	1.62	1.09-2.40
Premature Atrial Contractions, PAC (235)	3.5 (94)	6.1 (27)	3.0 (67)	0.002**	2.09	1.32-3.31
Left Bundle Branch Block, LBBB (235)	6.3 (168)	6.5 (29)	6.2 (139)	0.810	1.05	0.70-1.59
Right Bundle Branch Block, RBBB (235)	6.3 (170)	9.2 (41)	5.8 (129)	0.007**	1.65	1.15-2.39
Sinus rhythm and none of abnormalities above (150)	44.6 (1233)	26.5 (120)	48.1 (1113)	<0.001***	0.38	0.31-0.49
QRS-duration (150)	92 (median)	96 (median)	92 (median)	<0.001***	1.00	1.01-1.01
Uninterpretable ECG (132)	0.6 (18)	0.2 (1)	0.7 (17)	–	–	–

Supplemental material 4 (continues) - Prediction of high-risk conditions, univariate analyses

*p < 0.05
**p < 0.01
***p < 0.001
[a]Logistic regression
[b]When ventricular pacing or uninterpretable remaining ECG interpretation has been treated as missing.
