# Supplementary material for: Prehospital stratification in acute chest pain patient into high risk and low risk by emergency medical service: a prospective cohort study
Source: BMJ Open. 2021 Apr 15;11(4):e044938. doi: 10.1136/bmjopen-2020-044938 (PMC8055143; doi:10.1136/bmjopen-2020-044938)
Supplement: Supplementary data [file bmjopen-2020-044938supp005.pdf]

Supplemental material 5 - Prediction of low-risk conditions, univariate analyses

		Total with variable % (n)	Patients with low-risk condition % (n)	Patients without low-risk condition % (n)	Low-risk vs without low-risk, p-value[a]	Odds Ratio	Confidence Interval, 95 %
All		2917 (100)	68.4 (1995)	31.6 (922)			
	Men	50.2 (1465)	46.2 (922)	41.1 (379)	<0.001***	0.60	0.51-0.70
	Women	49.8 (1452)	53.8 (1073)	58.9 (543)			
Age		72 (median)	70 (median)	76 (median)	<0.001***	0.98	0.97-0.98
Age-group							
	Age ≤50	16.3 (476)	20.4 (406)	7.6 (70)			
	Age 51-64	18.5 (539)	20.1 (401)	15.0 (138)	<0.001***	0.50	0.36-0.69
	Age ≥65	65.2 (1902)	59.5 (1188)	77.4 (714)	<0.001***	0.29	0.22-0.38
Previous history of the following according to medical record							
Acute coronary syndrome (AMI, unstable angina)		29.3 (856)	29.0 (578)	30.2 (278)	0.515	0.95	0.80-1.12
Chronic obstructive pulmonary disease		10.5 (307)	9.7 (194)	12.3 (113)	0.039*	0.77	0.60-0.99
Angina pectoris		20.0 (582)	20.5 (409)	18.8 (173)	0.275	1.12	0.92-1.36
Hypertension		58.1 (1696)	55.6 (1110)	63.6 (586)	<0.001***	0.72	0.61-0.84
Heart failure		20.6 (600)	18.6 (371)	24.8 (229)	<0.001***	0.69	0.57-0.83
Diabetes mellitus (type I and II)		19.8 (578)	19.4 (388)	20.6 (190)	0.465	0.93	0.77-1.13
Stroke		8.2 (240)	7.5 (150)	9.8 (90)	0.041*	0.75	0.57-0.99
Kidney disease		12.0 (350)	11.0 (219)	14.2 (131)	0.013*	0.75	0.59-0.94
Atrial fibrillation/flutter		26.3 (767)	25.0 (498)	29.2 (269)	0.016*	0.81	0.68-0.96
Rheumatism		2.3 (66)	2.3 (46)	2.2 (20)	0.818	1.06	0.63-1.81
Cancer (any type)		17.2 (502)	15.2 (303)	21.6 (199)	<0.001***	0.65	0.53-0.79
Psychiatric diagnosis (any type)		38.9 (1136)	42.5 (848)	31.2 (288)	<0.001***	1.63	1.38-1.92

Supplemental material 5 (continues) - Prediction of low-risk conditions, univariate analyses

	Total with variable % (n)	Patients with low-risk condition % (n)	Patients without low-risk condition % (n)	Low-risk vs without low-risk, p-value[a]	Odds Ratio	Confidence Interval, 95 %
Vital signs on EMS arrival (missing)						
Breathing rate ≤8 breaths/min (22)	0 (0)	0 (0)	0 (0)	–	–	–
Breathing rate ≥25 breaths/min (22)	10.2 (297)	6.2 (122)	19.1 (175)	<0.001***	0.28	0.22-0.36
Oxygen saturation ≤91 % (8)	3.8 (110)	1.2 (23)	9.4 (87)	<0.001***	0.11	0.07-0.18
Heart rate ≤40 beats/min (18)	0.2 (7)	0.1 (1)	0.7 (6)	–	–	–
Heart rate ≥131 beats/min (18)	3.1 (91)	0.9 (18)	7.9 (73)	<0.001***	0.11	0.06-0.18
Systolic blood pressure ≤90 mmHg (22)	1.0 (29)	0.6 (12)	1.9 (17)	0.003**	0.32	0.15-0.67
Systolic blood pressure ≥220 mmHg (22)	1.0 (28)	0.8 (16)	1.3 (12)	0.198	0.61	0.29-1.30
Decreased level of consciousness or new confusion (68)	0.9 (25)	0.5 (9)	1.8 (16)	0.001**	0.26	0.12-0.60
Body temperature ≤35.0 (110)	0.2 (6)	0.2 (3)	0.3 (3)	–	–	–
Body temperature >38.0 (110)	2.3 (67)	1.4 (27)	4.5 (40)	<0.001***	0.30	0.18-0.49
Symptoms on EMS arrival (missing)						
Pale (565)	16.4 (386)	11.5 (182)	26.6 (204)	<0.001***	0.36	0.29-0.45
Clammy (565)	8.7 (204)	6.1 (97)	14.0 (107)	<0.001***	0.40	0.30-0.54
Nausea (576)	27.1 (635)	26.6 (421)	28.2 (214)	0.405	0.92	0.76-1.12
Vomiting (576)	7.0 (165)	5.8 (92)	9.6 (73)	0.001**	0.58	0.42-0.80
Affected breathing according to patient (596)	44.6 (1040)	40.7 (640)	52.6 (400)	<0.001***	0.62	0.52-0.74
Pain intensity according to Numeric Rating Scale. NRS 0-10 (415)						
0	20.3 (509)	21.8 (367)	17.4 (142)	0,004*		
1	3.8 (94)	4.3 (72)	2.7 (22)	0.369	1.27	0.76-2.12
2	11.5 (287)	11.9 (200)	10.7 (87)	0.470	0.89	0.65-1.22
3	7.6 (189)	7.3 (123)	8.1 (66)	0.072	0.72	0.51-1.03
4	8.1 (202)	8.7 (147)	6.7 (55)	0.857	1.03	0.72-1.49
5	16.7 (418)	16.1 (272)	17.9 (146)	0.022*	0.72	0.55-0.95
6	8.3 (207)	8.3 (140)	8.2 (67)	0.233	0.81	0.57-1.15
7	10.5 (263)	10.0 (169)	11.5 (94)	0.025*	0.70	0.51-0.96
8	8.2 (206)	7.5 (126)	9.8 (80)	0.004**	0.61	0.43-0.86
9	2.6 (64)	2.2 (37)	3.3 (27)	0.020*	0.53	0.31-0.90
10	2.5 (63)	2.0 (34)	3.6 (29)	0.004**	0.45	0.27-0.77

Supplemental material 5 (continues) - Prediction of low-risk conditions, univariate analyses

	Total with variable % (n)	Patients with low-risk condition % (n)	Patients without low-risk condition % (n)	Low-risk vs without low-risk, p-value[a]	Odds Ratio	Confidence Interval, 95 %
Time elapsed since pain onset >3 hours (1007)	45.2 (863)	45.9 (592)	43.8 (271)	0.394	1.09	0.90-1.32
Debut						
Debut during activity (752)	22.1 (479)	21.0 (306)	24.4 (173)	0.071	0.82	0.67-1.02
Debut while resting (752)	65.5 (1419)	65.6 (956)	65.4 (463)	0.920	1.01	0.84-1.22
Debut while sleeping (752)	15.8 (342)	16.9 (246)	13.6 (96)	0.047*	1.30	1.00-1.67
Sudden debut, within seconds (875)	35.7 (729)	37.5 (513)	32.1 (216)	0.017*	1.27	1.04-1.54
Quick debut, within minutes (875)	35.2 (718)	33.3 (456)	38.9 (262)	0.012*	0.78	0.65-0.95
Slow debut, within hours (875)	29.1 (595)	29.2 (400)	29.0 (195)	0.909	1.01	0.83-1.24
Constant pain (732)	55.5 (1212)	52.9 (778)	60.9 (434)	<0.001***	0.72	0.60-0.86
Fluctuating pain (732)	40.4 (883)	42.7 (629)	35.6 (254)	0.002**	1.35	1.12-1.62
Pain aggravating over time (732)	10.8 (237)	11.0 (162)	10.5 (75)	0.732	1.05	0.79-1.41
Pain in other parts of the body (1197)						
Head	2.5 (43)	3.0 (35)	1.4 (8)	0.051	2.16	0.10-4.70
Throat	10.3 (177)	10.5 (121)	9.9 (56)	0.743	1.06	0.76-1.48
Jaw	5.3 (92)	5.0 (58)	6.0 (34)	0.375	0.82	0.53-1.27
Neck	2.5 (43)	2.2 (26)	3.0 (17)	0.337	0.74	0.40-1.37
Between scapulars	2.2 (37)	1.9 (22)	2.7 (15)	0.308	0.71	0.36-1.38
Back	15.2 (261)	15.5 (179)	14.6 (82)	0.623	1.07	0.81-1.43
Left shoulder	8.5 (147)	8.6 (99)	8.5 (48)	0.983	1.00	0.70-1.44
Right shoulder	4.2 (72)	3.5 (41)	5.5 (31)	0.580	0.63	0.39-1.02
Left arm	24.0 (412)	22.9 (265)	26.1 (147)	0.144	0.84	0.67-1.06
Right arm	8.4 (145)	6.2 (72)	13.0 (73)	<0.001***	0.45	0.32-0.63
Left hand	1.0 (17)	1.2 (14)	0.5 (3)	–	–	–
Right hand	0.3 (6)	0.3 (4)	0.4 (2)	–	–	–
Stomach	7.0 (121)	6.9 (80)	7.3 (41)	0.779	0.95	0.54-1.40
Left leg	1.7 (29)	2.0 (23)	1.1 (6)	0.170	1.88	0.76-4.65
Right leg	1.4 (24)	1.8 (21)	0.5 (3)	–	–	–
No other pain	39.3 (676)	39.8 (461)	38.2 (215)	0.509	1.07	0.87-1.32

Supplemental material 5 (continues) - Prediction of low-risk conditions, univariate analyses

		Total with variable % (n)	Patients with low-risk condition % (n)	Patients without low-risk condition % (n)	Low-risk vs without low-risk, p-value[a]	Odds Ratio	Confidence Interval, 95 %
Pain quality (1175)							
	Band-shaped	3.3 (58)	3.0 (35)	4.0 (23)	0.250	0.73	0.43-1.25
	Burning	4.4 (76)	4.8 (56)	3.5 (20)	0.229	1.38	0.82-2.32
	Stabbing	9.7 (169)	10.9 (128)	7.2 (41)	0.015*	1.58	1.09-2.28
	Cramping	8.7 (151)	9.0 (1255)	8.1 (46)	0.547	1.12	0.78-1.61
	Dull pain	13.9 (242)	13.8 (162)	14.1 (80)	0.888	0.98	0.73-1.31
	Fells like something is on the chest	0.7 (12)	0.6 (7)	0.9 (5)	0.507	0.68	0.21-2.14
	Discomfort	10.2 (178)	10.7 (125)	9.3 (53)	0.386	1.16	0.83-1.63
	Tingling/Stinging	5.7 (99)	7.1 (83)	2.8 (16)	<0.001***	2.63	1.53-4.54
	Swaying	1.7 (30)	1.8 (21)	1.6 (9)	0.754	1.13	0.52-2.49
	Pressuring	57.9 (1008)	55.8 (654)	62.2 (354)	0.011*	0.77	0.62-0.94
	Heaviness	1.0 (17)	0.9 (11)	1.1 (6)	0.816	0.89	0.33-2.41
	Aching	2.4 (41)	2.0 (24)	3.0 (17)	0.227	0.69	0.36-1.27
Chest pain localization (640)							
	Central pain	53.4 (1215)	50.4 (776)	59.5 (439)	<0.001***	0.69	0.58-0.83
	Left side of chest	35.5 (809)	39.3 (605)	27.6 (204)	<0.001***	1.70	1.40-2.05
	Right side of chest	5.1 (116)	5.7 (87)	3.9 (29)	0.082	1.47	0.95-2.25
	Upper part of chest	6.7 (152)	6.8 (104)	6.5 (48)	0.821	1.01	0.93-1.10
	Lower part of chest	9.0 (204)	9.0 (138)	8.9 (66)	0.985	1.00	0.74-1.36
	All over the chest	11.8 (269)	11.0 (169)	13.6 (100)	0.076	0.79	0.60-1.03
Size of area affected by pain (794)							
	Two inch diameter	10.7 (228)	12.2 (175)	7.7 (53)	0.002**	1.68	1.22-2.32
	Size of patient's palm	58.4 (1240)	59.3 (848)	56.6 (392)	0.253	1.11	0.93-1.34
	Entire chest	30.9 (655)	28.5 (408)	35.7 (247)	0.001**	0.72	0.59-0.87
Palpation tenderness (655)		22.3 (505)	24.3 (371)	18.3 (134)	0.001**	1.44	1.15-1.79
Pain affected by movement (719)		17.0 (373)	18.7 (277)	13.4 (96)	0.002**	1.49	1.16-1.91
Pain affected by breathing (692)		25.8 (573)	26.5 (398)	24.2 (175)	0.258	1.13	0.92-1.38

Supplemental material 5 (continues) - Prediction of low-risk conditions, univariate analyses	Total with variable % (n)	Patients with low-risk condition % (n)	Patients without low-risk condition % (n)	Low-risk vs without low-risk, p-value[a]	Odds Ratio	Confidence Interval, 95 %
Troponin T (missing)						
High-sensitive Troponin T, cut-off >14 ng/L (1416)	12 (median)	9 (median)	25 (median)	<0.001***	0.98	0.98-0.98
Troponin T adjusted to Roche Cobas h232 (1416)					0.21	
Tnt <50 ng/L	88.1 (1323)	96.1 (932)	73.6 (391)	<0.001***		
Tnt 51-100 ng/L	6.6 (99)	3.2 (31)	12.8 (68)	<0.001***	0.19	0.12-0.30
Tnt 101-1000 ng/L	4.5 (67)	0.6 (6)	11.5 (61)	0.002**	0.04	0.02-0.10
Tnt >1000 ng/L	0.8 (12)	0.1 (1)	2.1 (11)	–	–	–
ECG (missing[b])						
ECG transmitted to hospital	95.5 (2785)	95.0 (1895)	96.5 (890)	0.064	0.68	0.45-1.02
Sinus Rhythm, SR (235)	83.8 (2248)	88.7 (1622)	73.3 (626)	<0.001***	2.87	2.33-3.54
Sinus Bradycardia (235)	1.3 (34)	1.3 (23)	1.3 (11)	0.949	0.98	0.47-2.01
Sinus Tachycardia (235)	8.9 (240)	7.3 (133)	12.5 (107	<0.001***	0.55	0.42-0.72
Supraventricular Tachycardia, SVT (235)	0.5 (14)	0.1 (1)	1.5 (13)	–	–	–
Atrial Fibrillation/Flutter, AF (235)	14.3 (383)	9.7 (177)	24.1 (206)	<0.001***	0.34	0.27-0.42
Ventricular Tachycardia, VT (235)	0.0 (1)	0.0 (0)	0.1 (1)	–	–	–
Atrial Pacing (235)	1.0 (29)	1.2 (22)	0.8 (7)	0.372	1.48	0.63-3.47
Ventricular Pacing (150)	3.1 (85)	3.0 (57)	3.2 (28)	0.830	0.95	0.60-1.51
AV-block II type 1 (235)	0.0 (1)	0.0 (0)	0.1 (1)	–	–	–
AV-block II type 2 (235)	0.0 (1)	0.1 (1)	0.0 (0)	–	–	–
AV-Block III (235)	0.1 (2)	0 (0.0)	0.2 (2)	–	–	–
ST-Elevation (235)	6.1 (164)	2.2 (41)	14.4 (123)	<0.001***	0.14	0.10-0.20
ST-Depression (235)	7.6 (204)	2.4 (44)	18.7 (160)	<0.001***	0.11	0.08-0.15
T-wave Inversion (235)	14.5 (388)	11.9 (217)	20.0 (171)	<0.001***	0.54	0.43-0.67
Q-wave (235)	4.8 (130)	4.9 (89)	4.8 (41)	0.939	1.02	0.70-1.48
Premature Ventricular Contraction, PVC (235)	5.5 (147)	5.5 (147)	6.6 (56)	0.095	0.75	0.53-1.05
Premature Atrial Contractions, PAC (235)	3.5 (94)	2.9 (53)	4.8 (41)	0.014*	0.59	0.39-0.90
Left Bundle Branch Block, LBBB (235)	6.3 (168)	5.2 (95)	8.5 (73)	0.001**	0.59	0.43-0.81
Right Bundle Branch Block, RBBB (235)	6.3 (170)	5.1 (93)	9.0 (77)	<0.001***	0.54	0.40-0.74
Sinus rhythm and none of abnormalities above (150)	44.6 (1233)	54.0 (1017)	24.5 (216)	<0.001***	3.61	3.02-4.32
QRS-duration (150)	92 (median)	92 (median)	96 (median)	<0.001***	0.99	0.99-0.99
Uninterpretable ECG (132)	0.6 (18)	0.5 (10)	0.9 (8)	0.260	0.59	0.23-1.49

Supplemental material 5 (continues) - Prediction of low-risk conditions, univariate analyses

*p < 0.05
**p < 0.01
***p < 0.001
[a]Logistic regression
[b]When ventricular pacing or uninterpretable remaining ECG interpretation has been treated as missing.
