# Supplementary material for: Prehospital stratification in acute chest pain patient into high risk and low risk by emergency medical service: a prospective cohort study
Source: BMJ Open. 2021 Apr 15;11(4):e044938. doi: 10.1136/bmjopen-2020-044938 (PMC8055143; doi:10.1136/bmjopen-2020-044938)
Supplement: Author's manuscript [file bmjopen-2020-044938.draft_revisions.pdf]

BMJ Open is committed to open peer review. As part of this commitment we make the peer review history of every article we publish publicly available.

When an article is published we post the peer reviewers' comments and the authors' responses online. We also post the versions of the paper that were used during peer review. These are the versions that the peer review comments apply to.

The versions of the paper that follow are the versions that were submitted during the peer review process. They are not the versions of record or the final published versions. They should not be cited or distributed as the published version of this manuscript.

BMJ Open is an open access journal and the full, final, typeset and author-corrected version of record of the manuscript is available on our site with no access controls, subscription charges or pay-per-view fees (http://bmjopen.bmj.com).

If you have any questions on BMJ Open's open peer review process please email info.bmjopen@bmj.com

BMJ Open

Prehospital stratification in acute chest pain patient into high- and low-risk by emergency medical service – a prospective cohort study

Journal:	*BMJ Open*
Manuscript ID	bmjopen-2020-044938
Article Type:	Original research
Date Submitted by the Author:	17-Sep-2020
Complete List of Authors:	Wibring, Kristoffer; University of Gothenburg Institute of Health and Care Sciences,<br>Lingman, Markus; Sahlgrenska Academy, University of Gothenburg, Department of molecular and clinical Medicine/Cardiology; Halland Hospital, Region Halland<br>Herlitz, Johan; Faculty of Caring Science, Work Life and Social Welfare, University of Borås<br>Amin, Sinan; Halland Hospital, Region Halland<br>Bång , Angela; University of Gothenburg,
Keywords:	ACCIDENT & EMERGENCY MEDICINE, Myocardial infarction < CARDIOLOGY, Cardiology < INTERNAL MEDICINE

SCHOLARONE™
Manuscripts

I, the Submitting Author has the right to grant and does grant on behalf of all authors of the Work (as defined in the below author licence), an exclusive licence and/or a non-exclusive licence for contributions from authors who are: i) UK Crown employees; ii) where BMJ has agreed a CC-BY licence shall apply, and/or iii) in accordance with the terms applicable for US Federal Government officers or employees acting as part of their official duties; on a worldwide, perpetual, irrevocable, royalty-free basis to BMJ Publishing Group Ltd ("BMJ") its licensees and where the relevant Journal is co-owned by BMJ to the co-owners of the Journal, to publish the Work in this journal and any other BMJ products and to exploit all rights, as set out in our licence.

The Submitting Author accepts and understands that any supply made under these terms is made by BMJ to the Submitting Author unless you are acting as an employee on behalf of your employer or a postgraduate student of an affiliated institution which is paying any applicable article publishing charge ("APC") for Open Access articles. Where the Submitting Author wishes to make the Work available on an Open Access basis (and intends to pay the relevant APC), the terms of reuse of such Open Access shall be governed by a Creative Commons licence – details of these licences and which Creative Commons licence will apply to this Work are set out in our licence referred to above.

Other than as permitted in any relevant BMJ Author's Self Archiving Policies, I confirm this Work has not been accepted for publication elsewhere, is not being considered for publication elsewhere and does not duplicate material already published. I confirm all authors consent to publication of this Work and authorise the granting of this licence.

For peer review only

Prehospital stratification in acute chest pain patient into high- and low-risk by emergency medical service – a prospective cohort study

Authors
Kristoffer Wibring[1, 2] kristoffer.wibring@gu.se
Markus Lingman[3,4] markus.lingman@regionhalland.se
Johan Herlitz[5] johan.herlitz@hb.se
Sinan Amin[6] sinan.amin@regionhalland.se
Angela Bång[2] angela.bang@gu.se

1. Department of Ambulance and Prehospital Care, Region Halland, Sweden
2. Institute of Health and Care Sciences, Sahlgrenska Academy, University of Gothenburg, Göteborg, Sweden
3. Department of Molecular and Clinical Medicine/Cardiology, Institute of Medicine, Sahlgrenska Academy, University of Gothenburg, Göteborg, Sweden
4. Department of development, Halland Hospital, Halmstad, Sweden
5. The Prehospital Research Center Western Sweden, University of Borås, Borås, Sweden.
6. Department of cardiology, Halland Hospital, Varberg, Sweden

All authors takes responsibility for all aspects of the reliability and freedom from bias of the data presented and their discussed interpretation.

Corresponding author
Kristoffer Wibring
Ambulanssjukvården Kungsbacka
Varlabergsvägen 29
434 39 Kungsbacka
E-mail: kristoffer.wibring@gu.se
Phone: 0046701757679

For peer review only

For peer review only - http://bmjopen.bmj.com/site/about/guidelines.xhtml

ABSTRACT

Objectives: To describe contemporary characteristics and diagnoses in prehospital patients with chest pain and to identify factors suitable for the early recognition of high- and low-risk conditions.

Design: Prospective observational cohort study

Setting: Single centre study in a Swedish county EMS organisation.

Participants: Unselected inclusion of 2917 patients with chest pain contacting the emergency medical services due to chest pain during 2018.

Primary outcome measures: Low- or high-risk condition, i.e. occurrence of time-sensitive diagnosis on hospital discharge.

Results: Among these chest pain-related EMS missions, 68 % concerned patients with a low-risk condition without medical need of acute hospital treatment. Sixteen percent concerned patients with a high-risk condition in need of rapid transport to hospital care. Numerous variables with significant association with low- or high-risk conditions were found. In total high- and low-risk prediction models shared ten predictive variables of which ST-elevation and ST depression on the electrocardiogram and age>65 years were most important. Previously known risk factors such as history of acute coronary syndrome, diabetes and hypertension had no predictive value. Some aspects of the symptoms such as pain intensity, pain in the right arm and paleness did on the other hand appear to be helpful. The area under the receiving operating characteristic curve for prediction of low-risk candidates was 0.806 and for high-risk candidates 0.833.

Conclusions: A majority of patients with chest pain cared for by the EMS suffer from a low-risk condition and have no prognostic reason for acute hospital care given their diagnosis on hospital discharge. A smaller proportion has a high-risk condition and is in need of prompt specialist care. Building models with good accuracy for prehospital identification of these groups is possible. The use of risk stratification models will make a more personalised care possible with increased patient safety.

STRENGTHS AND LIMITATIONS OF THIS STUDY

- Unselected inclusion of a close to complete county population of patients with chest pain contacting the emergency medical services.
- Low rates of missing considering the prehospital nature of data.
- Well examined cohort including data on demographics, previous medical history, symptoms, vital signs, ECG, biochemical markers and diagnosis on discharge.
- **Some variables are entailed with high rates of missing when compared to studies conducted in the hospital setting.**
- **Single centre study reduce generalisability**

Keywords
Chest Pain; Emergency Medical Services; Acute Coronary Syndrome; Signs and Symptoms; Triage; Risk Assessment

For peer review only - http://bmjopen.bmj.com/site/about/guidelines.xhtml

BACKGROUND

This report is the first presenting quantitative results in a larger research project (the BRIAN research programme) on improved prehospital risk assessment of patients with chest pain.

Chest pain is one of the most common chief complaints when contacting the emergency medical services (EMS). About 10-15 percent of all EMS missions concern patients with chest pain.[1 2] The role of the EMS when caring for these patients has changed since the introduction of telemedicine solutions for transmission of electrocardiograms (ECG) and direct transport to percutaneous intervention (PCI) centres for patients with ST-elevation myocardial infarction (STEMI).[3] However, these cases constitute only three percent of all patients with chest pain and one third of all patients with acute myocardial infarction (AMI).[2]

Previous studies[4] report that 15 percent of all EMS patients with chest pain have a life-threatening condition. It has been suggested that a large proportion of the patients transported by the EMS due to chest pain could be cared for outside the hospital.[5] Thus we can conclude that patients seen by the EMS before arrival in hospital due to chest pain form a mixed group with different care needs. Guidelines have focused only to a limited extent on personalising the care of patients with chest pain of other origins than STEMI despite the fact that the proportion of patients with AMI has decreased in recent decades from 30 %[6] of all patients seen by the EMS due to chest pain to 10 %.[2 4]

A more differentiated and personalised care for these chest pain patients has the potential of improving efficiency and outcome. For example, patients with non-ST elevation myocardial infarction (NSTEMI) may benefit from by-passing hospitals without PCI capabilities and by-passing the ED for direct transport to cardiac care units (CCU).[7-10] Furthermore, the undifferentiated care of the large group of patients with chest pain contributes to the problematic crowding of EDs.[11 12] Waiting in the ED increases the risk of an adverse outcome for these patients.[13 14] Consistently transporting patients with low-risk conditions to the ED also entails large costs [15] and occupy limited EMS resources. Referral to other destinations than the ED or non-conveyance for low-risk patients could provide improvements in all these aspects.

Hospital research on risk stratification is extensive but research based on data acquired in the prehospital setting is limited.[16] It is important to base prehospital clinical practice on prehospital data since patients who are seen in the prehospital setting differ from those seen at the ED.[6 17-19] This difference might affect pre-test probabilities hence diminishing the validity of the models when applied in a new (prehospital) setting.

Previous research shows that prehospital risk assessment of patients with chest pain may be possible.[2 16 20 21] However, research is still sparse and often has methodological shortcomings, mainly regarding patient selection and using AMI as the primary endpoint. This results in the neglect of other high-risk conditions, such as pulmonary embolism or aortic dissection, and reduces the possibility to identify low-risk patients suitable for non-conveyance. A risk assessment tool for chest pain patients, to be used in the prehospital setting, has been called for in previous research.[22-24] Such a tool might reduce arbitrariness in patient assessment and excess utilisation of emergency care.

The present report is mainly descriptive and investigates contemporary patient characteristics, outcome and how outcome can be predicted by information accessible in the EMS context.

For peer review only - http://bmjopen.bmj.com/site/about/guidelines.xhtml

Future reports within this project will focus on how such information could be used and combined in a refined risk assessment tool to be used in the prehospital setting in order to stratify risks and refer patients with chest pain to an appropriate level of care.

Objectives

- To describe contemporary characteristics and diagnoses among prehospital patients with high/low risk conditions presenting with chest pain.
- To identify factors suitable for the early recognition of:
    - patients with time-sensitive conditions in need of immediate care (high-risk conditions)
    - patients with no medical need of hospital treatment, suitable for non-conveyance to hospital (low-risk conditions)
    - present data that can inform the development of a prediction tool

METHODS

Study population

This prospective cohort study was conducted in the county of Halland, Sweden. In all, 3,121 EMS missions were carried out in 2018 concerning patients, ≥18 years old, with a chief complaint of chest pain. All these missions were eligible for inclusion. After excluding patients declining to participate and patients who were lost to follow-up, 2,917 EMS missions were included in data analyses (Figure 1).

Healthcare system

The county of Halland covers an area of 5,500 km^2 and had 329,000 inhabitants in 2018. These are served by two emergency hospitals, including one with PCI capabilities. The EMS consists of eight ambulance stations with 19 ambulance vehicles. In 2018, a total of 30,672 missions were carried out by the EMS (inter-hospital site transports excluded). The EMS is staffed mainly by nurses. An ECG is routinely collected prehospitally and stored digitally using telecommunications.

Data collection

The unique personal identity number assigned to all inhabitants in Sweden made it possible to track each patient throughout the healthcare chain, from EMS mission to hospital discharge and beyond. A novel questionnaire containing fifteen items concerning patient symptoms (Additional file 1) was integrated in the digital journal system used by the EMS. The questionnaire was developed by the research group using the findings from two earlier reports within the research project [16 25] along with results and methodology of numerous other studies concerning patients with chest pain. By using electronic tablets, both the questionnaire and the EMS record were available at the bedside during the entire EMS mission.

The questionnaire (Supplemental file 1) contained items mainly focusing on the patients' pain narratives identifying onset, provocation/palliation, quality, radiation, severity etc. Intensity was measured using the Numerical Rating Scale (NRS) ranging from 0-10.[26] If the patient was not able to use the NRS, a Verbal Rating Scale (VRS) ranging from "no pain" to "unbearable pain" was used instead.[27] The answers from the VRS were then transformed into NRS values as follows: "no pain" = 0, "mild pain" = 2 "moderate pain" = 5, "severe pain = 7" and "unbearable pain" = 9.[28] The questionnaire also contained items regarding nausea/vomiting, dyspnoea, paleness and clamminess.

For peer review only - http://bmjopen.bmj.com/site/about/guidelines.xhtml

From the EMS record, data regarding first measured vital signs was collected. From the hospital and primary care medical records, data on diagnosis on discharge from hospital and previous medical history were retrieved.

During the EMS mission a blood sample was obtained and brought to the ED. This blood sample was analysed in hospital for high-sensitive troponin T using Roche Cobas e 601, detecting values ≥ 5 ng/l. Retrieved high-sensitive troponin T-values were also converted into the following intervals <50, 51-100, 101-1000 and >1000 ng/l. In this way we could stipulate data on troponin T as it would have been presented if Roche's device for bedside troponin T analysis, Cobas h 232, had been used by the EMS.

All registered ECGs were interpreted by either KW or SA using a pre-set ECG interpretation template (Supplemental file 2). Both were blinded to all patient data except age and sex. In cases of uncertainty, interpretation was discussed with senior research cardiologist ML and JH until consensus was reached. Two hundred ECGs were interpreted separately by both KW and SA concerning identification of ST-elevation, ST-depression, right or left bundle branch block. The Kappa coefficient for agreement was 0.810, implying almost perfect agreement.[29] The specific ECG abnormalities included in the Kappa coefficient calculation were chosen based on being most clinically relevant for identification of myocardial ischaemia according to the European Society of Cardiology (ESC).[30 31]

All data except ECG and diagnosis on discharge from hospital was extracted automatically using digital software. Diagnosis on discharge from hospital, according to ICD 10, was collected manually from the medical record by KW. Automatically retrieved data was checked extensively against the original data sources without finding any discrepancies.

Sample size

The planned sample size was 1,500 EMS missions. For a factor present at 10 % of all observations, this would entail that a relative difference of 64 % and an absolute difference of 9 % would be statistically detected. For a factor present at 20 % of all observations, this would entail that a relative difference of 49 % and an absolute difference of 7 % would be detected. For these calculations, 80 % power, significance level of 5 % (two-sided) and a 15 % incidence rate of high-risk conditions are applied. Sample size was later increased to offset the higher rates of missing than predicted in respect of specific variables that were seen early in the inclusion process.

Endpoint

The primary endpoint was a risk-classification group, in terms of low- or high-risk condition. All patients included were classified as having either a low-, intermediate- or high-risk condition as the cause of their chest pain. The adjudication was based on diagnosis on discharge from hospital according to the physician in charge. A high-risk condition was defined as a time-sensitive condition with high mortality rates in need of immediate care from a medical point of view in hindsight, for which transport to hospital with highest priority was called upon. An intermediate-risk condition was defined as a diagnosis probably in need of hospital care, but for which time was not a critical factor. A low-risk condition was defined as a diagnosis with no medical need of hospital treatment, suitable for non-conveyance to hospital. This group also included patients with chest pain who remained at home and did not visit the ED within 72 hours and who did not die within 30 days. The risk-classification of each diagnosis was carried out independently by KW, JH, ML and AB using a pre-set definition for each risk group blinded to patient characteristics. Thereafter, differences that

For peer review only - http://bmjopen.bmj.com/site/about/guidelines.xhtml

had arisen were discussed until consensus was reached. Risk-classification was also carried out by an external cardiologist. When comparing this external risk-classification with that of the article authors, a Kappa coefficient of 0,631, substantial agreement, was reached.[29]

Statistical analysis

All variables were analysed using univariate logistic regression. Each variable was analysed twice, once to test association with high-risk conditions and once to test association with low-risk conditions. If a variable was present in fewer than six patients in any of the groups compared, no analysis was carried out. P-values below 0.05 were considered statistically significant. Variables with a statistical significant association were then included in a forward stepwise logistic regression to retain the final independent predictive variables for high- respectively low-risk conditions. These multivariate analyses where executed on a data set of 657 complete cases (Tnt excluded due to high rates of missing). All analyses were carried out using IBM SPSS Statistics 26.

Ethical considerations

In this study, all patients eligible for inclusion were subject to an opt-out procedure. The study was approved by the Regional Ethical Review Board in Lund (Dnr 2017/212).

RESULTS

In total, 139 patients representing 170 EMS missions declined to participate. These represent 6 % of eligible patients or 5 % of eligible EMS missions. In total 2,917 EMS missions concerning 2,352 unique patients with non-traumatic chest pain were included in the data analysis. These constitute 10 % of all patient-related EMS missions during this time period (inter-hospital transports excluded). Priority 1 (highest priority) was given by the emergency medical dispatcher to 63 % of all missions included, and these constitute 11 % of all priority 1-missions within the region. The EMS nurse then triaged patients on scene using the Rapid Triage and Treatment System (RETTS) [32,33] to the highest priority in 10 % of cases and to the lowest priority in 4 %. EMS missions were equally distributed regarding sex. Median age was 72 years old (Q25-Q75, 58-82) (Supplemental file 3).

In total, 16 % were classified with a high-risk condition, 16 % had an intermediate-risk condition and 68 % had a low-risk condition (Table 1). The all-cause mortality rate within 30 days was 2.9 % (each unique patient counted only once). In the high-risk group mortality rate within 30 days was 8.9 % and in the low-risk group 0.5 %.

For peer review only - http://bmjopen.bmj.com/site/about/guidelines.xhtml

Table 1. Distribution of risk classification and diagnoses by sex and age

	All	Sex		Age group		
	All % (n)	Men % (n)	Women % (n)	≤50 % (n)	51-64 % (n)	≥65 % (n)
	100 (2917)	50.2 (1465)	49.8 (1452)	16.3 (476)	18.5 (539)	65.2 (1902)
High-risk conditions	16.0 (467)	20.3 (298)	11.6 (169)	6.1 (29)	14.8 (80)	18.8 (358)
NSTEMI	6.7 (194)	8.2 (120)	5.1 (74)	2.1 (10)	5.0 (27)	8.3 (157)
STEMI	4.3 (127)	5.9 (87)	2.8 (40)	1.3 (6)	6.5 (35)	4.5 (86)
Unstable angina pectoris	2.1 (60)	2.8 (41)	1.3 (19)	0.4 (2)	1.5 (8)	2.6 (50)
Pulmonary embolism	0.8 (24)	1.0 (14)	0.7 (10)	0.8 (4)	0.7 (4)	0.8 (16)
Undefined AMI	0.4 (11)	0.7 (10)	0.1 (1)	0.0 (0)	0.2 (1)	0.5 (10)
MINCA/MINOCA	0.2 (7)	0.1 (1)	0.4 (6)	0.0 (0)	0.9 (5)	0.1 (2)
Aortic dissection/aneurysm	0.2 (7)	0.4 (6)	0.1 (1)	0.0 (0)	0.0 (0)	0.4 (7)
Severe arrhythmias (ventricular tachycardia. AV-block III etc.)	0.2 (7)	0.3 (5)	0.1 (2)	0.2 (1)	0.0 (0)	0.3 (6)
Takotsubo	0.2 (5)	0.1 (1)	0.3 (4)	0.4 (2)	0.0 (0)	0.2 (3)
Sepsis	0.1 (4)	0.2 (3)	0.1 (1)	0.2 (1)	0.0 (0)	0.2 (3)
Stroke/TIA	0.1 (4)	0.1 (2)	0.1 (2)	0.4 (2)	0.0 (0)	0.1 (2)
Gastric ulcer with perforation/bleeding	0.1 (4)	0.1 (1)	0.2 (3)	0.0 (0)	0.0 (0)	0.2 (4)
Pulmonary oedema	0.1 (2)	0.0 (0)	0.1 (2)	0.0 (0)	0.0 (0)	0.1 (2)
Other, high risk	0.4 (11)	0.5 (7)	4 0.3	0.2 (1)	0.0 (0)	0.5 (10)
Intermediate-risk conditions	15.6 (455)	16.7 (245)	14.5 (210)	8.6 (41)	10.8 (58)	18.7 (356)
Atrial fibrillation/flutter	3.8 (112)	3.9 (57)	3.8 (55)	0.6 (3)	3.0 (16)	4.9 (93)
Heart failure (without pulmonary oedema)	2.1 (60)	2.3 (34)	1.8 (26)	0.0 (0)	0.2 (1)	3.1 (59)
Pneumonia	1.8 (52)	1.7 (25)	1.9 (27)	0.2 (1)	0.7 (4)	2.5 (47)
Myocarditis, pericarditis, endocarditis	1.1 (31)	1.6 (23)	0.6 (8)	2.5 (12)	1.3 (7)	0.6 (12)
Syncope and collapse	0.6 (18)	0.5 (7)	0.8 (11)	0.4 (2)	0.7 (4)	0.6 (12)
Aortic valve stenosis	0.6 (17)	0.7 (10)	0.5 (7)	0.0 (0)	0.2 (1)	0.8 (16)
Tumour	0.6 (17)	0.5 (7)	0.7 (10)	0.2 (1)	0.4 (2)	0.7 (14)
Chronic obstructive pulmonary disease (COPD)	0.5 (14)	0.6 (9)	0.3 (5)	0.0 (0)	0.0 (0)	0.7 (14)
Infection, intermediate risk	0.5 (14)	0.5 (7)	0.5 (7)	0.4 (2)	0.6 (3)	0.5 (9)
Supraventricular tachycardia	0.4 (13)	0.3 (5)	0.1 (2)	0.6 (3)	0.7 (4)	0.3 (6)
Cholelithiasis	0.4 (13)	0.5 (7)	0.4 (6)	0.4 (2)	0.4 (2)	0.5 (9)
Pancreatitis	0.3 (9)	0.3 (5)	0.3 (4)	0.6 (3)	0.2 (1)	0.3 (5)
Electrolyte disturbance	0.2 (7)	0.5 (7)	0.0 (0)	0.2 (1)	0.0 (0)	0.3 (6)
Convulsions and seizures	0.2 (6)	0.3 (4)	0.1 (2)	0.6 (3)	0.4 (2)	0.1 (1)

For peer review only - http://bmjopen.bmj.com/site/about/guidelines.xhtml
For peer review only

Diverticulitis	0.1 (4)	0.3 (4)	0.0 (0)	0.0 (0)	0.0 (0)	0.2 (4)
Other, intermediate risk	2.3 (67)	2.5 (37)	2.1 (30)	1.7 (8)	2.0 (11)	2.5 (48)
Low-risk conditions	68.4 (1995)	62.9 (922)	73.9 (1073)	85.3 (406)	74.4 (401)	62.5 (1188)
Chest pain, unspecified	41.5 (1211)	39.0 (572)	44.0 (639)	52.7 (251)	50.8 (274)	36.1 (686)
Did not convey (No related ED visit in 72 h or death within 30 days)	9.5 (276)	8.1 (118)	0.3 (158)	16.4 (78)	8.7 (47)	7.9 (151)
Angina pectoris (unstable and spasm induced angina excluded)	3.1 (90)	3.3 (48)	2.9 (42)	0.4 (2)	1.5 (8)	4.2 (80)
Abdominal and pelvic pain	2.1 (62)	1.4 (21)	41 (2.8)	2.7 (13)	1.7 (9)	2.1 (40)
Infection, low risk	1.6 (47)	1.3 (19)	1.9 (28)	1.1 (5)	1.9 (10)	1.7 (32)
Gastritis/Gastro-oesophageal reflux disease	1.4 (41)	1.2 (18)	1.6 (23)	2.1 (10)	1.3 (7)	1.3 (24)
Dyspnoea and coughing	1.4 (40)	1.5 (22)	1.2 (18)	1.7 (8)	1.1 (6)	1.4 (26)
Palpitation and benign arrhythmias	1.2 (35)	0.8 (11)	1.7 (24)	1.7 (8)	1.1 (6)	1.1 (21)
Anxiety and other mental disorders	0.9 (25)	0.6 (9)	1.1 (16)	2.7 (13)	0.2 (1)	0.6 (11)
Other pain	0.7 (19)	0.6 (9)	0.7 (10)	0.2 (1)	1.3 (7)	0.6 (11)
Anaemia	0.5 (16)	0.5 (8)	0.6 (8)	0.2 (1)	0.7 (4)	0.6 (11)
Vertigo	0.5 (14)	0.5 (7)	0.5 (7)	0.4 (2)	0.2 (1)	0.6 (11)
Back pain	0.3 (10)	0.2 (3)	0.5 (7)	0.2 (1)	0.2 (1)	0.4 (8)
Orthostatic hypotension	0.3 (10)	0.5 (8)	0.1 (2)	0.0 (0)	0.0 (0)	0.5 (10)
Hypertension	0.3 (10)	0.1 (2)	0.6 (8)	0.2 (1)	0.2 (1)	0.4 (8)
Mental and behavioural disorders due to alcohol	0.3 (10)	0.6 (9)	0.1 (1)	0.4 (2)	0.7 (4)	0.2 (4)
Headache	0.2 (6)	0.1 (1)	0.3 (5)	0.6 (3)	0.2 (1)	0.1 (2)
Other, low risk	2.5 (74)	2.6 (38)	2.5 (36)	1.5 (7)	2.6 (14)	2.8 (53)

The cohort was diagnosed with a wide range of medical conditions involving most of the body's organ systems. Most common was musculoskeletal or unspecified chest pain. This diagnosis was given in 41.5 % of all EMS missions. Twelve percent were given an AMI diagnosis on discharge from hospital. In almost ten percent of the EMS missions, the patient was not conveyed to the ED and did not have a related ED visit within 72 hours or died within 30 days (Table 1).

Patient characteristics and patient presentation differ substantially within the cohort. However, there are some dominating aspects. Patients are old and have a substantial comorbidity – most commonly hypertension or mental disorders. They commonly describe central, constant, pressuring pain about the size of a palm starting several hours earlier during rest. Vital signs are most commonly unaffected. In most cases, the ECG presents alterations known to be associated with underlying pathological conditions. These are the most common findings in patients with both low- and high-risk conditions (Supplemental file 4 and 5).

For peer review only - http://bmjopen.bmj.com/site/about/guidelines.xhtml

For peer review only

Risk prediction

In total, 26 variables showed significantly increased odds and 16 variables showed significantly decreased odds ratio of having a high-risk condition in the univariate analyses (Supplemental file 4). When predicting low-risk conditions, eleven variables showed significantly increased odds ratio for a low-risk condition whereas 37 variables showed a significant increase in the ability to predict the absence of a low-risk condition in the univariate analyses (Supplemental file 5).

When including these variables in a complete cases multivariate analyses 16 and 17 variables remained for high-risk and low-risk prediction respectively. The predictive variables were largely common to both low- and high risk prediction, with old age, ST-elevation and ST-depression the most important predictors. Previous medical history, except psychiatric disorders and atrial fibrillation/flutter, had no predictive value in neither high- or low-risk models. In total high- and low-risk prediction models shared ten predictive variables which are written in italics in Table 2. The area under the receiving operating characteristic curve for prediction of low-risk conditions was 0.806 and 0.833 for high-risk conditions.

For peer review only - http://bmjopen.bmj.com/site/about/guidelines.xhtml

Table 2. Predictors of high- and low-risk conditions after multivariate analysis

	Odds Ratio	CI 95 %	p-value*
**High-risk predictors**			
Age ≤50			<0.001
Age 51-64	7.78	2.096-29.030	0.002
Age ≥65	19.437	5.568-67.850	<0.001
ECG ST-elevation	5.971	2.714-13.137	<0.001
ECG ST-depression	4.229	2.010-8.895	<0.001
ECG Premature Atrial Contractions, PAC	3.841	1.424-10.361	0.008
Pain intensity according to Numeric Rating Scale >8	2.740	1.293-5.805	0.008
Pain in right arm	2.632	1.396-4.965	0.003
Paleness	2.202	1.324-3.663	0.002
Debut during activity	2.155	1.330-3.490	0.002
ECG T wave inversion	1.958	1.056-3.632	0.033
Male	1.808	1.161-2.814	0.009
Pressuring pain	1.559	0.987-2.461	0.057
Central chest pain	1.553	1.000-2.410	0.050
Previous psychiatric diagnosis (any type)	0.596	0.365-0.974	0.039
Time elapsed since pain onset >3 hours	0.505	0.324-0.788	0.003
Previous atrial fibrillation/flutter	0.404	0.237-0.690	0.001
Size of area affected by pain; two inch diameter	0.343	0.136-0.868	0.024
**Low-risk predictors**			
Right sided chest pain	3.410	1.233-9.425	0.018
Previous atrial fibrillation/flutter	2.736	1.622-4.615	<0.001
Previous psychiatric diagnosis (any type)	1.717	1.151-2.561	0.008
ECG QRS duration	0.986	0.978-0.994	0.001
Central chest pain	0.671	0.464-0.971	0.034
Pain intensity according to Numeric Rating Scale >8	0.466	0.230-0.943	0.034
Breathing rate ≥25 breaths/min	0.460	0.248-0.853	0.014
Paleness	0.401	0.252-0.637	<0.001
Pain in right shoulder	0.327	0.139-0.765	0.010
ECG Premature Atrial Contractions, PAC	0.320	0.124-0.827	0.019
ECG Atrial Fibrillation/Flutter	0.297	0.164-0.540	<0.001
Pain in right arm	0.271	0.151-0.487	<0.001
Age ≤50			<0.001
Age 51-64	0.477	0.225-1.010	0.053
Age ≥65	0.261	0.133-0.512	<0.001
Oxygen saturation ≤91 %	0.245	0.092-0.657	0.005
ECG ST-elevation	0.159	0.071-0.356	<0.001
Body temperature >38.0	0.142	0.035-0.580	0.007
ECG ST-depression	0.122	0.052-0.286	<0.001

*Stepwise forward logistic regression, complete cases, entery p < 0.05, removal p < 0.10

Italics = Variable common for both high- and low-risk condition prediction models

For peer review only

DISCUSSION

To our best knowledge this is the first prospective population wide study of unselected prehospital chest pain patients beyond acute coronary syndromes. This is important since the rule out of acute myocardial infarction alone does not allow EMS to alter conveyance. We present models that can identify high- and low-risk underlying conditions with high accuracy and show that most of these patients suffered from a low-risk condition while sixteen percent had an underlying condition with high risk, such as AMI, pulmonary embolism, aortic dissection, arrhythmias, sepsis and bleeding gastric ulcer. This variety of diagnoses stresses the incentive to have broad inclusion criteria in contrast with previous reports.

The present paper aims to inform future risk assessment tools with capabilities to rule in high-risk conditions and rule out low-risk separately. To that end and opposite to expectations classic symptoms of AMI like radiation to left arm or jaws, constant pain, clamminess and dyspnoea did not differ between high- and low-risk conditions in the multivariate analyses. The same is true for previously known risk factors such as history of hypertension, ACS and diabetes. Therefore, future risk models may need to cover other variables, with ability to contribute to accuracy, than traditional risk factors.

One main finding of this study, and maybe also most relevant in clinical care, is that more than two thirds of all EMS missions concerning patients with chest pain were classified as low-risk, i.e. having no need for acute transportation to hospital given the diagnosis on discharge. This implies the substantial magnitude of the impact of a predictive model identifying them with high accuracy early in the care chain. Diverting only a proportion of these low-risk patients away from the ED to less resource intensive venues would probably reduce healthcare costs, EMS workload and ED crowding.

Among chest pain patients, ten percent remained at home, despite EMS personnel having neither mandate nor instruments to provide such recommendations. This finding indicates that even today, EMS personnel or the patients themselves sometimes deem transport to the ED as inappropriate, despite the patients' chest pain. However, little is known about the appropriateness of EMS decision-making when suggesting this type of triage.

Among the sixteen percent of EMS missions with patients with high-risk conditions, ACS constituted the major part. The portion of high-risk conditions is in line with previous findings.[2 4] Importantly STEMI constitute less than one third of all missions concerning high-risk conditions, confirming work by Terkelsen et al.[2] The fairly low incidence of STEMI shows the importance of taking other high-risk diagnoses into account when risk-assessing patients with chest pain. This would for example enable direct transport to CCU, hospitals with PCI or capabilities for thoracic surgery.[10]

Seeking to discriminate between high- and low-risk conditions we also looked for information that would *not* support this aim. In this report we therefore acknowledge that symptoms typically associated with myocardial ischemia, such as central pain, pressuring pain quality, left arm radiation and affected breathing, are common in both groups. Notably most patients with such symptoms were found in the low risk group. This may limit the usefulness of some symptoms previously reported to be of value in risk assessment and triage. However, other aspects of symptoms such as pain intensity, pain in the right arm and the presence of paleness were helpful in the discrimination of high versus low risk.

For peer review only - http://bmjopen.bmj.com/site/about/guidelines.xhtml

Risk assessment in the prehospital setting may serve several purposes. Conveyance decision based on the possibility to identify both low-risk patients suitable for non-hospital care/non-conveyance and high-risk patients in need of prompt specialist care are separate issues. To further inform a future model supporting the conveyance decision a multivariate analysis was reported. The results of the multivariate analyses presented in this study could form the basis for such a decision support tool. The c-statistics indicate that such a tool could reach a level of accuracy appropriate for clinical use. The fact that several classic symptoms and previously known risk factors of AMI turned out to be of little predictive value indicate that previous risk prediction tools developed in the hospital setting such as the HEART-score and RETTS may be less accurate and unvalid when used in the prehospital setting.

The models presented in this study need to be refined before tested further. Adding Tnt to the analyses will probably improve accuracy. However, this requires analyses on imputated data or a new data set with more complete data regarding Tnt. It would also be preferable to develop a combined model predicting both low- and high-risk conditions, given that good c-statistics may be retained. It would also be of interest to examine the accuracy of reduced models with fewer variables. A combined model including fewer variables would ease clinical usage. The result of such a refined model development will be presented in upcoming reports within the research programme. External validation of the final prediction model(s) will be carried out if upcoming results indicate that clinical usage would be appropriate.

Strengths and limitations

A key strength is the nearly complete and unselected inclusion of all EMS missions within the county concerning patients with chest pain during 2018. This increases the generalisability of the results. However, the use of data from only one EMS organisation (i.e. single centre study) negatively impacts the external validity.

Another strength is the complete picture of the study sample as data provides information on demographics, medical history, a wide range of symptoms, vital signs, ECG, biochemical markers and diagnosis on discharge. However, some variables included entail rather high rates of missing information (Supplemental file 4 and 5). When analysing the distribution of missing data, no skewness of clinical relevance could be identified. This is also confirmed by the fact that the results of the univariate analyses to a large extent confirm previous findings concerning which variables were associated with high-risk conditions. This holds true also for variables with the highest rates of missing e.g. pain intensity [34], pain radiation [34], quality of pain [35] and biochemical markers (Tnt).[36]

Data collection in the prehospital setting is known to be challenging. The situation is often perceived as stressful by the patient, the patient may be physically or mentally unable to provide the information requested, personnel resources are limited and protocol compliance among EMS personnel has been reported to be low.[37 38] All these components contribute to the finding that prehospital research often suffers from high rates of missing information. In this study we observed that missing rates were highest regarding data where the questionnaire (Supplemental file 1) required the user to write the answer rather than tick a box. The rates of missing data in these cases can thereby probably mainly be explained by limited protocol compliance among EMS personnel.

The high rate of missing data regarding the biochemical marker (Tnt) has two main reasons. Firstly, no blood sample analysis was done if the patient remained at home. Secondly, a blood sample could not be analysed if the patient was transported directly to a hospital outside the

For peer review only - http://bmjopen.bmj.com/site/about/guidelines.xhtml

county. However these reasons for not obtaining data on Tnt are not a real-life problem if using a device for prehospital bedside troponin T analysis.

Considering that this was a study based on prehospital data, the rates of missing information must be regarded as low and therefore data may be looked upon as comparatively comprehensive. The varying rates of missing information show possible difficulties using certain variables in a prehospital context. For this reason, it is also important to be careful when selecting variables to be included in a planned decision support tool. A tool requiring data on variables which are difficult to collect in the prehospital setting will reduce personnel compliance and thereby be of less clinical value.

CONCLUSIONS

A majority of patients with chest pain cared for by the EMS suffer from a low-risk condition and have no prognostic reason for acute hospital care given their diagnosis on hospital discharge. A smaller proportion has a high-risk condition and is in need of prompt specialist care. Building models with good accuracy for prehospital identification of these groups is possible. Models optimising rule out will differ from models optimising rule in. However, ECG findings and age are cornerstones in both. Importantly previous known risk factors such as history of acute coronary syndrome, diabetes or hypertension did not have a predictive value in such models. On the other hand previous history of psychiatric disorders and atrial fibrillation/flutter are of importance when risk stratifying prehospital patients with chest pain. The use of risk stratification models will make a more personalised care possible with increased patient safety. Research on the value of adding Tnt and how to refine such risk stratification models is needed before clinical testing.

List of abbreviations

ACS - Acute coronary syndrome
AMI - Acute myocardial infarction
CABG - Coronary artery bypass graft
CCU - Cardiac care units
ECG - Electrocardiogram
ED - Emergency department
EMS - Emergency medical services
ESC - European Society of Cardiology
NSTEMI - Non-ST elevation myocardial infarction
PCI - Percutaneous intervention
RETTS - Rapid Emergency Triage and Treatment System
STEMI - ST-elevation myocardial infarction
TNT - Troponin T

Contributors

KW, ML, JH, SA and AB designed the study and planned the data collection. KW, ML, JH and SA interpreted the ECG's. Data analysis was carried out by KW. KW, ML, JH and AB contributed in writing the manuscript. All authors read and approved the final manuscript.

Funding

This study has been funded by the Department of Ambulance and Prehospital Care, Region Halland and the Scientific Council of Region Halland (HALLAND-209901). The funding

For peer review only - http://bmjopen.bmj.com/site/about/guidelines.xhtml

bodies had no role in the design, conduct, interpretation or writing of the report on this research.

Competing interests
The authors declare that they have no competing interests.

Patient consent for publication
Not required.

Ethics approval
The study was approved by the Regional Ethical Review Board in Lund (Dnr 2017/212). All patients were given the opportunity to withdraw their participation using an opt-out procedure.

Data availability statement
The datasets generated and analysed during the current study are not publicly available due the integrity of patient privacy but are available from the corresponding author on reasonable request and if approved by the Regional Ethical Review Board in Lund.

Acknowledgements
We want to thank the following persons:

- The EMS personnel of Region Halland for their help with data collection.
- Thomas Thorsson, Vinh Norberg Nguyen and Sasa Pejicic for data extraction.
- Anders Holmén and Ulf Strömberg for statistical support.

For peer review only

REFERENCES

1. Clawson J, Olola C, Heward A, et al. The Medical Priority Dispatch System's ability to predict cardiac arrest outcomes and high acuity pre-hospital alerts in chest pain patients presenting to 9-9-9. *Resuscitation* 2008;78(3):298-306. doi: 10.1016/j.resuscitation.2008.03.229
2. Pedersen CK, Stengaard C, Friesgaard K, et al. Chest pain in the ambulance; prevalence, causes and outcome - a retrospective cohort study. *Scand J Trauma Resusc Emerg Med* 2019;27(1):84. doi: 10.1186/s13049-019-0659-6 [published Online First: 2019/08/29]
3. Hagiwara MA, Bremer A, Claesson A, et al. The impact of direct admission to a catheterisation lab/CCU in patients with ST-elevation myocardial infarction on the delay to reperfusion and early risk of death: results of a systematic review including meta-analysis. *Scandinavian Journal of Trauma Resuscitation & Emergency Medicine* 2014;22 doi: 10.1186/s13049-014-0067-x
4. Rawshani A, Larsson A, Gelang C, et al. Characteristics and outcome among patients who dial for the EMS due to chest pain. *International Journal of Cardiology* 2014;176(3):859-65. doi: 10.1016/j.ijcard.2014.08.004
5. Ishak M, Ali D, Fokkert MJ, et al. Fast assessment and management of chest pain patients without ST-elevation in the pre-hospital gateway (FamouS Triage): ruling out a myocardial infarction at home with the modified HEART score. *Eur Heart J Acute Cardiovasc Care* 2017:2048872616687116. doi: 10.1177/2048872616687116 [published Online First: 2017/01/01]
6. Thang ND, Karlson BW, Bergman B, et al. Characteristics of and outcome for patients with chest pain in relation to transport by the emergency medical services in a 20-year perspective. *American Journal of Emergency Medicine* 2012;30(9):1788-95. doi: 10.1016/j.ajem.2012.02.014
7. Ravn-Fischer A, Karlsson T, Johanson P, et al. Prehospital ECG signs of acute coronary occlusion are associated with reduced one-year mortality. *Int J Cardiol* 2013;168(4):3594-8. doi: 10.1016/j.ijcard.2013.05.064 [published Online First: 2013/05/30]
8. Beygui F, Castren M, Brunetti ND, et al. Pre-hospital management of patients with chest pain and/or dyspnoea of cardiac origin. A position paper of the Acute Cardiovascular Care Association (ACCA) of the ESC. *Eur Heart J Acute Cardiovasc Care* 2015 doi: 10.1177/2048872615604119 [published Online First: 2015/08/27]
9. Rasmussen MB, Stengaard C, Sørensen JT, et al. Predictive value of routine point-of-care cardiac troponin T measurement for prehospital diagnosis and risk-stratification in patients with suspected acute myocardial infarction. *Eur Heart J Acute Cardiovasc Care* 2017:2048872617745893. doi: 10.1177/2048872617745893 [published Online First: 2017/12/01]
10. Stengaard C, Sørensen JT, Rasmussen MB, et al. Editor's Choice-Acute versus subacute angiography in patients with non-ST-elevation myocardial infarction - the NONSTEMI trial phase I. *Eur Heart J Acute Cardiovasc Care* 2017;6(6):490-99. doi: 10.1177/2048872616648468 [published Online First: 2016/05/06]
11. Carter EJ, Pouch SM, Larson EL. The relationship between emergency department crowding and patient outcomes: a systematic review. *J Nurs Scholarsh* 2014;46(2):106-15. doi: 10.1111/jnu.12055 [published Online First: 2013/12/19]
12. Patton GG, Thakore S. Reducing inappropriate emergency department attendances--a review of ambulance service attendances at a regional teaching hospital in Scotland. *Emergency medicine journal : EMJ* 2013;30(6):459-61. doi: 10.1136/emermed-2012-201116 [published Online First: 2012/07/16]

For peer review only - http://bmjopen.bmj.com/site/about/guidelines.xhtml

13. Pines JM, Pollack CV, Diercks DB, et al. The Association Between Emergency Department Crowding and Adverse Cardiovascular Outcomes in Patients with Chest Pain. *Academic Emergency Medicine* 2009;16(7):617-25. doi: 10.1111/j.1553-2712.2009.00456.x
14. Kulstad EB, Kelley KM. Overcrowding is associated with delays in percutaneous coronary intervention for acute myocardial infarction. *Int J Emerg Med* 2009;2(3):149-54. doi: 10.1007/s12245-009-0107-x [published Online First: 2009/06/05]
15. van Dongen DN, Ottervanger JP, Tolsma R, et al. In-Hospital Healthcare Utilization, Outcomes, and Costs in Pre-Hospital-Adjudicated Low-Risk Chest-Pain Patients. *Appl Health Econ Health Policy* 2019 doi: 10.1007/s40258-019-00502-6 [published Online First: 2019/08/06]
16. Wibring K, Herlitz J, Christensson L, et al. Prehospital factors associated with an acute life-threatening condition in non-traumatic chest pain patients - A systematic review. *Int J Cardiol* 2016;219:373-9. doi: 10.1016/j.ijcard.2016.06.066
17. Lavery T, Greenslade JH, Parsonage WA, et al. Factors influencing choice of pre-hospital transportation of patients with potential acute coronary syndrome: An observational study. *Emerg Med Australas* 2017;29(2):210-16. doi: 10.1111/1742-6723.12735 [published Online First: 2017/01/25]
18. Boothroyd LJ, Lambert LJ, Segal E, et al. Comparison of outcomes of ambulance users and nonusers in ST elevation myocardial infarction. *Am J Cardiol* 2014;114(9):1289-94. doi: 10.1016/j.amjcard.2014.07.060 [published Online First: 2014/09/10]
19. Thuresson M, Jarlöv MB, Lindahl B, et al. Factors that influence the use of ambulance in acute coronary syndrome. *Am Heart J* 2008;156(1):170-6. doi: 10.1016/j.ahj.2008.01.020 [published Online First: 2008/03/14]
20. Rawshani N, Rawshani A, Gelang C, et al. Could ten questions asked by the dispatch center predict the outcome for patients with chest discomfort? *Int J Cardiol* 2016;209:223-5. doi: 10.1016/j.ijcard.2016.02.011 [published Online First: 2016/02/22]
21. van Dongen DN, Tolsma RT, Fokkert MJ, et al. Pre-hospital risk assessment in suspected non-ST-elevation acute coronary syndrome: A prospective observational study. *Eur Heart J Acute Cardiovasc Care* 2018:2048872618813846. doi: 10.1177/2048872618813846 [published Online First: 2018/11/23]
22. Thang ND, Sundstrom BW, Karlsson T, et al. ECG signs of acute myocardial ischemia in the prehospital setting of a suspected acute coronary syndrome and its association with outcomes. *American Journal of Emergency Medicine* 2014;32(6):601-05. doi: 10.1016/j.ajem.2014.03.006
23. Saddichha S, Saxena MK. Is every chest pain a cardiac event? : an audit of patients with chest pain presenting to emergency services in India. *Internal and emergency medicine* 2009;4(3):235-9. doi: 10.1007/s11739-009-0246-3 [published Online First: 2009/04/16]
24. Nehme Z, Boyle MJ, Brown T. Diagnostic accuracy of prehospital clinical prediction models to identify short-term outcomes in patients with acute coronary syndromes: a systematic review. *J Emerg Med* 2013;44(5):946-54.e6. doi: 10.1016/j.jemermed.2012.07.078 [published Online First: 2013/01/13]
25. Wibring K, Herlitz J, Lingman M, et al. Symptom description in patients with chest pain - a qualitative analysis of emergency medical calls involving high-risk conditions. *J Clin Nurs* 2019 doi: 10.1111/jocn.14867 [published Online First: 2019/04/02]
26. Hawker GA, Mian S, Kendzerska T, et al. Measures of adult pain: Visual Analog Scale for Pain (VAS Pain), Numeric Rating Scale for Pain (NRS Pain), McGill Pain

For peer review only - http://bmjopen.bmj.com/site/about/guidelines.xhtml

Questionnaire (MPQ), Short-Form McGill Pain Questionnaire (SF-MPQ), Chronic Pain Grade Scale (CPGS), Short Form-36 Bodily Pain Scale (SF-36 BPS), and Measure of Intermittent and Constant Osteoarthritis Pain (ICOAP). *Arthritis Care Res (Hoboken)* 2011;63 Suppl 11:S240-52. doi: 10.1002/acr.20543
27. Pesonen A, Kauppila T, Tarkkila P, et al. Evaluation of easily applicable pain measurement tools for the assessment of pain in demented patients. *Acta anaesthesiologica Scandinavica* 2009;53(5):657-64. doi: 10.1111/j.1399-6576.2009.01942.x
28. Brunelli C, Zecca E, Martini C, et al. Comparison of numerical and verbal rating scales to measure pain exacerbations in patients with chronic cancer pain. *Health Qual Life Outcomes* 2010;8:42. doi: 10.1186/1477-7525-8-42 [published Online First: 2010/04/22]
29. McHugh ML. Interrater reliability: the kappa statistic. *Biochem Med (Zagreb)* 2012;22(3):276-82.
30. Ibanez B, James S, Agewall S, et al, ESC Guidelines for the management of acute myocardial infarction in patients presenting with ST-segment elevation: The Task Force for the management of acute myocardial infarction in patients presenting with ST-segment elevation of the European Society of Cardiology (ESC). *Eur Heart J* 2018;39(2):119-77. doi: 10.1093/eurheartj/ehx393
31. Roffi M, Patrono C, Collet JP, et al. 2015 ESC Guidelines for the management of acute coronary syndromes in patients presenting without persistent ST-segment elevation: Task Force for the Management of Acute Coronary Syndromes in Patients Presenting without Persistent ST-Segment Elevation of the European Society of Cardiology (ESC). *Eur Heart J* 2016;37(3):267-315. doi: 10.1093/eurheartj/ehv320 [published Online First: 2015/08/29]
32. Widgren BR, Jourak M. Medical Emergency Triage and Treatment System (METTS): a new protocol in primary triage and secondary priority decision in emergency medicine. *J Emerg Med* 2011;40(6):623-8. doi: 10.1016/j.jemermed.2008.04.003 [published Online First: 2008/10/18]
33. Magnusson C, Herlitz J, Axelsson C. Patient characteristics, triage utilisation, level of care, and outcomes in an unselected adult patient population seen by the emergency medical services: a prospective observational study. *BMC Emerg Med* 2020;20(1):7. doi: 10.1186/s12873-020-0302-x [published Online First: 2020/01/30]
34. Reuter PG, Pradeau C, Huo Yung Kai S, et al. Predicting acute coronary syndrome in males and females with chest pain who call an emergency medical communication centre. *Scand J Trauma Resusc Emerg Med* 2019;27(1):92. doi: 10.1186/s13049-019-0670-y [published Online First: 2019/10/17]
35. Stopyra JP, Harper WS, Higgins TJ, et al. Prehospital Modified HEART Score Predictive of 30-Day Adverse Cardiac Events. *Prehosp Disaster Med* 2018;33(1):58-62. doi: 10.1017/S1049023X17007154 [published Online First: 2018/01/10]
36. van Dongen DN, Fokkert MJ, Tolsma RT, et al. Value of Prehospital Troponin Assessment in Suspected Non-ST-Elevation Acute Coronary Syndrome. *Am J Cardiol* 2018 doi: 10.1016/j.amjcard.2018.07.037 [published Online First: 2018/08/20]
37. Maurin Söderholm H, Andersson H, Andersson Hagiwara M, et al. Research challenges in prehospital care: the need for a simulation-based prehospital research laboratory. *Adv Simul (Lond)* 2019;4:3. doi: 10.1186/s41077-019-0090-0 [published Online First: 2019/02/13]

For peer review only - http://bmjopen.bmj.com/site/about/guidelines.xhtml

38. Dickert NW, Brown J, Cairns CB, et al. Confronting Ethical and Regulatory Challenges of Emergency Care Research With Conscious Patients. *Ann Emerg Med* 2016;67(4):538-45. doi: 10.1016/j.annemergmed.2015.10.026 [published Online First: 2015/12/18]

For peer review only

For peer review only - http://bmjopen.bmj.com/site/about/guidelines.xhtml

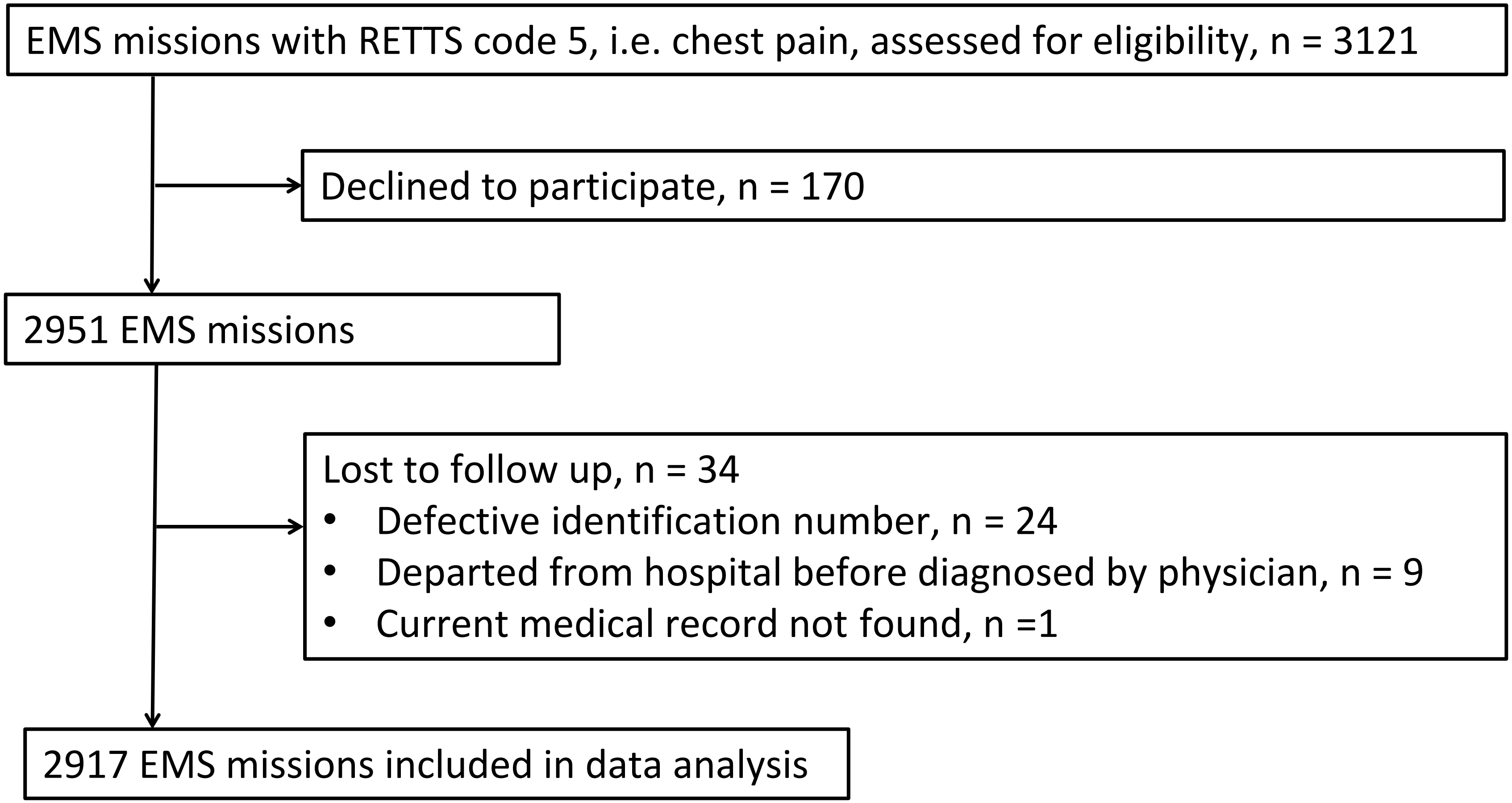
EMS missions with RETTS code 5, i.e. chest pain, assessed for eligibility, n = 3121
Declined to participate, n = 170
2951 EMS missions
Lost to follow up, n = 34
• Defective identification number, n = 24
• Departed from hospital before diagnosed by physician, n = 9
• Current medical record not found, n =1
2917 EMS missions included in data analysis

Supplemental material 2 - ECG interpretation manual

1. Heart rate: Provided by computer.
2. Sinus rhythm: Positive P wave in lead II with a constant morphology, rate between 50 and 100/min, constant PQ interval and normal QRS morphology.(2)
3. Sinus Bradycardia: Positive P-wave in lead II with a constant morphology, rate <50/min, constant PQ interval and normal QRS morphology.(2)
4. Sinus Tachycardia: Positive P wave in lead II with a constant morphology, rate >100/min, constant PQ interval and normal QRS morphology.(2)
5. Supraventricular Tachycardia: Fast and regular ventricular rhythm usually between 150-200/min. Episodic presentation with sudden onset. Normal QRS-complexes.(2)
6. Atrial Fibrillation: No evident P-waves instead the ECG reveals low-amplitude baseline oscillations (fibrillary or f waves from the fibrillating atria) and an irregular ventricular rhythm. Normal QRS complex duration during AV conduction.(2, 5)
7. Atrial Flutter: In typical atrial flutter, the ECG reveals identically recurring, regular, sawtooth flutter waves, and evidence of continual electrical activity (lack of an isoelectric interval between flutter waves), often best visualized in leads II, III, aVF, or V1. The atrial rate during typical atrial flutter is usually 250 to 350 beats/min with variations in the degree of ventricular conduction, usually 2:1 conduction. Normal QRS complex duration during AV conduction.(2, 5)
8. Atrial pacing: Pacing spike precedes the p wave. Morphology of p wave dependent of lead placement but may appear normal.
9. Ventricular pacing: Pacing spike precedes the QRS complex.
10. Second Degree AV block – II type 1: Progressive prolongation of the PR interval culminating in a non-conducted P wave. The PR interval is longest immediately before the dropped beat. The PR interval is shortest immediately after the dropped beat.(5)
11. Second Degree AV block – II type 2: Intermittent non-conducted P waves without progressive prolongation of the PR interval. The PR interval in the conducted beats remains constant. The P waves continue at a constant rate. The RR interval surrounding the dropped beat(s) is an exact multiple of the preceding RR interval.(5)
12. Third Degree AV block – III: Normal P waves with regular rhythm. Complete absence of AV conduction – none of the supraventricular impulses are conducted to the ventricles. Perfusing rhythm is maintained by a junctional or ventricular escape rhythm.(5)
13. Suspected STEMI: ST-elevation of the J-point in two contiguous leads with ≥ 2.5 mm in men < 40 years, ≥ 2 mm in men > 40 years, or ≥ 1,5 mm in women in V2-V3 and/or ≥ 1 mm in the other leads.(1)
14. ST-Depression: ST-depression ≥ 0.5 mm in at least two contiguous leads. (3)
15. T-wave inversion: Negative electrical axis of the T wave. Positive T wave is always expected at least in the precordial leads V2-V6 and in AVL where the R wave is at least 5mm, deviation from this rule is pathologic.
16. Q-wave: Pathologic q-wave ≥ 25% of R-wave or duration of ≥0,03 s in two contiguous leads. Q-wave or QS-komplex in V2 or V3 is always pathologic. (2)
17. QRS-duration: Provided by computer.

For peer review only

18. Premature Ventricular Complex-PVC: Characterized by the premature occurrence of a QRS complex that is abnormal in shape and has a duration usually exceeding the dominant QRS complex, generally longer than 120 milliseconds. The T wave is usually large and opposite in direction to the major deflection of the QRS. The QRS complex is not preceded by a premature P wave but can be preceded by a nonconducted sinus P wave occurring at its expected time.(5)
19. PAC-SVES: Premature Atrial Complexes: Premature beat with or without a premature P wave that generally differs from a normal sinus P wave. Normal QRS complex. (2,5)
20. Left Bundle Branch Block – LBBB: QRS duration of ≥ 120 ms. Dominant S wave in V1-V3. Broad M-shaped QRS complexes in lateral leads (V5-V6), ST and T waves usually opposite in direction to QRS. Absence of Q waves in lateral leads (I, V5-V6; small Q waves are still allowed in aVL). Prolonged R wave peak time > 60ms in left precordial leads (V5-6).(5,6)
21. Right Bundle Branch Block – RBBB: QRS duration ≥ 120 msec. rsr′, rsR′, or rSR′, patterns in leads V1 and V2. S waves in leads I and V6 ≥ 40 msec wide. Normal time to peak R wave in leads V5 and V6 but > 50 msec in V1.(5)
22. Ventricular Tachycardia: VT is suggested by the occurrence of a series of three or more consecutive, abnormally shaped QRS complexes longer than 120 milliseconds, with the ST-T vector pointing opposite the major QRS deflection. The R-R interval can be regular or varying. Atrial activity can be independent of ventricular activity (AV dissociation), or the atria can be depolarized retrogradely (ventriculoatrial [VA] association). Depending on the particular type of VT, rates range from 70 to 250 beats/min, usually > 100 beats/min.(2,5)
23. Uninterpretable ECG: Meaningful ECG interpretation not possible due to low ECG quality as judged by interpreter.

References

1. Ibanez B, James S, Agewall S, Antunes MJ, Bucciarelli-Ducci C, Bueno H, et al. 2017 ESC Guidelines for the management of acute myocardial infarction in patients presenting with ST-segment elevationThe Task Force for the management of acute myocardial infarction in patients presenting with ST-segment elevation of the European Society. Eur Heart J. 2018 Jan 7;39(2):119–77.
2. Jern Sverker. Klinisk EKG-Diagnostik. 2010;
3. Roffi M, Patrono C, Collet J-P, Mueller C, Valgimigli M, Andreotti F, et al. 2015 ESC Guidelines for the management of acute coronary syndromes in patients presenting without persistent ST-segment elevationTask Force for the Management of Acute Coronary Syndromes in Patients Presenting without Persistent ST-Segment Elevation of th. Eur Heart J. 2016 Jan 14;37(3):267–315.
4. Rhinehardt J, Brady WJ, Perron AD, Mattu A. Electrocardiographic manifestations of Wellens' syndrome. Am J Emerg Med. 2002 Nov 1;20(7):638–43.
5. Douglas P. Zipes, Peter Libby, Robert O. Bonow, Douglas L. Mann, Gordon F. Tomaselli and EB. Braunwald's Heart Disease: A Textbook of Cardiovascular Medicine 11th edition. 2019;
6. Surawicz B, Childers R, Deal BJ, Gettes LS. AHA/ACCF/HRS Recommendations for the

For peer review only

Standardization and Interpretation of the Electrocardiogram. Part III: Intraventricular Conduction Disturbances A Scientific Statement From the American Heart Association Electrocardiography and Arrhythmias Committee, . J Am Coll Cardiol. 2009 Mar 17;53(11):976–81.

For peer review only

Supplemental material 1 - Analog version of study questionnaire

1. Patient is (several options possible)?
□Pale □Clammy □Neither □ Can't tell

2. Patient is nauseas, is vomiting or has been vomiting (several options possible)?
□Nausea □Vomiting/has been vomiting □Neither □ Can't tell

3. The patient experiences his/her breathing as affected?
□Affected □Unaffected □ Can't tell

4. Pain intensity (when resting) on EMS arrival according to NRS (0-10, 0 = "no pain", 10 = "worst imaginable pain").
NRS: 0 1 2 3 4 5 6 7 8 9 10

If NRS is not possible to use, ask the patient to rate his/her pain as follows:
□No pain □Mild pain □Moderate pain □Severe pain □Unbearable pain

5. At what time and date did the pain, inducing the EMS contact, start?
Time: □ Can't tell
Date:

6. How did the pain start?
□Suddenly (seconds) □Quickly (minutes) □Slowly (hours) □ Can't tell

7. Did the pain start in conjunction with (several options possible)?
□Activity □While resting □In pain when waking up □ Can't tell

8. Development of pain (several options possible).
□Pain is constant □Pain come and go □Pain increases gradually □ Can't tell

9. How does the patient describe his/her pain (several options possible)?
Do the patient describe his/her pain as pressuring, cramping, burning, cutting, stabbing, dull, discomfort, swaying, bursting etc. Please write below.

10. Chest pain localisation (several options possible)?
□Central □Left side of chest □Right side of chest □Upper half of chest
□Lower half of chest □Entire chest □ Can't tell

11. Do the patient have pain somewhere else other than in their chest (several different areas/body parts can be specified).
Please write below which area/body part that hurts or if the chest pain is radiating somewhere. If no other pain than chest pain, write: "No other pain".

12. Size of area affected by pain?
□Size of a 5 sek coin □Size of a palm □Entire chest □ Can't tell

13. Chest pain affected by palpation?
□Yes □No □ Can't tell

14. Is pain affected by movement?
□Yes □No □ Can't tell

15. Is pain affected by breathing?
□Yes □No □ Can't tell

Supplemental material 3 - Description of study sample

	All n (%)	Sex	
		Men n (%)	Women n (%)
	2917 (100)	1465 (50.2)	1452 (49.8)
Mean age (SD)	69 (17)	67 (18)	70 (17)
Median age (Q1-Q3)	72 (58-82)	70 (56-80)	73 (60-83)
Priority by EMD[a]			
Priority 1	1836 (62.9)	936 (63.9)	900 (62.0)
Priority 2	1049 (36.0)	513 (35)	536 (36.9)
Priority 3	32 (1.1)	16 (1.1)	16 (1.1)
Priority 4	0 (0)	0 (0.0)	0 (0.0)
Priority by EMS-personnel[b]			
Priority 1	311 (10.7)	208 (14.2)	103 (7.1)
Priority 2	1969 (67.5)	981 (67.0)	988 (68)
Priority 3	325 (11.1)	139 (9.5)	186 (12.8)
Priority 4	10 (0.3)	4 (0.3)	6 (0.4)
Did not convey	302 (10.4)	133 (9.1)	169 (11.6)
Priority according to RETTS[c] by EMS-personnel[b]			
Red	290 (9.9)	187 (12.8)	103 (7.1)
Orange	1628 (55.8)	819 (55.9)	809 (55.7)
Yellow	887 (30.4)	419 (28.6)	468 (32.2)
Green	112 (3.8)	40 (2.7)	72 (5.0)
Transport from primary care centre	508 (17.4)	286 (19.5)	222 (15.3)
Transport to primary care centre	15 (0.5)	8 (0.5)	7 (0.5)
Transported to hospital	2600 (89.1)	1324 (90.4)	1276 (87.9)
Admitted to hospital (missing = 1)	1409 (48.3)	812 (55.4)	597 (41.1)
Did not convey	302 (10.4)	133 (9.1)	169 (11.6)
EMS response time (minutes)			
Median EMS dispatch to scene arrival (Q1-Q3)	10 (6-15)	9 (6-15)	10 (6-16)
Median EMS time at scene (Q1-Q3)	25 (18-31)	24 (18-30)	25 (19-32)
Median transportation time (Q1-Q3)	25 (13-34)	25 (14-33)	25 (12-35)
Median total EMS time (Q1-Q3)	95 (73-118)	95 (73-118)	95 (73-118)

[a]Emergency dispatch centre

[b]Emergency medical services

[c]Rapid Emergency Triage and Treatment System

For peer review only

Age group		
≤50 n (%)	51-64 n (%)	≥65 n (%)
476 (16.3)	539 (18.5)	1902 (65.2)
39 (9)	58 (4)	79 (8)
41 (31-47)	58 (55-62)	79 (73-85)
280 (55.5)	232 (59.9)	1233 (64.8)
189 (39.7)	208 (38.6)	652 (34.3)
7 (1.5)	8 (1.5)	17 (0.9)
0 (0.0)	0 (0.0)	0 (0.0)
37 (7.8)	68 (12.3)	206 (10.8)
286 (60.1)	355 (65.9)	1328 (69.8)
68 (14.3)	63 (11.7)	194 (10.2)
0 (0.0)	2 (0.4)	8 (0.4)
85 (17.9)	51 (9.5)	166 (8.7)
32 (6.7)	59 (10.9)	199 (10.5)
239 (50.2)	291 (54.0)	1098 (57.7)
170 (35.7)	170 (31.5)	547 (28.8)
35 (7.4)	19 (3.5)	58 (3.0)
77 (16.2)	146 (27.1)	285 (15.0)
3 (0.6)	0 (0.0)	12 (0.6)
388 (81.5)	488 (90.5)	1724 (90.6)
123 (25.8)	235 (43.6)	1051 (55.3)
85 (17.9)	51 (9.5)	166 (8.7)
10 (7-16)	10 (6-17)	9 (6-15)
22 (16-27)	22 (15-27)	26 20-33)
25 (13-35)	24 (13-32)	25 13-34)
89 (67-115)	92 (70-113)	97 75-120)

For peer review only

Supplemental material 4 - Prediction of high-risk conditions, univariate analyses

		Total with variable % (n)	Patients with high-risk condition % (n)
All		2917 (100)	16.0 (467)
	Men	50.2 (1465)	63.8 (298)
	Women	49.8 (1452)	36.2 (169)
Age		72 (median)	75 (median)
Age-group			
	Age ≤50	16.3 (476)	6.2 (29)
	Age 51-64	18.5 (539)	17.1 (80)
	Age ≥65	65.2 (1902)	76.7 (358)
**Previous history of the following according to medical record**			
Acute coronary syndrome (AMI, unstable angina)		29.3 (856)	27.8 (130)
Chronic obstructive pulmonary disease		10.5 (307)	7.5 (35)
Angina pectoris		20.0 (582)	16.9 (79)
Hypertension		58.1 (1696)	63.2 (295)
Heart failure		20.6 (600)	18.0 (84)
Diabetes mellitus (type I and II)		19.8 (578)	23.6 (110)
Stroke		8.2 (240)	7.9 (37)
Kidney disease		12.0 (350)	12.6 (59)
Atrial fibrillation/flutter		26.3 (767)	15.8 (74)
Rheumatism		2.3 (66)	1.5 (7)
Cancer (any type)		17.2 (502)	18.6 (87)
Psychiatric diagnosis (any type)		38.9 (1136)	27.4 (128)
**Vital signs on EMS arrival (missing)**			
Breathing rate ≤8 breaths/min (22)		0 (0)	0 (0)
Breathing rate ≥25 breaths/min (22)		10.2 (297)	14.7 (68)
Oxygen saturation ≤91 % (8)		3.8 (110)	6.4 (30)
Heart rate ≤40 beats/min (18)		0.2 (7)	0.9 (4)
Heart rate ≥131 beats/min (18)		3.1 (91)	2.4 (11)
Systolic blood pressure ≤90 mmHg (22)		1.0 (29)	1.5 (7)
Systolic blood pressure ≥220 mmHg (22)		1.0 (28)	0.4 (2)
Decreased level of consciousness or new confusion (68)		0.9 (25)	1.5 (7)
Body temperature ≤35.0 (110)		0.2 (6)	0.5 (2)
Body temperature >38.0 (110)		2.3 (67)	2.0 (9)
**Symptoms on EMS arrival (missing)**			
Pale (565)		16.4 (386)	29.1 (111)
Clammy (565)		8.7 (204)	14.4 (55)
Nausea (576)		27.1 (635)	26.8 (101)
Vomiting (576)		7.0 (165)	7.4 (28)
Affected breathing according to patient (596)		44.6 (1040)	41.9 (158)
Pain intensity according to Numeric Rating Scale. NRS 0-10 (415)			
	0	20.3 (509)	17.4 (71)

For peer review only

1	3.8 (94)	3.2 (13)
2	11.5 (287)	9.8 (40)
3	7.6 (189)	8.6 (35)
4	8.1 (202)	6.8 (28)
5	16.7 (418)	15.4 (63)
6	8.3 (207)	6.8 (28)
7	10.5 (263)	12.2 (50)
8	8.2 (206)	9.8 (40)
9	2.6 (64)	4.9 (20)
10	2.5 (63)	5.1 (21)
Time elapsed since pain onset >3 hours (1007)	45.2 (863)	40.3 (128)
Debut		
Debut during activity (752)	22.1 (479)	32.8 (116)
Debut while resting (752)	65.5 (1419)	59.6 (211)
Debut while sleeping (752)	15.8 (342)	11.3 (40)
Sudden debut, within seconds (875)	35.7 (729)	33.0 (115)
Quick debut, within minutes (875)	35.2 (718)	42.7 (149)
Slow debut, within hours (875)	29.1 (595)	24.4 (85)
Constant pain (732)	55.5 (1212)	61.8 (235)
Fluctuating pain (732)	40.4 (883)	30.5 (116)
Pain aggravating over time (732)	10.8 (237)	8.7 (33)
Pain in other parts of the body (1197)		
Head	2.5 (43)	0.7 (2)
Throat	10.3 (177)	8.2 (24)
Jaw	5.3 (92)	7.5 (22)
Neck	2.5 (43)	2.7 (8)
Between scapulars	2.2 (37)	4.1 (12)
Back	15.2 (261)	15.4 (45)
Left shoulder	8.5 (147)	7.9 (23)
Right shoulder	4.2 (72)	6.2 (18)
Left arm	24.0 (412)	33.6 (98)
Right arm	8.4 (145)	16.4 (48)
Left hand	1.0 (17)	0.7 (2)
Right hand	0.3 (6)	0.3 (1)
Stomach	7.0 (121)	4.8 (14)
Left leg	1.7 (29)	0.7 (2)
Right leg	1.4 (24)	0.0 (0)
No other pain	39.3 (676)	36.3 (106)
Pain quality (1175)		
Band-shaped	3.3 (58)	5.2 (15)
Burning	4.4 (76)	4.5 (13)
Stabbing	9.7 (169)	4.2 (12)
Cramping	8.7 (151)	9.8 (28)
Dull pain	13.9 (242)	13.3 (38)
Fells like something is on the chest	0.7 (12)	1.0 (3)

For peer review only

Discomfort	10.2 (178)	8.7 (25)
Tingling/Stinging	5.7 (99)	1.4(4)
Swaying	1.7 (30)	1.4 (4)
Pressuring	57.9 (1008)	63.6 (182)
Heaviness	1.0 (17)	1.4 (4)
Aching	2.4 (41)	3.1 (9)
Chest pain localization (640)		
Central pain	53.4 (1215)	64.5 (240)
Left side of chest	35.5 (809)	28.0 (104)
Right side of chest	5.1 (116)	2.4 (9)
Upper part of chest	6.7 (152)	7.3 (27)
Lower part of chest	9.0 (204)	5.9 (22)
All over the chest	11.8 (269)	12.6 (47)
Size of area affected by pain (794)		
Two inch diameter	10.7 (228)	5.1 (18)
Size of patient's palm	58.4 (1240)	61.0 (216)
Entire chest	30.9 (655)	33.9 (120)
Palpation tenderness (655)	22.3 (505)	15.8 (58)
Pain affected by movement (719)	17.0 (373)	11.5 (41)
Pain affected by breathing (692)	25.8 (573)	17.3 (63)
Troponin T (missing)		
high-sensitive Troponin T (1416)	12 (median)	33 (median)
Troponin T with analyse levels adjusted to Roche Cobas h232 (1416)		
Tnt <50 ng/L	88.1 (1323)	62.9 (161)
Tnt 51-100 ng/L	6.6 (99)	15.6 (40)
Tnt 101-1000 ng/L	4.5 (67)	17.2 (44)
Tnt >1000 ng/L	0.8 (12)	4.3 (11)
ECG (missing[b])		
ECG transmitted to hospital	95.5 (2785)	97.2 (454)
Sinus Rhythm, SR (235)	83.8 (2248)	84.7 (377)
Sinus Bradycardia (235)	1.3 (34)	1.1 (5)
Sinus Tachycardia (235)	8.9 (240)	12.4 (55)
Supraventricular Tachycardia, SVT (235)	0.5 (14)	0.2 (1)
Atrial Fibrillation/Flutter, AF (235)	14.3 (383)	13.9 (62)
Ventricular Tachycardia, VT (235)	0.0 (1)	0.0 (0)
Atrial Pacing (235)	1.0 (29)	0.4 (2)
Ventricular Pacing (150)	3.1 (85)	1.8 (8)
AV-block II type 1 (235)	0.0 (1)	0.2 (1)
AV-block II type 2 (235)	0.0 (1)	0.0 (0)
AV-Block III (235)	0.1 (2)	0.4 (2)
ST-Elevation (235)	6.1 (164)	22.9 (102)
ST-Depression (235)	7.6 (204)	23.8 (106)
T-wave Inversion (235)	14.5 (388)	22.7 (101)
Q-wave (235)	4.8 (130)	6.5 (29)

For peer review only

Premature Ventricular Contraction, PVC (235)	5.5 (147)	7.9 (35)
Premature Atrial Contractions, PAC (235)	3.5 (94)	6.1 (27)
Left Bundle Branch Block, LBBB (235)	6.3 (168)	6.5 (29)
Right Bundle Branch Block, RBBB (235)	6.3 (170)	9.2 (41)
Sinus rhythm and none of abnormalities above (150)	44.6 (1233)	26.5 (120)
QRS-duration (150)	92 (median)	96 (median)
Uninterpretable ECG (132)	0.6 (18)	0.2 (1)

*p < 0,05
**p < 0,01
***p < 0,001
[a]Logistic regression
[b]When ventricular pacing or uninterpretable remaining ECG interpretation has been treated as missing.

For peer review only

Patients without high-risk condition % (n)	High-risk vs without high-risk, p-value[a]	Odds Ratio	CI 95 %
84.0 (2450)			
47.6 (1167)	<0.001***	1.939	1.580-2.379
52.4 (1283)			
71 (median)	<0.001***	1.020	1.014-1.027
18.2 (447)			
18.7 (459)	<0.001***	2.686	1.723-4.190
63.0 (1544)	<0.001***	3.574	2.413-5.293
29.6 (726)	0.435	0.916	0.735-1.142
11.2 (272)	0.021*	0.649	0.450-0.936
20.5 (503)	0.074	0.788	0.607-1.023
57.2 (1401)	0.016*	1.284	1.047-1.575
21.1 (516)	0.132	0.822	0.637-1.061
19.1 (468)	0.027*	1.305	1.030-1.653
8.3 (203)	0.794	0.952	0.661-1.372
11.9 (291)	0.645	1.073	0.795-1.447
28.3 (693)	<0.001***	0.477	0.367-0.621
2.4 (59)	0.230	0.617	0.280-1.359
16.9 (415)	0.375	1.123	0.869-1.450
41.1 (1008)	<0.001***	0.540	0.434-0.672
0 (0)	–	–	–
9.4 (229)	0.001**	1.656	1.238-2.216
3.3 (80)	0.001**	2.027	1.316-3.122
0.1 (3)	–	–	–
3.3 (80)	0.295	0.711	0.376-1.346
0.9 (22)	0.230	1.690	0.718-3.980
1.1 (26)	–	–	–
0.7 (18)	0.114	2.030	0.843-4.888
0.2 (4)	–	–	–
2.5 (58)	0.599	0.827	0.407-1.681
14.0 (275)	<0.001***	2.525	1.956-3.258
7.6 (149)	<0.001***	2.056	1.477-2.862
27.2 (534)	0.873	0.980	0.764-1.257
7.0 (137)	0.754	1.070	0.701-1.632
45.1 (882)	0.248	0.877	0.702-1.096
20.9 (438)	<0.001***		

For peer review only

3.9 (81)	0.976	0.990	0.524-1.872
11.8 (247)	0.996	0.999	0.658-1.517
7.4 (154)	0.136	1.402	0.899-2.187
8.3 (174)	0.976	0.993	0.620-1.590
17.0 (355)	0.629	1.095	0.758-1.580
8.6 (179)	0.882	0.965	0.603-1.545
10.2 (213)	0.068	1.448	0.973-2.154
7.9 (166)	0.069	1.487	0.970-2.278
2.1 (44)	0.001**	2.804	1.562-5.033
2.4 (42)	<0.001***	3.085	1.726-5.513
46.2 (735)	0.053	0.786	0.615-1.003
20.0 (363)	<0.001***	1.944	1.514-2.496
66.7 (1208)	0.010*	0.737	0.583-0.930
16.7 (302)	0.012*	0.637	0.448-0.905
36.3 (614)	0.239	0.864	0.677-1.103
33.6 (569)	0.001**	1.472	1.164-1.861
30.1 (510)	0.031*	0.747	0.573-0.974
49.9 (977)	<0.001***	1.662	1.312-2.106
39.2 (767)	0.001**	0.662	0.521-0.842
10.4 (204)	0.279	0.808	0.549-1.189
2.9 (41)	–	–	–
10.7 (153)	0.202	0.746	0.476-1.170
4.9 (70)	0.071	1.581	0.962-2.597
2.5 (35)	0.773	1.121	0.515-2.442
1.8 (25)	0.014*	2.405	1.194-4.844
15.1 (216)	0.902	1.022	0.721-1.449
8.7 (124)	0.653	0.899	0.565-1.430
3.8 (54)	0.067	1.672	0.965-2.894
22.0 (314)	<0.001***	1.792	1.364-2.355
6.8 (97)	<0.001***	2.699	1.861-3.915
1.1 (15)	–	–	–
0.4 (5)	–	–	–
7.5 (107)	0.103	0.622	0.351-1.101
1.9 (27)	–	–	–
1.7 (24)	–	–	–
39.9 (570)	0.249	0.858	0.661-1.114
3.0 (43)	0.051	1.819	0.996-3.321
4.3 (63)	0.869	1.053	0.572-1.940
10.8 (157)	0.001**	0.362	0.199-0.661
8.4 (123)	0.461	1.176	0.764-1.811
14.0 (204)	0.746	0.940	0.648-1.364
0.6 (9)	–	–	–

For peer review only

10.5 (153)	0.368	0.816	0.524-1.271
6.5 (95)	–	–	–
1.8 (26)	–	–	–
56.7 (826)	0.031*	1.335	1.027-1.735
0.9 (13)	–	–	–
2.2 (32)	0.336	1.446	0.683-3.063
51.2 (975)	<0.001***	1.734	1.377-2.184
37.0 (705)	0.001**	0.661	0.517-0.844
5.6 (107)	0.013*	0.417	0.209-0.830
6.6 (125)	0.623	1.114	0.724-1.716
9.6 (182)	0.026*	0.595	0.377-0.940
11.7 (222)	0.592	1.096	0.783-.1.535
11.9 (210)	<0.001***	0.398	0.242-0.653
57.9 (1024)	0.275	1.139	0.902-1.438
30.2 (535)	0.174	1.183	0.928-1.507
23.6 (447)	0.001**	0.608	0.451-0.821
18.0 (332)	0.003**	0.597	0.416-0.831
27.4 (510)	<0.001***	0.552	0.413-0.738
11 (median)	<0.001***	1.013	1.010-1.016
93.3 (1162)	<0.001***		
4.7 (59)	<0.001***	4.893	3.170-7.552
1.8 (23)	<0.001***	13.807	8.122-23.471
0.1 (1)	–	–	–
95.1 (2331)	0.051	1.783	0.997-3.188
83.6 (1871)	0.572	1.085	0.818-1.437
1.3 (29)	0.766	0.865	0.333-2.247
8.3 (185)	0.006	1.564	1.136-2.154
0.6 (3)	–	–	–
14.3 (321)	0.818	0.966	0.721-1.295
0.2 (1)	–	–	–
1.2 (27)	–	–	–
3.3 (77)	0.083	0.522	0.250-1.089
0.0 (0)	–	–	–
0.0 (1)	–	–	–
0.0 (0)	–	–	–
2.8 (62)	<0.001***	10.432	7.458-14.591
4.4 (98)	<0.001***	5.825	5.068-9.190
12.8 (287)	<0.001***	1.995	1.547-2.572
4.5 (101)	0.074	1.474	0.963-2.257

For peer review only

5.0 (112)	0.016*	1.620	1.092-2.402
3.0 (67)	0.002**	2.092	1.322-3.310
6.2 (139)	0.810	1.052	0.696-1.591
5.8 (129)	0.007**	1.658	1.149-2.394
48.1 (1113)	<0.001***	0.389	0.311-0.487
92 (median)	<0.001***	1.009	1.005-.1.013
0.7 (17)	–	–	–

.

For peer review only

Supplemental material 5 - Prediction of low-risk conditions, univariate analyses

		Total with variable % (n)
All		2917 (100)
	Men	50.2 (1465)
	Women	49.8 (1452)
Age		72 (median)
Age-group		
	Age ≤50	16.3 (476)
	Age 51-64	18.5 (539)
	Age ≥65	65.2 (1902)
Previous history of the following according to medical record		
Acute coronary syndrome (AMI, unstable angina)		29.3 (856)
Chronic obstructive pulmonary disease		10.5 (307)
Angina pectoris		20.0 (582)
Hypertension		58.1 (1696)
Heart failure		20.6 (600)
Diabetes mellitus (type I and II)		19.8 (578)
Stroke		8.2 (240)
Kidney disease		12.0 (350)
Atrial fibrillation/flutter		26.3 (767)
Rheumatism		2.3 (66)
Cancer (any type)		17.2 (502)
Psychiatric diagnosis (any type)		38.9 (1136)
Vital signs on EMS arrival (missing)		
Breathing rate ≤8 breaths/min (22)		0 (0)
Breathing rate ≥25 breaths/min (22)		10.2 (297)
Oxygen saturation ≤91 % (8)		3.8 (110)
Heart rate ≤40 beats/min (18)		0.2 (7)
Heart rate ≥131 beats/min (18)		3.1 (91)
Systolic blood pressure ≤90 mmHg (22)		1.0 (29)
Systolic blood pressure ≥220 mmHg (22)		1.0 (28)
Decreased level of consciousness or new confusion (68)		0.9 (25)
Body temperature ≤35.0 (110)		0.2 (6)
Body temperature >38.0 (110)		2.3 (67)
Symptoms on EMS arrival (missing)		
Pale (565)		16.4 (386)
Clammy (565)		8.7 (204)
Nausea (576)		27.1 (635)
Vomiting (576)		7.0 (165)
Affected breathing according to patient (596)		44.6 (1040)
Pain intensity according to Numeric Rating Scale. NRS 0-10 (415)		
	0	20.3 (509)

For peer review only

	1	3.8 (94)
	2	11.5 (287)
	3	7.6 (189)
	4	8.1 (202)
	5	16.7 (418)
	6	8.3 (207)
	7	10.5 (263)
	8	8.2 (206)
	9	2.6 (64)
	10	2.5 (63)
Time elapsed since pain onset >3 hours (1007)		45.2 (863)
Debut		
	Debut during activity (752)	22.1 (479)
	Debut while resting (752)	65.5 (1419)
	Debut while sleeping (752)	15.8 (342)
	Sudden debut, within seconds (875)	35.7 (729)
	Quick debut, within minutes (875)	35.2 (718)
	Slow debut, within hours (875)	29.1 (595)
Constant pain (732)		55.5 (1212)
Fluctuating pain (732)		40.4 (883)
Pain aggravating over time (732)		10.8 (237)
Pain in other parts of the body (1197)		
	Head	2.5 (43)
	Throat	10.3 (177)
	Jaw	5.3 (92)
	Neck	2.5 (43)
	Between scapulars	2.2 (37)
	Back	15.2 (261)
	Left shoulder	8.5 (147)
	Right shoulder	4.2 (72)
	Left arm	24.0 (412)
	Right arm	8.4 (145)
	Left hand	1.0 (17)
	Right hand	0.3 (6)
	Stomach	7.0 (121)
	Left leg	1.7 (29)
	Right leg	1.4 (24)
	No other pain	39.3 (676)
Pain quality (1175)		
	Band-shaped	3.3 (58)
	Burning	4.4 (76)
	Stabbing	9.7 (169)
	Cramping	8.7 (151)
	Dull pain	13.9 (242)
	Fells like something is on the chest	0.7 (12)

For peer review only

Discomfort	10.2 (178)
Tingling/Stinging	5.7 (99)
Swaying	1.7 (30)
Pressuring	57.9 (1008)
Heaviness	1.0 (17)
Aching	2.4 (41)
Chest pain localization (640)	
Central pain	53.4 (1215)
Left side of chest	35.5 (809)
Right side of chest	5.1 (116)
Upper part of chest	6.7 (152)
Lower part of chest	9.0 (204)
All over the chest	11.8 (269)
Size of area affected by pain (794)	
Two inch diameter	10.7 (228)
Size of patient's palm	58.4 (1240)
Entire chest	30.9 (655)
Palpation tenderness (655)	22.3 (505)
Pain affected by movement (719)	17.0 (373)
Pain affected by breathing (692)	25.8 (573)
Troponin T (missing)	
high-sensitive Troponin T (1416)	12 (median)
Troponin T with analyse levels adjusted to Roche Cobas h232 (1416)	
Tnt <50 ng/L	88.1 (1323)
Tnt 51-100 ng/L	6.6 (99)
Tnt 101-1000 ng/L	4.5 (67)
Tnt >1000 ng/L	0.8 (12)
ECG (missing[b])	
ECG transmitted to hospital	95.5 (2785)
Sinus Rhythm, SR (235)	83.8 (2248)
Sinus Bradycardia (235)	1.3 (34)
Sinus Tachycardia (235)	8.9 (240)
Supraventricular Tachycardia, SVT (235)	0.5 (14)
Atrial Fibrillation/Flutter, AF (235)	14.3 (383)
Ventricular Tachycardia, VT (235)	0.0 (1)
Atrial Pacing (235)	1.0 (29)
Ventricular Pacing (150)	3.1 (85)
AV-block II type 1 (235)	0.0 (1)
AV-block II type 2 (235)	0.0 (1)
AV-Block III (235)	0.1 (2)
ST-Elevation (235)	6.1 (164)
ST-Depression (235)	7.6 (204)
T-wave Inversion (235)	14.5 (388)
Q-wave (235)	4.8 (130)

For peer review only

Premature Ventricular Contraction, PVC (235)	5.5 (147)
Premature Atrial Contractions, PAC (235)	3.5 (94)
Left Bundle Branch Block, LBBB (235)	6.3 (168)
Right Bundle Branch Block, RBBB (235)	6.3 (170)
Sinus rhythm and none of abnormalities above (150)	44.6 (1233)
QRS-duration (150)	92 (median)
Uninterpretable ECG (132)	0.6 (18)

*p < 0,05

**p < 0,01

***p < 0,001

[a]Logistic regression

[b]When ventricular pacing or uninterpretable remaining ECG interpretation has been

For peer review only

Patients with low-risk condition % (n)	Patients without low-risk condition % (n)	Low-risk vs without low-risk, p-value[a]	Odds Ratio	CI 95 %
68.4 (1995)	31.6 (922)			
46.2 (922)	41.1 (379)	<0.001***	0.600	0.512-0.702
53.8 (1073)	58.9 (543)			
70 (median)	76 (median)	<0.001***	0.975	0.970-0.980
20.4 (406)	7.6 (70)			
20.1 (401)	15.0 (138)	<0.001***	0.501	0.364-0.689
59.5 (1188)	77.4 (714)	<0.001***	0.287	0.219-0.376
29.0 (578)	30.2 (278)	0.515	0.945	0.797-1.121
9.7 (194)	12.3 (113)	0.039*	0.771	0.603-0.987
20.5 (409)	18.8 (173)	0.275	1.116	0.916-1.361
55.6 (1110)	63.6 (586)	<0.001***	0.719	0.612-0.844
18.6 (371)	24.8 (229)	<0.001***	0.691	0.573-0.834
19.4 (388)	20.6 (190)	0.465	0.930	0.766-1.130
7.5 (150)	9.8 (90)	0.041*	0.752	0.572-0.988
11.0 (219)	14.2 (131)	0.013*	0.745	0.590-0.939
25.0 (498)	29.2 (269)	0.016*	0.808	0.678-0.961
2.3 (46)	2.2 (20)	0.818	1.064	0.626-1.810
15.2 (303)	21.6 (199)	<0.001***	0.651	0.533-0.794
42.5 (848)	31.2 (288)	<0.001***	1.628	1.380-1.920
0 (0)	0 (0)	–	–	–
6.2 (122)	19.1 (175)	<0.001***	0.278	0.217-0.355
1.2 (23)	9.4 (87)	<0.001***	0.112	0.070-0.179
0.1 (1)	0.7 (6)	–	–	–
0.9 (18)	7.9 (73)	<0.001***	0.107	0.063-0.180
0.6 (12)	1.9 (17)	0.003**	0.321	0.152-0.674
0.8 (16)	1.3 (12)	0.198	0.610	0.287-1.295
0.5 (9)	1.8 (16)	0.001**	0.262	0.115-0.595
0.2 (3)	0.3 (3)	–	–	–
1.4 (27)	4.5 (40)	<0.001***	0.299	0.183-0.491
11.5 (182)	26.6 (204)	<0.001***	0.357	0.286-0.446
6.1 (97)	14.0 (107)	<0.001***	0.401	0.300-0.536
26.6 (421)	28.2 (214)	0.405	0.921	0.759-1.118
5.8 (92)	9.6 (73)	0.001**	0.579	0.420-0.798
40.7 (640)	52.6 (400)	<0.001***	0.619	0.520-0.737
21.8 (367)	17.4 (142)	0,004*		

For peer review only

4.3 (72)	2.7 (22)	0.369	1.266	0.756-2.120
11.9 (200)	10.7 (87)	0.470	0.889	0.647-1.222
7.3 (123)	8.1 (66)	0.072	0.721	0.505-1.030
8.7 (147)	6.7 (55)	0.857	1.034	0.718-1.490
16.1 (272)	17.9 (146)	0.022*	0.721	0.545-0.953
8.3 (140)	8.2 (67)	0.233	0.808	0.570-1.147
10.0 (169)	11.5 (94)	0.025*	0.696	0.506-0.956
7.5 (126)	9.8 (80)	0.004**	0.609	0.433-0.857
2.2 (37)	3.3 (27)	0.020*	0.530	0.311-0.903
2.0 (34)	3.6 (29)	0.004**	0.454	0.266-0.772
45.9 (592)	43.8 (271)	0.394	1.088	0.897-1.319
21.0 (306)	24.4 (173)	0.071	0.822	0.665-1.017
65.6 (956)	65.4 (463)	0.920	1.010	0.836-1.220
16.9 (246)	13.6 (96)	0.047*	1.295	1.003-1.671
37.5 (513)	32.1 (216)	0.017*	1.268	1.043-1.542
33.3 (456)	38.9 (262)	0.012*	0.783	0.647-0.949
29.2 (400)	29.0 (195)	0.909	1.012	0.826-1.240
52.9 (778)	60.9 (434)	<0.001***	0.721	0.601-0.864
42.7 (629)	35.6 (254)	0.002**	1.348	1.121-1.622
11.0 (162)	10.5 (75)	0.732	1.052	0.787-1.405
3.0 (35)	1.4 (8)	0.051	2.164	0.997-4.696
10.5 (121)	9.9 (56)	0.743	1.057	0.757-1.477
5.0 (58)	6.0 (34)	0.375	0.821	0.531-1.270
2.2 (26)	3.0 (17)	0.337	0.738	0.397-1.372
1.9 (22)	2.7 (15)	0.308	0.708	0.364-1.376
15.5 (179)	14.6 (82)	0.623	1.074	0.809-1.425
8.6 (99)	8.5 (48)	0.983	1.004	0.700-1.440
3.5 (41)	5.5 (31)	0.580	0.630	0.391-1.017
22.9 (265)	26.1 (147)	0.144	0.841	0.666-1.061
6.2 (72)	13.0 (73)	<0.001***	0.445	0.316-0.627
1.2 (14)	0.5 (3)	–	–	–
0.3 (4)	0.4 (2)	–	–	–
6.9 (80)	7.3 (41)	0.779	0.946	0.540-1.398
2.0 (23)	1.1 (6)	0.170	1.883	0.762-4.651
1.8 (21)	0.5 (3)	–	–	–
39.8 (461)	38.2 (215)	0.509	1.072	0.872-1.318
3.0 (35)	4.0 (23)	0.250	0.730	0.427-1.248
4.8 (56)	3.5 (20)	0.229	1.376	0.818-2.316
10.9 (128)	7.2 (41)	0.015*	1.577	1.093-2.277
9.0 (1255)	8.1 (46)	0.547	1.118	0.778-1.605
13.8 (162)	14.1 (80)	0.888	0.979	0.734-1.307
0.6 (7)	0.9 (5)	0.507	0.677	0.214-2.143

For peer review only

10.7 (125)	9.3 (53)	0.386	1.161	0.828-1.629
7.1 (83)	2.8 (16)	<0.001***	2.632	1.526-4.538
1.8 (21)	1.6 (9)	0.754	1.134	0.516-2.493
55.8 (654)	62.2 (354)	0.011*	0.765	0.624-0.939
0.9 (11)	1.1 (6)	0.816	0.888	0.327-2.414
2.0 (24)	3.0 (17)	0.227	0.687	0.361-1.273
50.4 (776)	59.5 (439)	<0.001***	0.693	0.580-0.827
39.3 (605)	27.6 (204)	<0.001***	1.696	1.401-2.052
5.7 (87)	3.9 (29)	0.082	1.465	0.953-2.251
6.8 (104)	6.5 (48)	0.821	1.010	0.925-1.104
9.0 (138)	8.9 (66)	0.985	1.003	0.737-1.364
11.0 (169)	13.6 (100)	0.076	0.787	0.604-1.025
12.2 (175)	7.7 (53)	0.002**	1.680	1.218-2.317
59.3 (848)	56.6 (392)	0.253	1.113	0.926-1.338
28.5 (408)	35.7 (247)	0.001**	0.719	0.592-0.872
24.3 (371)	18.3 (134)	0.001**	1.436	1.151-1.790
18.7 (277)	13.4 (96)	0.002**	1.488	1.157-1.914
26.5 (398)	24.2 (175)	0.258	1.126	0.917-1.382
9 (median)	25 (median)	<0.001***	0.979	0.974-0.984
			0.208	
96.1 (932)	73.6 (391)	<0.001***		
3.2 (31)	12.8 (68)	<0.001***	0.191	0.123-0.297
0.6 (6)	11.5 (61)	0.002**	0.041	0.018-0.096
0.1 (1)	2.1 (11)	–	–	–
95.0 (1895)	96.5 (890)	0.064	0.681	0.454-1.023
88.7 (1622)	73.3 (626)	<0.001***	2.868	2.325-3.537
1.3 (23)	1.3 (11)	0.949	0.977	0.474-2.013
7.3 (133)	12.5 (107	<0.001***	0.548	0.419-0.717
0.1 (1)	1.5 (13)	–	–	–
9.7 (177)	24.1 (206)	<0.001***	0.337	0.271-0.420
0.0 (0)	0.1 (1)	–	–	–
1.2 (22)	0.8 (7)	0.372	1.476	0.628-3.468
3.0 (57)	3.2 (28)	0.830	0.951	0.601-1.506
0.0 (0)	0.1 (1)	–	–	–
0.1 (1)	0.0 (0)	–	–	–
0 (0.0)	0.2 (2)	–	–	–
2.2 (41)	14.4 (123)	<0.001***	0.136	0.095-0.196
2.4 (44)	18.7 (160)	<0.001***	0.107	0.076-0.151
11.9 (217)	20.0 (171)	<0.001***	0.538	0.432-0.670
4.9 (89)	4.8 (41)	0.939	1.015	0.695-1.483

For peer review only

5.5 (147)	6.6 (56)	0.095	0.747	0.530-1.052
2.9 (53)	4.8 (41)	0.014*	0.592	0.391-0.898
5.2 (95)	8.5 (73)	0.001**	0.586	0.427-0.805
5.1 (93)	9.0 (77)	<0.001***	0.541	0.395-0.740
54.0 (1017)	24.5 (216)	<0.001***	3.613	3.023-4.317
92 (median)	96 (median)	<0.001***	0.988	0.985-0.992
0.5 (10)	0.9 (8)	0.260	0.585	0.230-1.488

treated as missing.

For peer review only

STROBE 2007 (v4) Statement—Checklist of items that should be included in reports of *cohort studies*

Section/Topic	Item #	Recommendation	Reported on page #
**Title and abstract**	1	(*a*) Indicate the study's design with a commonly used term in the title or the abstract	Title
		(*b*) Provide in the abstract an informative and balanced summary of what was done and what was found	Abstract
**Introduction**			
Background/rationale	2	Explain the scientific background and rationale for the investigation being reported	2-3
Objectives	3	State specific objectives, including any prespecified hypotheses	3
**Methods**			
Study design	4	Present key elements of study design early in the paper	3 + title
Setting	5	Describe the setting, locations, and relevant dates, including periods of recruitment, exposure, follow-up, and data collection	3-5
Participants	6	(*a*) Give the eligibility criteria, and the sources and methods of selection of participants. Describe methods of follow-up	3-5
		(*b*) For matched studies, give matching criteria and number of exposed and unexposed	Not applicable
Variables	7	Clearly define all outcomes, exposures, predictors, potential confounders, and effect modifiers. Give diagnostic criteria, if applicable	3-5 + supplement 1 and 2 + table 1
Data sources/ measurement	8*	For each variable of interest, give sources of data and details of methods of assessment (measurement). Describe comparability of assessment methods if there is more than one group	*3-5 + supplement 1 and 2 + table 1*
Bias	9	Describe any efforts to address potential sources of bias	4-5
Study size	10	Explain how the study size was arrived at	4
Quantitative variables	11	Explain how quantitative variables were handled in the analyses. If applicable, describe which groupings were chosen and why	5 + supplement 4+5
Statistical methods	12	(*a*) Describe all statistical methods, including those used to control for confounding	5
		(*b*) Describe any methods used to examine subgroups and interactions	Not applicable
		(*c*) Explain how missing data were addressed	Supplement 4+5
		(*d*) If applicable, explain how loss to follow-up was addressed	3 + Figure 1
		(*e*) Describe any sensitivity analyses	Not applicable
**Results**			

For peer review only

Participants	13*	(a) Report numbers of individuals at each stage of study—eg numbers potentially eligible, examined for eligibility, confirmed eligible, included in the study, completing follow-up, and analysed	5
		(b) Give reasons for non-participation at each stage	Figure 1
		(c) Consider use of a flow diagram	Figure 1
Descriptive data	14*	(a) Give characteristics of study participants (eg demographic, clinical, social) and information on exposures and potential confounders	Supplement 1, table 1
		(b) Indicate number of participants with missing data for each variable of interest	Supplement 4 + 5
		(c) Summarise follow-up time (eg, average and total amount)	Not applicable
Outcome data	15*	Report numbers of outcome events or summary measures over time	Supplement 4 + 5
Main results	16	(*a*) Give unadjusted estimates and, if applicable, confounder-adjusted estimates and their precision (eg, 95% confidence interval). Make clear which confounders were adjusted for and why they were included	Supplement 4 + 5 and Table 2
		(*b*) Report category boundaries when continuous variables were categorized	Supplement 4 + 5
		(*c*) If relevant, consider translating estimates of relative risk into absolute risk for a meaningful time period	Not applicable
Other analyses	17	Report other analyses done—eg analyses of subgroups and interactions, and sensitivity analyses	Not applicable
**Discussion**			
Key results	18	Summarise key results with reference to study objectives	10-11
**Limitations**			
Interpretation	20	Give a cautious overall interpretation of results considering objectives, limitations, multiplicity of analyses, results from similar studies, and other relevant evidence	11-12
Generalisability	21	Discuss the generalisability (external validity) of the study results	11
**Other information**			
Funding	22	Give the source of funding and the role of the funders for the present study and, if applicable, for the original study on which the present article is based	13

*Give information separately for cases and controls in case-control studies and, if applicable, for exposed and unexposed groups in cohort and cross-sectional studies.

Note: An Explanation and Elaboration article discusses each checklist item and gives methodological background and published examples of transparent reporting. The STROBE checklist is best used in conjunction with this article (freely available on the Web sites of PLoS Medicine at http://www.plosmedicine.org/, Annals of Internal Medicine at http://www.annals.org/, and Epidemiology at http://www.epidem.com/). Information on the STROBE Initiative is available at www.strobe-statement.org.

For peer review only

BMJ Open

Prehospital stratification in acute chest pain patient into high- and low-risk by emergency medical service – a prospective cohort study

Journal:	*BMJ Open*
Manuscript ID	bmjopen-2020-044938.R1
Article Type:	Original research
Date Submitted by the Author:	10-Mar-2021
Complete List of Authors:	Wibring, Kristoffer; Sahlgrenska Academy, Institute of Health and Care Sciences, Gothenburg University; Halland County, Department of Ambulance and Prehospital Care<br>Lingman, Markus; Sahlgrenska Academy, Department of molecular and clinical Medicine/Cardiology; Halland County, Department of development, Halland Hospital<br>Herlitz, Johan; University of Borås, The Prehospital Research Center Western Sweden<br>Amin, Sinan; Halland County, Department of Cardiology, Halland Hospital<br>Bång , Angela; Sahlgrenska Academy, Institute of Health and Care Sciences, Gothenburg University
**Primary Subject Heading**:	Emergency medicine
Secondary Subject Heading:	Cardiovascular medicine
Keywords:	ACCIDENT & EMERGENCY MEDICINE, Myocardial infarction < CARDIOLOGY, Cardiology < INTERNAL MEDICINE

SCHOLARONE™
Manuscripts

I, the Submitting Author has the right to grant and does grant on behalf of all authors of the Work (as defined in the below author licence), an exclusive licence and/or a non-exclusive licence for contributions from authors who are: i) UK Crown employees; ii) where BMJ has agreed a CC-BY licence shall apply, and/or iii) in accordance with the terms applicable for US Federal Government officers or employees acting as part of their official duties; on a worldwide, perpetual, irrevocable, royalty-free basis to BMJ Publishing Group Ltd ("BMJ") its licensees and where the relevant Journal is co-owned by BMJ to the co-owners of the Journal, to publish the Work in this journal and any other BMJ products and to exploit all rights, as set out in our licence.

The Submitting Author accepts and understands that any supply made under these terms is made by BMJ to the Submitting Author unless you are acting as an employee on behalf of your employer or a postgraduate student of an affiliated institution which is paying any applicable article publishing charge ("APC") for Open Access articles. Where the Submitting Author wishes to make the Work available on an Open Access basis (and intends to pay the relevant APC), the terms of reuse of such Open Access shall be governed by a Creative Commons licence – details of these licences and which Creative Commons licence will apply to this Work are set out in our licence referred to above.

Other than as permitted in any relevant BMJ Author's Self Archiving Policies, I confirm this Work has not been accepted for publication elsewhere, is not being considered for publication elsewhere and does not duplicate material already published. I confirm all authors consent to publication of this Work and authorise the granting of this licence.

For peer review only

Prehospital stratification in acute chest pain patient into high- and low-risk by emergency medical service – a prospective cohort study

Authors
Kristoffer Wibring[1, 2] kristoffer.wibring@gu.se
Markus Lingman[3, 4] markus.lingman@regionhalland.se
Johan Herlitz[5] johan.herlitz@hb.se
Sinan Amin[6] sinan.amin@regionhalland.se
Angela Bång[2] angela.bang@gu.se

1. Department of Ambulance and Prehospital Care, Region Halland, Sweden
2. Institute of Health and Care Sciences, Sahlgrenska Academy, University of Gothenburg, Göteborg, Sweden
3. Department of Molecular and Clinical Medicine/Cardiology, Institute of Medicine, Sahlgrenska Academy, University of Gothenburg, Göteborg, Sweden
4. Department of development, Halland Hospital, Sweden
5. The Prehospital Research Center Western Sweden, University of Borås, Borås, Sweden.
6. Department of cardiology, Halland Hospital, Varberg, Sweden

All authors takes responsibility for all aspects of the reliability and freedom from bias of the data presented and their discussed interpretation.

Corresponding author
Kristoffer Wibring
Ambulanssjukvården Kungsbacka
Varlabergsvägen 29
434 39 Kungsbacka
E-mail: kristoffer.wibring@gu.se
Phone: 0046701757679

For peer review only

For peer review only - http://bmjopen.bmj.com/site/about/guidelines.xhtml

ABSTRACT

Objectives: To describe contemporary characteristics and diagnoses in prehospital patients with chest pain and to identify factors suitable for the early recognition of high- and low-risk conditions.

Design: Prospective observational cohort study

Setting: Two centre study in a Swedish county EMS organisation.

Participants: Unselected inclusion of 2917 patients with chest pain contacting the EMS due to chest pain during 2018.

Primary outcome measures: Low- or high-risk condition, i.e. occurrence of time-sensitive diagnosis on hospital discharge.

Results: Of included EMS missions, 68 % concerned patients with a low-risk condition without medical need of acute hospital treatment in hindsight. Sixteen percent concerned patients with a high-risk condition in need of rapid transport to hospital care. Numerous variables with significant association with low- or high-risk conditions were found. In total high- and low-risk prediction models shared six predictive variables of which ST-depression on ECG and age were most important. Previously known risk factors such as history of acute coronary syndrome, diabetes and hypertension had no predictive value in the multivariate analyses. Some aspects of the symptoms such as pain intensity, pain in the right arm and paleness did on the other hand appear to be helpful. The AUC for prediction of low-risk candidates was 0.786 and for high-risk candidates 0.796. The addition of Troponin in a subset increased the AUC to >0.8 for both.

Conclusions: A majority of patients with chest pain cared for by the EMS suffer from a low-risk condition and have no prognostic reason for acute hospital care given their diagnosis on hospital discharge. A smaller proportion has a high-risk condition and is in need of prompt specialist care. Building models with good accuracy for prehospital identification of these groups is possible. The use of risk stratification models could make a more personalised care possible with increased patient safety.

STRENGTHS AND LIMITATIONS OF THIS STUDY

- Unselected inclusion of a close to complete county population of patients with chest pain contacting the emergency medical services.
- Low rates of missing considering the prehospital nature of data.
- Well examined cohort including data on demographics, previous medical history, symptoms, vital signs, ECG, biochemical markers and diagnosis on discharge.
- Some variables are entailed with high rates of missing when compared to studies conducted in the hospital setting.
- Study conducted in one county reduces generalisability

Keywords
Chest Pain; Emergency Medical Services; Acute Coronary Syndrome; Signs and Symptoms; Triage; Risk Assessment

For peer review only - http://bmjopen.bmj.com/site/about/guidelines.xhtml

BACKGROUND

This report is the first presenting quantitative results in a larger prospective research project (the BRIAN research programme) on improved prehospital risk assessment of patients with chest pain.

Chest pain is one of the most common chief complaints when contacting the emergency medical services (EMS). About 10-15 percent of all EMS missions concern patients with chest pain.[1 2] The role of the EMS when caring for these patients has changed since the introduction of telemedicine solutions for transmission of electrocardiograms (ECG) and direct transport to percutaneous intervention (PCI) centres for patients with ST-elevation myocardial infarction (STEMI).[3] However, these cases constitute only three percent of all patients with chest pain and one third of all patients with acute myocardial infarction (AMI).[2]

Previous studies[4] report that 15 percent of all EMS patients with chest pain have a life-threatening condition. It has been suggested that a large proportion of the patients transported by the EMS due to chest pain could be cared for outside the hospital.[5] Thus we can conclude that patients seen by the EMS before arrival in hospital due to chest pain form a mixed group with different care needs. Guidelines have focused only to a limited extent on personalising the care of patients with chest pain of other origins than STEMI despite the fact that the proportion of patients with AMI has decreased in recent decades from 30 %[6] of all patients seen by the EMS due to chest pain to 10 %.[2 4]

A more differentiated and personalised care for these chest pain patients has the potential of improving efficiency and outcome. For example, patients with non-ST elevation myocardial infarction (NSTEMI) may benefit from bypassing hospitals without PCI capabilities and bypassing the ED for direct transport to cardiac care units (CCU).[7-10] Furthermore, the undifferentiated care of the large group of patients with chest pain contributes to the problematic crowding of EDs. [11 12] Waiting in the ED increases the risk of an adverse outcome for these patients.[13 14] Consistently transporting patients with low-risk conditions to the ED also entails large costs [15] and occupy limited EMS resources. Referral to other destinations than the ED or non-conveyance for low-risk patients could provide improvements in all these aspects.

Hospital research on risk stratification is extensive but research based on data acquired in the prehospital setting is limited.[16] It is important to base prehospital clinical practice on prehospital data since patients who are seen in the prehospital setting differ from those seen at the ED.[6 17-19] This difference might affect pre-test probabilities hence diminishing the validity of the models that have been created in the ED [20-22] when applied in a new (prehospital) setting.

Previous research shows that prehospital risk assessment of patients with chest pain may be possible.[2 16 23 24] However, research is still sparse and often has methodological shortcomings, mainly regarding patient selection and using AMI as the primary endpoint. [20-22] This results in the neglect of other high-risk conditions, such as pulmonary embolism or aortic dissection, and reduces the possibility to identify low-risk patients suitable for non-conveyance. A risk assessment tool for chest pain patients, to be used in the prehospital setting, has been called for in previous research. [25-27] Such a tool might reduce arbitrariness in patient assessment and excess utilisation of emergency care.

For peer review only - http://bmjopen.bmj.com/site/about/guidelines.xhtml

The present report is mainly descriptive and investigates contemporary patient characteristics, outcome and how outcome can be predicted by information accessible in the EMS context. Future reports within this project will focus on how such information could be used and combined in a refined risk assessment tool to be used in the prehospital setting in order to stratify risks and refer patients with chest pain to an appropriate level of care.

Objectives

- To describe contemporary characteristics and diagnoses among prehospital patients with high/low risk conditions presenting with chest pain.
- To identify factors suitable for the early recognition of:
    - patients with time-sensitive conditions in need of immediate care (high-risk conditions)
    - patients with no medical need of hospital treatment, suitable for non-conveyance to hospital (low-risk conditions)
    - present data that can inform the development of a prediction tool

METHODS

Study population

This prospective cohort study was conducted in the county of Halland, Sweden. In all, 3,121 EMS missions were carried out in 2018 concerning patients, ≥18 years old, with a chief complaint of chest pain. All these missions were eligible for inclusion. After excluding patients declining to participate and patients who were lost to follow-up, 2,917 EMS missions were included in data analyses (Figure 1).

Healthcare system

The county of Halland covers an area of 5,500 km^2 and had 329,000 inhabitants in 2018. These are served by two emergency hospitals, including one with PCI capabilities. The EMS consists of eight ambulance stations with 19 ambulance vehicles. In 2018, a total of 30,672 missions were carried out by the EMS (inter-hospital site transports excluded). The EMS is staffed mainly by nurses. An ECG is routinely collected prehospitally and stored digitally using telecommunications.

Data collection

The unique personal identity number assigned to all inhabitants in Sweden made it possible to track each patient throughout the healthcare chain, from EMS mission to hospital discharge and beyond. A novel questionnaire containing fifteen items concerning patient symptoms (Supplemental file 1) was integrated in the digital journal system used by the EMS. The questionnaire was developed by the research group using the findings from two earlier reports within the research project [16 28] along with results and methodology of numerous other studies concerning patients with chest pain. By using electronic tablets, both the questionnaire and the EMS record were available at the bedside during the entire EMS mission.

The questionnaire (Supplemental file 1) contained items mainly focusing on the patients' pain narratives identifying onset, provocation/palliation, quality, radiation, severity etc. Intensity was measured using the Numerical Rating Scale (NRS) ranging from 0-10.[29] If the patient was not able to use the NRS, a Verbal Rating Scale (VRS) ranging from "no pain" to "unbearable pain" was used instead.[30] The answers from the VRS were then transformed into NRS values as follows: "no pain" = 0, "mild pain" = 2 "moderate pain" = 5, "severe pain =

For peer review only - http://bmjopen.bmj.com/site/about/guidelines.xhtml

7" and "unbearable pain" = 9.[31] The questionnaire also contained items regarding nausea/vomiting, dyspnoea, paleness and clamminess.

From the EMS record, data regarding first measured vital signs was collected. From the hospital and primary care medical records, data on diagnosis on discharge from hospital and previous medical history were retrieved.

During the EMS mission a blood sample was obtained and brought to the ED. This blood sample was analysed in hospital for high-sensitive troponin T using Roche Cobas e 601, detecting values ≥ 5 ng/l, cut-off was set to >14 ng/L. Retrieved high-sensitive troponin T-values were also converted into the following intervals <50, 51-100, 101-1000 and >1000 ng/l. In this way, we could stipulate data on troponin T as it would have been presented if Roche's device for bedside troponin T analysis, Cobas h 232, had been used by the EMS.

All registered ECGs were interpreted by either KW or SA using a pre-set ECG interpretation template (Supplemental file 2). Both were blinded to all patient data except age and sex. In cases of uncertainty, interpretation was discussed with senior research cardiologist ML and JH until consensus was reached. Two hundred ECGs were interpreted separately by both KW and SA concerning identification of ST-elevation, ST-depression, right or left bundle branch block. The Kappa coefficient for agreement was 0.810, implying almost perfect agreement.[32] The specific ECG abnormalities included in the Kappa coefficient calculation were chosen based on being most clinically relevant for identification of myocardial ischaemia according to the European Society of Cardiology (ESC).[33 34]

All data except ECG and diagnosis on discharge from hospital was extracted automatically using digital software. Diagnosis on discharge from hospital, according to ICD 10, was collected manually from the medical record by KW. Automatically retrieved data was checked extensively against the original data sources without finding any discrepancies.

Sample size

The planned sample size was 1,500 EMS missions. For a factor present at 10 % of all observations, this would entail that a relative difference of 64 % and an absolute difference of 9 % would be statistically detected. For a factor present at 20 % of all observations, this would entail that a relative difference of 49 % and an absolute difference of 7 % would be detected. For these calculations, 80 % power, significance level of 5 % (two-sided) and a 15 % incidence rate of high-risk conditions are applied. Sample size was later increased to offset the higher rates of missing than predicted in respect of specific variables that were seen early in the inclusion process.

Endpoint

The primary endpoint was a risk-classification group, in terms of low- or high-risk condition. All patients included were classified as having either a low-, intermediate- or high-risk condition as the cause of their chest pain. The adjudication was based on diagnosis on discharge from hospital according to the physician in charge. A high-risk condition was defined as a time-sensitive condition with an increased risk of death and in need of immediate care from a medical point of view in hindsight, for which transport to hospital with highest priority was called upon. An intermediate-risk condition was defined as a diagnosis probably in need of hospital care, but for which time was not judged as a critical factor. A low-risk condition was defined as a diagnosis with no medical need of hospital treatment, suitable for non-conveyance to hospital. This group also included patients with chest pain who remained

For peer review only - http://bmjopen.bmj.com/site/about/guidelines.xhtml

at home and did not visit the ED within 72 hours and who did not die within 30 days. The risk-classification of each diagnosis was carried out independently by KW, JH, ML and AB using a pre-set definition for each risk group blinded to patient characteristics. Thereafter, differences that had arisen were discussed until consensus was reached. Risk-classification was also carried out by an external cardiologist. When comparing this external risk-classification with that of the article authors, a Kappa coefficient of 0,631, substantial agreement, was reached.[32]

Statistical analysis

All variables were analysed using univariate logistic regression. Each variable was analysed twice, once to test association with high-risk conditions and once to test association with low-risk conditions. If a variable was present in fewer than six patients in any of the groups compared, no analysis was carried out. P-values below 0.05 were considered statistically significant. Variables with a statistically significant association were then included in a forward stepwise logistic regression to retain the final independent predictive variables for high- respectively low-risk conditions. These multivariate analyses were executed on a data set of 714 complete cases. Patients with ST-elevation on ECG were excluded given that there are already well-established fast-tracks for these patients. Tnt were excluded from the multivariate analyses due to high rates of missing. Additional multivariate analyses where Tnt was included were also conducted. However, the study was not powered for these specific analyses and the results should therefore be interpreted with caution. All analyses were carried out using IBM SPSS Statistics 26.

Ethical considerations

In this study, all patients eligible for inclusion were subject to an opt-out procedure. The study was approved by the Regional Ethical Review Board in Lund (Dnr 2017/212).

Patient and public involvement

Patients have not been directly involved in planning or conducting this study. Design of the questionnaire (Supplemental file 1) was partly based on patient narratives from a previous study within this research project[28] and other studies based on patient interviews. Furthermore, KW had personal contact with several patients contacting him by phone or e-mail due to the opt-out procedure, both patients wanting to opt-out and those who wanted to remain within the study. Results of the study will presented directly to those patients who requested this when contacting KW.

RESULTS

In total, 139 patients representing 170 EMS missions declined to participate. These patients represent six percent of eligible patients or five percent of eligible EMS missions. In total 2,917 EMS missions concerning 2,352 unique patients with non-traumatic chest pain were included in the data analysis. These constitute 10 % of all patient-related EMS missions during this time period (inter-hospital transports excluded). Priority 1 (highest priority) was given by the emergency medical dispatcher to 63 % of all missions included, and these constitute 11 % of all priority 1-missions within the region. The EMS nurse then triaged patients on scene using the Rapid Triage and Treatment System (RETTS) [35,36] to the highest priority in ten percent of cases and to the lowest priority in four percent. EMS missions were equally distributed regarding sex. Median age was 72 years (Q25-Q75, 58-82) (Supplemental file 3).

For peer review only - http://bmjopen.bmj.com/site/about/guidelines.xhtml

In total, 16 % were classified with a high-risk condition, 16 % had an intermediate-risk condition and 68 % had a low-risk condition (Table 1). The all-cause mortality rate within 30 days was 2.9 % (each unique patient counted only once). In the high-risk group mortality rate within 30 days was 8.9 % and in the low-risk group 0.5 %.

Table 1 - Distribution of risk classification and diagnoses by sex and age

		Sex		Age group		
	**All % (n)**	**Men % (n)**	**Women % (n)**	**≤50 % (n)**	**51-64 % (n)**	**≥65 % (n)**
	100 (2917)	50.2 (1465)	49.8 (1452)	16.3 (476)	18.5 (539)	65.2 (1902)
High-risk conditions	16.0 (467)	20.3 (298)	11.6 (169)	6.1 (29)	14.8 (80)	18.8 (358)
NSTEMI	6.7 (194)	8.2 (120)	5.1 (74)	2.1 (10)	5.0 (27)	8.3 (157)
STEMI	4.3 (127)	5.9 (87)	2.8 (40)	1.3 (6)	6.5 (35)	4.5 (86)
Unstable angina pectoris	2.1 (60)	2.8 (41)	1.3 (19)	0.4 (2)	1.5 (8)	2.6 (50)
Pulmonary embolism	0.8 (24)	1.0 (14)	0.7 (10)	0.8 (4)	0.7 (4)	0.8 (16)
Undefined AMI*	0.4 (11)	0.7 (10)	0.1 (1)	0.0 (0)	0.2 (1)	0.5 (10)
MINCA/MINOCA	0.2 (7)	0.1 (1)	0.4 (6)	0.0 (0)	0.9 (5)	0.1 (2)
Aortic dissection/aneurysm	0.2 (7)	0.4 (6)	0.1 (1)	0.0 (0)	0.0 (0)	0.4 (7)
Severe arrhythmias and conducting disorders**	0.2 (7)	0.3 (5)	0.1 (2)	0.2 (1)	0.0 (0)	0.3 (6)
Takotsubo	0.2 (5)	0.1 (1)	0.3 (4)	0.4 (2)	0.0 (0)	0.2 (3)
Sepsis	0.1 (4)	0.2 (3)	0.1 (1)	0.2 (1)	0.0 (0)	0.2 (3)
Stroke/TIA	0.1 (4)	0.1 (2)	0.1 (2)	0.4 (2)	0.0 (0)	0.1 (2)
Gastric ulcer with perforation/bleeding	0.1 (4)	0.1 (1)	0.2 (3)	0.0 (0)	0.0 (0)	0.2 (4)
Pulmonary oedema	0.1 (2)	0.0 (0)	0.1 (2)	0.0 (0)	0.0 (0)	0.1 (2)
Other, high risk	0.4 (11)	0.5 (7)	4 0.3	0.2 (1)	0.0 (0)	0.5 (10)
Intermediate-risk conditions	15.6 (455)	16.7 (245)	14.5 (210)	8.6 (41)	10.8 (58)	18.7 (356)
Atrial fibrillation/flutter	3.8 (112)	3.9 (57)	3.8 (55)	0.6 (3)	3.0 (16)	4.9 (93)
Heart failure (without pulmonary oedema)	2.1 (60)	2.3 (34)	1.8 (26)	0.0 (0)	0.2 (1)	3.1 (59)
Pneumonia	1.8 (52)	1.7 (25)	1.9 (27)	0.2 (1)	0.7 (4)	2.5 (47)
Myocarditis, pericarditis,	1.1 (31)	1.6 (23)	0.6 (8)	2.5 (12)	1.3 (7)	0.6

For peer review only

endocarditis						(12)
Syncope and collapse	0.6 (18)	0.5 (7)	0.8 (11)	0.4 (2)	0.7 (4)	0.6 (12)
Aortic valve stenosis	0.6 (17)	0.7 (10)	0.5 (7)	0.0 (0)	0.2 (1)	0.8 (16)
Tumour	0.6 (17)	0.5 (7)	0.7 (10)	0.2 (1)	0.4 (2)	0.7 (14)
Chronic obstructive pulmonary disease (COPD)	0.5 (14)	0.6 (9)	0.3 (5)	0.0 (0)	0.0 (0)	0.7 (14)
Infection, intermediate risk	0.5 (14)	0.5 (7)	0.5 (7)	0.4 (2)	0.6 (3)	0.5 (9)
Supraventricular tachycardia	0.4 (13)	0.3 (5)	0.1 (2)	0.6 (3)	0.7 (4)	0.3 (6)
Cholelithiasis	0.4 (13)	0.5 (7)	0.4 (6)	0.4 (2)	0.4 (2)	0.5 (9)
Pancreatitis	0.3 (9)	0.3 (5)	0.3 (4)	0.6 (3)	0.2 (1)	0.3 (5)
Electrolyte disturbance	0.2 (7)	0.5 (7)	0.0 (0)	0.2 (1)	0.0 (0)	0.3 (6)
Convulsions and seizures	0.2 (6)	0.3 (4)	0.1 (2)	0.6 (3)	0.4 (2)	0.1 (1)
Diverticulitis	0.1 (4)	0.3 (4)	0.0 (0)	0.0 (0)	0.0 (0)	0.2 (4)
Other, intermediate risk	2.3 (67)	2.5 (37)	2.1 (30)	1.7 (8)	2.0 (11)	2.5 (48)
Low-risk conditions	68.4 (1995)	62.9 (922)	73.9 (1073)	85.3 (406)	74.4 (401)	62.5 (1188)
Chest pain, unspecified	41.5 (1211)	39.0 (572)	44.0 (639)	52.7 (251)	50.8 (274)	36.1 (686)
Did not convey (No related ED visit in 72 h or death within 30 days)	9.5 (276)	8.1 (118)	0.3 (158)	16.4 (78)	8.7 (47)	7.9 (151)
Angina pectoris (unstable and spasm induced angina excluded)	3.1 (90)	3.3 (48)	2.9 (42)	0.4 (2)	1.5 (8)	4.2 (80)
Abdominal and pelvic pain	2.1 (62)	1.4 (21)	41 (2.8)	2.7 (13)	1.7 (9)	2.1 (40)
Infection, low risk	1.6 (47)	1.3 (19)	1.9 (28)	1.1 (5)	1.9 (10)	1.7 (32)
Gastritis/Gastro-oesophageal reflux disease	1.4 (41)	1.2 (18)	1.6 (23)	2.1 (10)	1.3 (7)	1.3 (24)
Dyspnoea and coughing	1.4 (40)	1.5 (22)	1.2 (18)	1.7 (8)	1.1 (6)	1.4 (26)
Palpitation and benign arrhythmias	1.2 (35)	0.8 (11)	1.7 (24)	1.7 (8)	1.1 (6)	1.1 (21)
Anxiety and other mental disorders	0.9 (25)	0.6 (9)	1.1 (16)	2.7 (13)	0.2 (1)	0.6 (11)
Other pain	0.7 (19)	0.6 (9)	0.7 (10)	0.2 (1)	1.3 (7)	0.6 (11)
Anaemia	0.5 (16)	0.5 (8)	0.6 (8)	0.2 (1)	0.7 (4)	0.6 (11)
Vertigo	0.5 (14)	0.5 (7)	0.5 (7)	0.4 (2)	0.2 (1)	0.6 (11)

For peer review only - http://bmjopen.bmj.com/site/about/guidelines.xhtml

Back pain	0.3 (10)	0.2 (3)	0.5 (7)	0.2 (1)	0.2 (1)	0.4 (8)
Orthostatic hypotension	0.3 (10)	0.5 (8)	0.1 (2)	0.0 (0)	0.0 (0)	0.5 (10)
Hypertension	0.3 (10)	0.1 (2)	0.6 (8)	0.2 (1)	0.2 (1)	0.4 (8)
Mental and behavioural disorders due to alcohol	0.3 (10)	0.6 (9)	0.1 (1)	0.4 (2)	0.7 (4)	0.2 (4)
Headache	0.2 (6)	0.1 (1)	0.3 (5)	0.6 (3)	0.2 (1)	0.1 (2)
Other, low risk	2.5 (74)	2.6 (38)	2.5 (36)	1.5 (7)	2.6 (14)	2.8 (53)

*Type of myocardial infarction not stated in patient medical record
**Ventricular tachycardia, AV-block III etc.

For peer review only

The cohort was diagnosed with a wide range of medical conditions involving most of the body's organ systems. Most common was musculoskeletal or unspecified chest pain. This diagnosis was given in 41.5 % of all EMS missions. Twelve percent were given an AMI diagnosis on discharge from hospital. In almost ten percent of the EMS missions, the patient was not conveyed to the ED and did not have a related ED visit within 72 hours or died within 30 days (Table 1).

Patient characteristics and patient presentation differ substantially within the cohort. However, there are some dominating aspects. Patients are old and have a substantial comorbidity – most commonly hypertension or mental disorders. They commonly describe central, constant, pressuring pain about the size of a palm starting several hours earlier during rest. Vital signs are most commonly unaffected. In most cases, the ECG presents alterations known to be associated with underlying pathological conditions. These are the most common findings in patients with both low- and high-risk conditions (Supplemental file 4 and 5).

Risk prediction
In total, 26 variables showed significantly increased odds and 16 variables showed significantly decreased odds ratio of having a high-risk condition in the univariate analyses (Supplemental file 4). When predicting low-risk conditions, eleven variables showed significantly increased odds ratio for a low-risk condition whereas 37 variables showed a significant increase in the ability to predict the absence of a low-risk condition in the univariate analyses (Supplemental file 5).

When including these variables in a complete cases multivariate analyses 13 and 14 variables remained for high-risk and low-risk prediction respectively. The predictive variables were largely common to both low- and high-risk prediction, with old age and ST-depression as the most important predictors. Previous medical history, except psychiatric disorders, atrial fibrillation/flutter and chronic obstructive pulmonary disease (COPD), had no predictive value in neither high- or low-risk models. In total high- and low-risk prediction models shared six predictive variables, which are written in italics in Table 2. These variables were; age, premature atrial contractions, ST-depression, pain in right arm, paleness and previous medical history of atrial fibrillation/flutter. The area under the receiving operating characteristic curve for prediction of low-risk conditions was 0.786 and 0.796 for high-risk conditions (Figure 2). The additional analyses where Tnt were included resulted in a high-risk model with an AUC of 0.847 and a low-risk model with an AUC of 0.884. Accuracy, in terms of AUC, when

adding Tnt thereby increased by 8 % for high-risk prediction and 11 % for low-risk prediction.

Table 2 - Predictors of high- and low-risk conditions after multivariate analysis			
	**Odds Ratio**	**CI 95 %**	**p-value***
**High-risk predictors**			
Age ≤50	-	-	<0.001
Age 51-64	18.98	5.47-65.95	0,000
Age ≥65	7.87	2.12-29.24	0.002
ECG Premature Atrial Contractions, PAC	4.52	1.67-12.25	0.003
ECG ST-depression	3.52	1.63-7.58	0.001
Pain in right arm	2.80	1.48-5.32	0.001
Paleness	2.44	1.44-4.14	<0.001
Male	2.01	1.29-3.14	0.002
Debut during activity	2.01	1.22-3.32	0.006
Pain intensity according to Numeric Rating Scale >8	2.38	1.10-5.13	0.026
ECG T-wave inversion	2.01	1.06-3.82	0.032
Time elapsed since pain onset >3 hours	0.49	0.31-0.77	0.001
Previous atrial fibrillation/flutter	0.42	0.25-0.72	0.001
Previous COPD	0.41	0.18-0.94	0.035
Size of area affected by pain; two inch diameter	0.35	0.13-0.93	0.035
**Low-risk predictors**			
Previous atrial fibrillation/flutter	2.46	1.47-4.11	<0.001
Right sided chest pain	3.88	1.30-11.59	0.015
Previous psychiatric diagnosis (any type)	1.70	1.14-2.54	0.009
Sinus rhythm without abnormalities	1.70	1.11-2.60	0.014
Paleness	0.39	0.25-0.63	<0.001
Breathing rate ≥25 breaths/min	0.45	0.24-0.82	0.009
ECG Atrial Fibrillation/Flutter	0.42	0.23-0.76	0.004
Age ≤50	-	-	<0.001
Age 51-64	0.40	0.18-0.87	0.021
Age ≥65	0.22	0.11-0.45	<0.001
Pain in right arm	0.28	0.15-0.50	<0.001
Pain in right shoulder	0.33	0.14-0.80	0.014
ECG Premature Atrial Contractions, PAC	0.36	0.14-0.94	0.036
Oxygen saturation ≤91 %	0.32	0.12-0.84	0.020
ECG ST-depression	0.18	0.08-0.42	<0.001
Body temperature >38.0	0.15	0.04-0.63	0.009

*Stepwise forward logistic regression, complete cases, entery $p < 0.05$, removal $p < 0.10$

Italics = Variable common for both high- and low-risk condition prediction models

For peer review only - http://bmjopen.bmj.com/site/about/guidelines.xhtml

DISCUSSION

To our best knowledge, this is the first prospective population wide study of unselected prehospital chest pain patients beyond acute coronary syndromes. This is important since the rule out of acute myocardial infarction alone does not allow EMS to alter conveyance. We present models that can identify high- and low-risk underlying conditions with high accuracy and show that most of these patients suffered from a low-risk condition while sixteen percent had an underlying condition with high risk, such as AMI, pulmonary embolism, aortic dissection, arrhythmias, sepsis and bleeding gastric ulcer. This variety of diagnoses stresses the incentive to have broad inclusion criteria in contrast with previous reports.

Accuracy in terms of AUC and ROC curve shape is quite similar when comparing low- and high-risk prediction models. Both models also seem to improve substantially if adding Tnt.

The present paper aims to inform future risk assessment tools with capabilities to rule in high-risk conditions and rule out low-risk separately. To that end and opposite to expectations classic symptoms of AMI like radiation to left arm or jaws, constant pain, clamminess and dyspnoea did not differ between high- and low-risk conditions in the multivariate analyses. The same was true for previously known risk factors such as history of hypertension, ACS and diabetes. [34] These findings are surprising. It may be explained by our study using a wider range of diagnoses as endpoint compared with previous studies focusing on AMI. [5 37] This is strengthened by ad-hoc analyses showing increased significance for above-mentioned factors when using AMI as endpoint instead of high-risk condition. It may also be explained by prehospital patients having a higher comorbidity compared to patients at the ED [6] and that previous medical history therefore is less useful to discriminate high- from low-risk conditions in the prehospital setting. However, most of these factors differ in the univariate analyses but not in the multivariate ones. Thereby, these factors may still have a predictive value, but when combined with others predictive factors they become less important. Future risk models may need to cover other variables, with ability to contribute to accuracy, than traditional risk factors for AMI. The use of artificial intelligence may be one way to identify such variables.

In both the low- and high-risk models, premature atrial complex (PAC) on ECG have a predictive value. This finding was not expected. Once again, this may be explained by not using AMI as endpoint. This is strengthened by ad-hoc analyses using AMI as endpoint where PAC did not turn out significantly. Given the multiple analyses and the quite wide confidence intervals, this may also be a chance finding.

Vital signs seem to have little predictive value when identifying high-risk conditions. To some extent, this may be explained by strongly deviating vital signs were quite rare within the cohort. However, tachypnoea, low oxygen saturation and fever all reduced the odds for a low-risk condition. Deviating vital signs thereby seem more useful when ruling out patients from the low-risk group rather than to predict high-risk conditions.

One main finding of this study, and maybe also most relevant in clinical care, is that more than two thirds of all EMS missions concerning patients with chest pain were classified as low-risk, i.e. having no need for acute transportation to hospital given the diagnosis on discharge. This implies the substantial magnitude of the impact of a predictive model identifying them with high accuracy early in the care chain. Diverting only a proportion of these low-risk patients away from the ED to less resource intensive venues would probably reduce healthcare costs, EMS workload and ED crowding.

For peer review only - http://bmjopen.bmj.com/site/about/guidelines.xhtml

Among chest pain patients, ten percent remained at home, despite EMS personnel having neither mandate nor instruments to provide such recommendations. This finding indicates that even today, EMS personnel or the patients themselves sometimes deem transport to the ED as inappropriate, despite the patients' chest pain. However, little is known about the appropriateness of EMS decision-making when suggesting this type of triage.

Among the sixteen percent of EMS missions with patients with high-risk conditions, ACS constituted the major part. The portion of high-risk conditions is in line with previous findings.[2 4] Importantly STEMI constitute less than one third of all missions concerning high-risk conditions, confirming work by Terkelsen et al.[2] The fairly low incidence of STEMI shows the importance of taking other high-risk diagnoses into account when risk-assessing patients with chest pain. This would for example enable direct transport to CCU, hospitals with PCI or capabilities for thoracic surgery.[10]

Seeking to discriminate between high- and low-risk conditions, we also looked for information that would *not* support this aim. In this report, we therefore acknowledge that symptoms typically associated with myocardial ischemia, such as central pain, pressuring pain quality, left arm radiation and affected breathing, are common in both groups. Notably most patients with such symptoms were found in the low risk group. This may limit the usefulness of some symptoms previously reported to be of value in risk assessment and triage. However, other aspects of symptoms such as pain intensity, pain in the right arm and the presence of paleness were helpful in the discrimination of high versus low risk.

Risk assessment in the prehospital setting may serve several purposes. Conveyance decision based on the possibility to identify both low-risk patients suitable for non-hospital care/non-conveyance and high-risk patients in need of prompt specialist care are separate issues.
To further inform a future model supporting the conveyance decision a multivariate analysis was reported. The results of the multivariate analyses presented in this study could form the basis for such a decision support tool. The c-statistics indicate that such a tool could reach a level of accuracy appropriate for clinical use. The fact that several classic symptoms and previously known risk factors of AMI turned out to be of little predictive value indicates that previous risk prediction tools developed in the hospital setting such as the HEART-score and RETTS may be less accurate and invalid when used in the prehospital setting. It is also important to acknowledge that a single factor in itself can constitute grounds for prompt transport to hospital, such as strongly deviating vital signs.

The models presented in this study need to be refined before tested further. Adding Tnt to the analyses seems to improve accuracy substantially and needs to be investigated further. Analyses on imputated data or a new data set with more complete data regarding Tnt is needed. It would also be preferable to develop a combined model predicting both low- and high-risk conditions, given that good c-statistics may be retained. It would also be of interest to examine the accuracy of reduced models with fewer variables. A combined model including fewer variables would ease clinical usage. The result of such a refined model development will be presented in upcoming reports within the research programme. Prediction models developed using artificial intelligence will also be presented. External validation of the final prediction model(s) is planned to be carried out in multi-centre studies if upcoming results indicate that clinical usage would be appropriate. The prediction model(s) could be integrated in a mobile application or other electronic device to improve clinical usability.

For peer review only - http://bmjopen.bmj.com/site/about/guidelines.xhtml

Strengths and limitations

A key strength is the complete picture of the study sample as data provides information on demographics, medical history, a wide range of symptoms, vital signs, ECG, biochemical markers and diagnosis on discharge. However, some variables included entail rather high rates of missing information (Supplemental file 4 and 5). When analysing the distribution of missing data, no skewness of clinical relevance could be identified. This is also confirmed by the fact that the results of the univariate analyses to a large extent confirm previous findings concerning which variables were associated with high-risk conditions. This holds true also for variables with the highest rates of missing e.g. pain intensity [38], pain radiation [38], quality of pain [21] and biochemical markers (Tnt).[39]

Data collection in the prehospital setting is known to be challenging. The situation is often perceived as stressful by the patient, the patient may be physically or mentally unable to provide the information requested, personnel resources are limited and protocol compliance among EMS personnel has been reported to be low.[40 41] All these components contribute to the finding that prehospital research often suffers from high rates of missing information. In this study, we observed that missing rates were highest regarding data where the questionnaire (Supplemental file 1) required the user to write the answer rather than tick a box. The rates of missing data in these cases can thereby probably mainly be explained by limited protocol compliance among EMS personnel.

The high rate of missing data regarding Tnt is a major limitation, since this complicates the inclusion of Tnt in the multivariate analyses. Upcoming reports, using data that are more complete on Tnt, will present more comprehensive prediction models. There are two main reasons for the high rates of missing data on Tnt. Firstly, no blood sample analysis was done if the patient remained at home. Secondly, a blood sample could not be analysed if the patient was transported directly to a hospital outside the county. However, these reasons for not obtaining data on Tnt are not a real-life problem if using a device for prehospital bedside troponin T analysis.

Considering that this was a study based on prehospital data, the rates of missing information must be regarded as low and therefore data may be looked upon as comparatively comprehensive. The varying rates of missing information show possible difficulties using certain variables in a prehospital context. For this reason, it is also important to be careful when selecting variables to be included in a planned decision support tool. A tool requiring data on variables which are difficult to collect in the prehospital setting will reduce personnel compliance and thereby be of less clinical value.

This study use risk-classification group as end-point. The classification was based on extensive discussions among experienced colleagues trying to reach a consensus. Risk-classification was also carried out by an external cardiologist, with substantial Cohen's kappa agreement. However, there are not absolute distinctions between risk-classification groups. For example, aortic stenosis and acute pancreatitis can present in different ways. Patients with these diagnoses can be in need of prompt hospital care but in other cases, time is of little importance. Abnormal vital signs may be helpful to discern such cases. Furthermore, there may be other reasons (for example frailty) or intensive pain, which may force the EMS personnel to transport a patient with a low-risk condition to hospital. The objective of the predictions models is to guide the EMS personnel in their decision making, not to replace clinical judgement.

For peer review only - http://bmjopen.bmj.com/site/about/guidelines.xhtml

Generalisability

The study is strengthened by the nearly complete and unselected inclusion of all EMS missions within the county concerning patients with chest pain during 2018. This increases the generalisability of the results. However, the use of data from only one EMS organisation negatively impacts the external validity. We have no reason to believe that our result is not applicable in other counties in Sweden. The current county includes both urban and rural areas, with a wide range of socio-economic conditions and ethnical diversity. However, we don't have access to specific data on these factors for included patients which is a limitation given that those factors are reported to affect prehospital care of patients with chest pain. [42] Generalisation of the results beyond Sweden should be done with care, at least outside the western world, given differences in EMS organisations, care financing and EMS contact behaviour.

The level of competence among EMS personnel and equipment available differ between different EMS organisations and countries. This should be considered when discussing future clinical implications. With a minimum of training, included variables are easy to obtain, at least if using external ECG interpretation via telemedicine solutions. Future prediction models should therefore be possible to use by EMS personnel with different competence.

Some of included variables are subjective, either from a patient or personnel perspective. For example, what is regarded as pain in right shoulder may differ among patients. Different EMS personnel may assess paleness differently, etc. The use of such variables should therefore be limited. Instead, more objective parameters such as, ECG, previous medical history, age and sex should be promoted in future models to improve reliability.

CONCLUSIONS

A majority of patients with chest pain cared for by the EMS suffer from a low-risk condition and have no prognostic reason for acute hospital care given their diagnosis on hospital discharge. A smaller proportion has a high-risk condition and is in need of prompt specialist care. Building models with good accuracy for prehospital identification of these groups is possible. Models optimising rule out will differ from models optimising rule in. However, ECG findings and age are cornerstones in both. Importantly previously known risk factors such as history of acute coronary syndrome, diabetes or hypertension did not have a predictive value in such models. On the other hand previous history of psychiatric disorders and atrial fibrillation/flutter are of importance when risk stratifying prehospital patients with chest pain. The use of risk stratification models will make a more personalised care possible with increased patient safety. More research on the value of adding Tnt and how to refine such risk stratification models is needed before clinical testing.

List of abbreviations

ACS - Acute coronary syndrome
AMI - Acute myocardial infarction
CABG - Coronary artery bypass graft
CCU - Cardiac care units
COPD – Chronic obstructive pulmonary disease
ECG - Electrocardiogram
ED - Emergency department
EMS - Emergency medical services
ESC - European Society of Cardiology

For peer review only - http://bmjopen.bmj.com/site/about/guidelines.xhtml

NSTEMI - Non-ST elevation myocardial infarction
PAC - Premature atrial complex
PCI - Percutaneous intervention
RETTS - Rapid Emergency Triage and Treatment System
STEMI - ST-elevation myocardial infarction
TNT - Troponin T

Contributors

KW, ML, JH, SA and AB designed the study and planned the data collection. KW, ML, JH and SA interpreted the ECG's. Data analysis was carried out by KW. KW, ML, JH and AB contributed in writing the manuscript. All authors read and approved the final manuscript.

Funding

This study has been funded by the Department of Ambulance and Prehospital Care, Region Halland and the Scientific Council of Region Halland (HALLAND-209901). The funding bodies had no role in the design, conduct, interpretation or writing of the report on this research.

Competing interests

The authors declare that they have no competing interests.

Patient consent for publication

Not required.

Ethics approval

The study was approved by the Regional Ethical Review Board in Lund (Dnr 2017/212). All patients were given the opportunity to withdraw their participation using an opt-out procedure.

Data availability statement

The datasets generated and analysed during the current study are not publicly available due the integrity of patient privacy but are available from the corresponding author on reasonable request and if approved by the Regional Ethical Review Board in Lund.

Acknowledgements

We want to thank the following persons:

- The EMS personnel of Region Halland for their help with data collection.
- Thomas Thorsson, Vinh Norberg Nguyen and Sasa Pejicic for data extraction.
- Anders Holmén and Ulf Strömberg for statistical support.

Tables, figures and supplemental material

Figure 1 - Flow chart of inclusion process
Figure 2 - Schematic presentation of prediction models accuracy
Table 1 - Distribution of risk classification and diagnoses by sex and age
Table 2 - Prediction models for high- and low-risk conditions
Supplemental material 1 - Analog version of study questionnaire
Supplemental material 2 - ECG interpretation manual
Supplemental material 3 - Description of study sample

For peer review only - http://bmjopen.bmj.com/site/about/guidelines.xhtml

Supplemental material 4 - Prediction of high-risk conditions, univariate analyses
Supplemental material 5 - Prediction of low-risk conditions, univariate analyses

For peer review only

REFERENCES

1. Clawson J, Olola C, Heward A, et al. The Medical Priority Dispatch System's ability to predict cardiac arrest outcomes and high acuity pre-hospital alerts in chest pain patients presenting to 9-9-9. *Resuscitation* 2008;78(3):298-306. doi: 10.1016/j.resuscitation.2008.03.229
2. Pedersen CK, Stengaard C, Friesgaard K, et al. Chest pain in the ambulance; prevalence, causes and outcome - a retrospective cohort study. *Scand J Trauma Resusc Emerg Med* 2019;27(1):84. doi: 10.1186/s13049-019-0659-6 [published Online First: 2019/08/29]
3. Hagiwara MA, Bremer A, Claesson A, et al. The impact of direct admission to a catheterisation lab/CCU in patients with ST-elevation myocardial infarction on the delay to reperfusion and early risk of death: results of a systematic review including meta-analysis. *Scandinavian Journal of Trauma Resuscitation & Emergency Medicine* 2014;22 doi: 10.1186/s13049-014-0067-x
4. Rawshani A, Larsson A, Gelang C, et al. Characteristics and outcome among patients who dial for the EMS due to chest pain. *International Journal of Cardiology* 2014;176(3):859-65. doi: 10.1016/j.ijcard.2014.08.004
5. Ishak M, Ali D, Fokkert MJ, et al. Fast assessment and management of chest pain patients without ST-elevation in the pre-hospital gateway (FamouS Triage): ruling out a myocardial infarction at home with the modified HEART score. *Eur Heart J Acute Cardiovasc Care* 2017:2048872616687116. doi: 10.1177/2048872616687116 [published Online First: 2017/01/01]
6. Thang ND, Karlson BW, Bergman B, et al. Characteristics of and outcome for patients with chest pain in relation to transport by the emergency medical services in a 20-year perspective. *American Journal of Emergency Medicine* 2012;30(9):1788-95. doi: 10.1016/j.ajem.2012.02.014
7. Ravn-Fischer A, Karlsson T, Johanson P, et al. Prehospital ECG signs of acute coronary occlusion are associated with reduced one-year mortality. *Int J Cardiol* 2013;168(4):3594-8. doi: 10.1016/j.ijcard.2013.05.064 [published Online First: 2013/05/30]
8. Beygui F, Castren M, Brunetti ND, et al. Pre-hospital management of patients with chest pain and/or dyspnoea of cardiac origin. A position paper of the Acute Cardiovascular Care Association (ACCA) of the ESC. *Eur Heart J Acute Cardiovasc Care* 2015 doi: 10.1177/2048872615604119 [published Online First: 2015/08/27]
9. Rasmussen MB, Stengaard C, Sørensen JT, et al. Predictive value of routine point-of-care cardiac troponin T measurement for prehospital diagnosis and risk-stratification in patients with suspected acute myocardial infarction. *Eur Heart J Acute Cardiovasc Care* 2017:2048872617745893. doi: 10.1177/2048872617745893 [published Online First: 2017/12/01]
10. Stengaard C, Sørensen JT, Rasmussen MB, et al. Editor's Choice-Acute versus subacute angiography in patients with non-ST-elevation myocardial infarction - the NONSTEMI trial phase I. *Eur Heart J Acute Cardiovasc Care* 2017;6(6):490-99. doi: 10.1177/2048872616648468 [published Online First: 2016/05/06]
11. Carter EJ, Pouch SM, Larson EL. The relationship between emergency department crowding and patient outcomes: a systematic review. *J Nurs Scholarsh* 2014;46(2):106-15. doi: 10.1111/jnu.12055 [published Online First: 2013/12/19]
12. Patton GG, Thakore S. Reducing inappropriate emergency department attendances--a review of ambulance service attendances at a regional teaching hospital in Scotland. *Emergency medicine journal : EMJ* 2013;30(6):459-61. doi: 10.1136/emermed-2012-201116 [published Online First: 2012/07/16]

For peer review only - http://bmjopen.bmj.com/site/about/guidelines.xhtml

13. Pines JM, Pollack CV, Diercks DB, et al. The Association Between Emergency Department Crowding and Adverse Cardiovascular Outcomes in Patients with Chest Pain. *Academic Emergency Medicine* 2009;16(7):617-25. doi: 10.1111/j.1553-2712.2009.00456.x
14. Kulstad EB, Kelley KM. Overcrowding is associated with delays in percutaneous coronary intervention for acute myocardial infarction. *Int J Emerg Med* 2009;2(3):149-54. doi: 10.1007/s12245-009-0107-x [published Online First: 2009/06/05]
15. van Dongen DN, Ottervanger JP, Tolsma R, et al. In-Hospital Healthcare Utilization, Outcomes, and Costs in Pre-Hospital-Adjudicated Low-Risk Chest-Pain Patients. *Appl Health Econ Health Policy* 2019 doi: 10.1007/s40258-019-00502-6 [published Online First: 2019/08/06]
16. Wibring K, Herlitz J, Christensson L, et al. Prehospital factors associated with an acute life-threatening condition in non-traumatic chest pain patients - A systematic review. *Int J Cardiol* 2016;219:373-9. doi: 10.1016/j.ijcard.2016.06.066
17. Lavery T, Greenslade JH, Parsonage WA, et al. Factors influencing choice of pre-hospital transportation of patients with potential acute coronary syndrome: An observational study. *Emerg Med Australas* 2017;29(2):210-16. doi: 10.1111/1742-6723.12735 [published Online First: 2017/01/25]
18. Boothroyd LJ, Lambert LJ, Segal E, et al. Comparison of outcomes of ambulance users and nonusers in ST elevation myocardial infarction. *Am J Cardiol* 2014;114(9):1289-94. doi: 10.1016/j.amjcard.2014.07.060 [published Online First: 2014/09/10]
19. Thuresson M, Jarlöv MB, Lindahl B, et al. Factors that influence the use of ambulance in acute coronary syndrome. *Am Heart J* 2008;156(1):170-6. doi: 10.1016/j.ahj.2008.01.020 [published Online First: 2008/03/14]
20. Ishak M, Ali D, Fokkert MJ, et al. Fast assessment and management of chest pain without ST-elevation in the pre-hospital gateway: rationale and design. *Eur Heart J Acute Cardiovasc Care* 2015;4(2):129-36. doi: 10.1177/2048872614549738 [published Online First: 2014/09/08]
21. Stopyra JP, Harper WS, Higgins TJ, et al. Prehospital Modified HEART Score Predictive of 30-Day Adverse Cardiac Events. *Prehosp Disaster Med* 2018;33(1):58-62. doi: 10.1017/S1049023X17007154 [published Online First: 2018/01/10]
22. Alghamdi A, Howard L, Reynard C, et al. Enhanced triage for patients with suspected cardiac chest pain: the History and Electrocardiogram-only Manchester Acute Coronary Syndromes (HE-MACS) decision aid. *Eur J Emerg Med* 2018 doi: 10.1097/MEJ.0000000000000575 [published Online First: 2018/10/04]
23. Rawshani N, Rawshani A, Gelang C, et al. Could ten questions asked by the dispatch center predict the outcome for patients with chest discomfort? *Int J Cardiol* 2016;209:223-5. doi: 10.1016/j.ijcard.2016.02.011 [published Online First: 2016/02/22]
24. van Dongen DN, Tolsma RT, Fokkert MJ, et al. Pre-hospital risk assessment in suspected non-ST-elevation acute coronary syndrome: A prospective observational study. *Eur Heart J Acute Cardiovasc Care* 2018:2048872618813846. doi: 10.1177/2048872618813846 [published Online First: 2018/11/23]
25. Thang ND, Sundstrom BW, Karlsson T, et al. ECG signs of acute myocardial ischemia in the prehospital setting of a suspected acute coronary syndrome and its association with outcomes. *American Journal of Emergency Medicine* 2014;32(6):601-05. doi: 10.1016/j.ajem.2014.03.006
26. Saddichha S, Saxena MK. Is every chest pain a cardiac event? : an audit of patients with chest pain presenting to emergency services in India. *Internal and emergency*

For peer review only - http://bmjopen.bmj.com/site/about/guidelines.xhtml

medicine 2009;4(3):235-9. doi: 10.1007/s11739-009-0246-3 [published Online First: 2009/04/16]

27. Nehme Z, Boyle MJ, Brown T. Diagnostic accuracy of prehospital clinical prediction models to identify short-term outcomes in patients with acute coronary syndromes: a systematic review. *J Emerg Med* 2013;44(5):946-54.e6. doi: 10.1016/j.jemermed.2012.07.078 [published Online First: 2013/01/13]
28. Wibring K, Herlitz J, Lingman M, et al. Symptom description in patients with chest pain - a qualitative analysis of emergency medical calls involving high-risk conditions. *J Clin Nurs* 2019 doi: 10.1111/jocn.14867 [published Online First: 2019/04/02]
29. Hawker GA, Mian S, Kendzerska T, et al. Measures of adult pain: Visual Analog Scale for Pain (VAS Pain), Numeric Rating Scale for Pain (NRS Pain), McGill Pain Questionnaire (MPQ), Short-Form McGill Pain Questionnaire (SF-MPQ), Chronic Pain Grade Scale (CPGS), Short Form-36 Bodily Pain Scale (SF-36 BPS), and Measure of Intermittent and Constant Osteoarthritis Pain (ICOAP). *Arthritis Care Res (Hoboken)* 2011;63 Suppl 11:S240-52. doi: 10.1002/acr.20543
30. Pesonen A, Kauppila T, Tarkkila P, et al. Evaluation of easily applicable pain measurement tools for the assessment of pain in demented patients. *Acta anaesthesiologica Scandinavica* 2009;53(5):657-64. doi: 10.1111/j.1399-6576.2009.01942.x
31. Brunelli C, Zecca E, Martini C, et al. Comparison of numerical and verbal rating scales to measure pain exacerbations in patients with chronic cancer pain. *Health Qual Life Outcomes* 2010;8:42. doi: 10.1186/1477-7525-8-42 [published Online First: 2010/04/22]
32. McHugh ML. Interrater reliability: the kappa statistic. *Biochem Med (Zagreb)* 2012;22(3):276-82.
33. Ibanez B, James S, Agewall S, et al. 2017 ESC Guidelines for the management of acute myocardial infarction in patients presenting with ST-segment elevation: The Task Force for the management of acute myocardial infarction in patients presenting with ST-segment elevation of the European Society of Cardiology (ESC). *Eur Heart J* 2018;39(2):119-77. doi: 10.1093/eurheartj/ehx393
34. Collet JP, Thiele H, Barbato E, et al. 2020 ESC Guidelines for the management of acute coronary syndromes in patients presenting without persistent ST-segment elevation. *Eur Heart J* 2020 doi: 10.1093/eurheartj/ehaa575 [published Online First: 2020/08/29]
35. Widgren BR, Jourak M. Medical Emergency Triage and Treatment System (METTS): a new protocol in primary triage and secondary priority decision in emergency medicine. *J Emerg Med* 2011;40(6):623-8. doi: 10.1016/j.jemermed.2008.04.003 [published Online First: 2008/10/18]
36. Magnusson C, Herlitz J, Axelsson C. Patient characteristics, triage utilisation, level of care, and outcomes in an unselected adult patient population seen by the emergency medical services: a prospective observational study. *BMC Emerg Med* 2020;20(1):7. doi: 10.1186/s12873-020-0302-x [published Online First: 2020/01/30]
37. Roffi M, Patrono C, Collet JP, et al. 2015 ESC Guidelines for the management of acute coronary syndromes in patients presenting without persistent ST-segment elevation: Task Force for the Management of Acute Coronary Syndromes in Patients Presenting without Persistent ST-Segment Elevation of the European Society of Cardiology (ESC). *Eur Heart J* 2016;37(3):267-315. doi: 10.1093/eurheartj/ehv320 [published Online First: 2015/08/29]
38. Reuter PG, Pradeau C, Huo Yung Kai S, et al. Predicting acute coronary syndrome in males and females with chest pain who call an emergency medical communication

For peer review only - http://bmjopen.bmj.com/site/about/guidelines.xhtml

centre. *Scand J Trauma Resusc Emerg Med* 2019;27(1):92. doi: 10.1186/s13049-019-0670-y [published Online First: 2019/10/17]

39. van Dongen DN, Fokkert MJ, Tolsma RT, et al. Value of Prehospital Troponin Assessment in Suspected Non-ST-Elevation Acute Coronary Syndrome. *Am J Cardiol* 2018 doi: 10.1016/j.amjcard.2018.07.037 [published Online First: 2018/08/20]
40. Maurin Söderholm H, Andersson H, Andersson Hagiwara M, et al. Research challenges in prehospital care: the need for a simulation-based prehospital research laboratory. *Adv Simul (Lond)* 2019;4:3. doi: 10.1186/s41077-019-0090-0 [published Online First: 2019/02/13]
41. Dickert NW, Brown J, Cairns CB, et al. Confronting Ethical and Regulatory Challenges of Emergency Care Research With Conscious Patients. *Ann Emerg Med* 2016;67(4):538-45. doi: 10.1016/j.annemergmed.2015.10.026 [published Online First: 2015/12/18]
42. Asghar Z, Phung VH, Siriwardena AN. Ethnicity and pre-hospital care for people with suspected cardiac pain: cross-sectional study. *J Eval Clin Pract* 2016;22(5):721-5. doi: 10.1111/jep.12523 [published Online First: 2016/03/10]

For peer review only

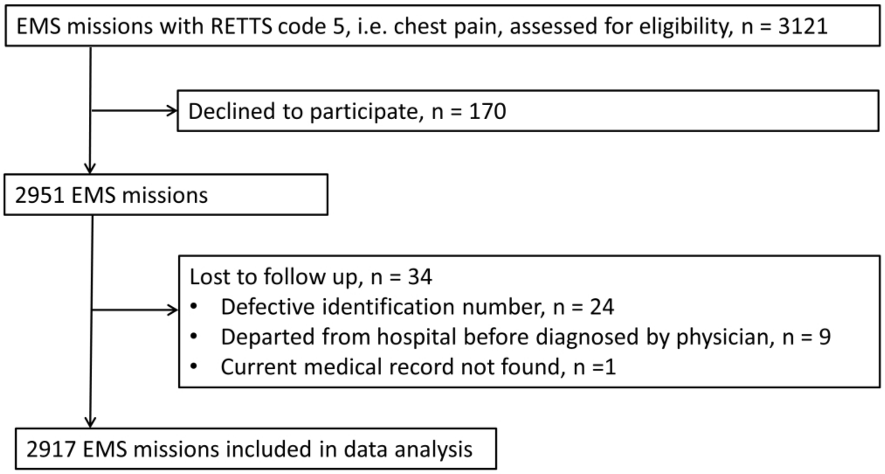

Figure 1 - Flow chart of inclusion process

254x190mm (300 x 300 DPI)

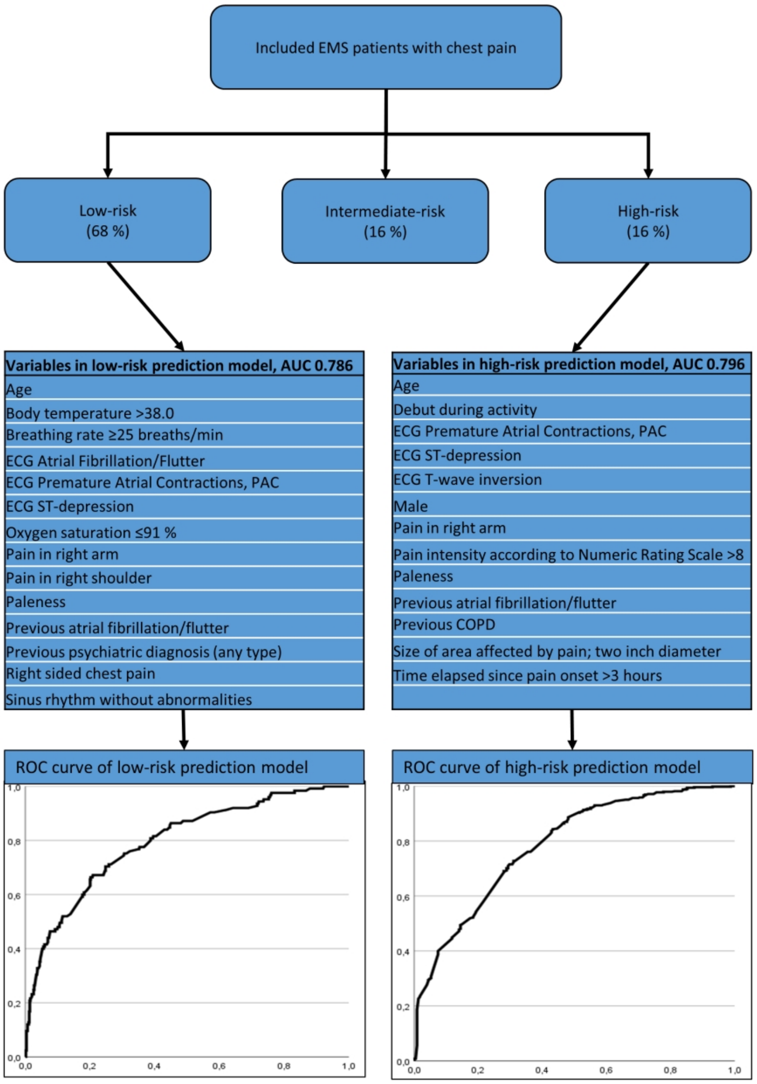

Figure 2 - Schematic presentation of prediction models accuracy

190x275mm (300 x 300 DPI)

Supplemental material 1 - Analog version of study questionnaire

1. Patient is (several options possible)?
□Pale □Clammy □Neither □ Can't tell

2. Patient is nauseas, is vomiting or has been vomiting (several options possible)?
□Nausea □Vomiting/has been vomiting □Neither □ Can't tell

3. The patient experiences his/her breathing as affected?
□Affected □Unaffected □ Can't tell

4. Pain intensity (when resting) on EMS arrival according to NRS (0-10, 0 = "no pain", 10 = "worst imaginable pain").
NRS: 0 1 2 3 4 5 6 7 8 9 10

If NRS is not possible to use, ask the patient to rate his/her pain as follows:
□No pain □Mild pain □Moderate pain □Severe pain □Unbearable pain

5. At what time and date did the pain, inducing the EMS contact, start?
Time: □ Can't tell
Date:

6. How did the pain start?
□Suddenly (seconds) □Quickly (minutes) □Slowly (hours) □ Can't tell

7. Did the pain start in conjunction with (several options possible)?
□Activity □While resting □In pain when waking up □ Can't tell

8. Development of pain (several options possible).
□Pain is constant □Pain come and go □Pain increases gradually □ Can't tell

9. How does the patient describe his/her pain (several options possible)?
Do the patient describe his/her pain as pressuring, cramping, burning, cutting, stabbing, dull, discomfort, swaying, bursting etc. Please write below.

10. Chest pain localisation (several options possible)?
□Central □Left side of chest □Right side of chest □Upper half of chest
□Lower half of chest □Entire chest □ Can't tell

11. Do the patient have pain somewhere else other than in their chest (several different areas/body parts can be specified).
Please write below which area/body part that hurts or if the chest pain is radiating somewhere. If no other pain than chest pain, write: "No other pain".

12. Size of area affected by pain?
□Size of a 5 sek coin □Size of a palm □Entire chest □ Can't tell

13. Chest pain affected by palpation?
□Yes □No □ Can't tell

14. Is pain affected by movement?
□Yes □No □ Can't tell

15. Is pain affected by breathing?
□Yes □No □ Can't tell

Supplemental material 2 - ECG interpretation manual

1. Heart rate: Provided by computer.
2. Sinus rhythm: Positive P wave in lead II with a constant morphology, rate between 50 and 100/min, constant PQ interval and normal QRS morphology.(2)
3. Sinus Bradycardia: Positive P-wave in lead II with a constant morphology, rate <50/min, constant PQ interval and normal QRS morphology.(2)
4. Sinus Tachycardia: Positive P wave in lead II with a constant morphology, rate >100/min, constant PQ interval and normal QRS morphology.(2)
5. Supraventricular Tachycardia: Fast and regular ventricular rhythm usually between 150-200/min. Episodic presentation with sudden onset. Normal QRS-complexes.(2)
6. Atrial Fibrillation: No evident P-waves instead the ECG reveals low-amplitude baseline oscillations (fibrillary or f waves from the fibrillating atria) and an irregular ventricular rhythm. Normal QRS complex duration during AV conduction.(2, 5)
7. Atrial Flutter: In typical atrial flutter, the ECG reveals identically recurring, regular, sawtooth flutter waves, and evidence of continual electrical activity (lack of an isoelectric interval between flutter waves), often best visualized in leads II, III, aVF, or V1. The atrial rate during typical atrial flutter is usually 250 to 350 beats/min with variations in the degree of ventricular conduction, usually 2:1 conduction. Normal QRS complex duration during AV conduction.(2, 5)
8. Atrial pacing: Pacing spike precedes the p wave. Morphology of p wave dependent of lead placement but may appear normal.
9. Ventricular pacing: Pacing spike precedes the QRS complex.
10. Second Degree AV block – II type 1: Progressive prolongation of the PR interval culminating in a non-conducted P wave. The PR interval is longest immediately before the dropped beat. The PR interval is shortest immediately after the dropped beat.(5)
11. Second Degree AV block – II type 2: Intermittent non-conducted P waves without progressive prolongation of the PR interval. The PR interval in the conducted beats remains constant. The P waves continue at a constant rate. The RR interval surrounding the dropped beat(s) is an exact multiple of the preceding RR interval.(5)
12. Third Degree AV block – III: Normal P waves with regular rhythm. Complete absence of AV conduction – none of the supraventricular impulses are conducted to the ventricles. Perfusing rhythm is maintained by a junctional or ventricular escape rhythm.(5)
13. Suspected STEMI: ST-elevation of the J-point in two contiguous leads with ≥ 2.5 mm in men < 40 years, ≥ 2 mm in men > 40 years, or ≥ 1,5 mm in women in V2-V3 and/or ≥ 1 mm in the other leads.(1)
14. ST-Depression: ST-depression ≥ 0.5 mm in at least two contiguous leads. (3)
15. T-wave inversion: Negative electrical axis of the T wave. Positive T wave is always expected at least in the precordial leads V2-V6 and in AVL where the R wave is at least 5mm, deviation from this rule is pathologic.
16. Q-wave: Pathologic q-wave ≥ 25% of R-wave or duration of ≥0,03 s in two contiguous leads. Q-wave or QS-komplex in V2 or V3 is always pathologic. (2)
17. QRS-duration: Provided by computer.
18. Premature Ventricular Complex-PVC: Characterized by the premature occurrence of a QRS complex that is abnormal in shape and has a duration usually exceeding the dominant QRS complex, generally longer than 120 milliseconds. The T wave is usually large and opposite in direction to the major deflection of the QRS. The QRS complex is not preceded by a premature P wave but can be preceded by a nonconducted sinus P wave occurring at its expected time.(5)
19. PAC-SVES: Premature Atrial Complexes: Premature beat with or without a premature P wave that generally differs from a normal sinus P wave. Normal QRS complex. (2,5)

For peer review only - http://bmjopen.bmj.com/site/about/guidelines.xhtml

1. Left Bundle Branch Block – LBBB: QRS duration of ≥ 120 ms. Dominant S wave in V1-V3. Broad M-shaped QRS complexes in lateral leads (V5-V6), ST and T waves usually opposite in direction to QRS. Absence of Q waves in lateral leads (I, V5-V6; small Q waves are still allowed in aVL). Prolonged R wave peak time > 60ms in left precordial leads (V5-6).(5,6)
2. Right Bundle Branch Block – RBBB: QRS duration ≥ 120 msec. rsr′, rsR′, or rSR′, patterns in leads V1 and V2. S waves in leads I and V6 ≥ 40 msec wide. Normal time to peak R wave in leads V5 and V6 but > 50 msec in V1.(5)
3. Ventricular Tachycardia: VT is suggested by the occurrence of a series of three or more consecutive, abnormally shaped QRS complexes longer than 120 milliseconds, with the ST-T vector pointing opposite the major QRS deflection. The R-R interval can be regular or varying. Atrial activity can be independent of ventricular activity (AV dissociation), or the atria can be depolarized retrogradely (ventriculoatrial [VA] association). Depending on the particular type of VT, rates range from 70 to 250 beats/min, usually > 100 beats/min.(2,5)
4. Uninterpretable ECG: Meaningful ECG interpretation not possible due to low ECG quality as judged by interpreter.

References

1. Ibanez B, James S, Agewall S, Antunes MJ, Bucciarelli-Ducci C, Bueno H, et al. 2017 ESC Guidelines for the management of acute myocardial infarction in patients presenting with ST-segment elevationThe Task Force for the management of acute myocardial infarction in patients presenting with ST-segment elevation of the European Society. Eur Heart J. 2018 Jan 7;39(2):119–77.
2. Jern Sverker. Klinisk EKG-Diagnostik. 2010;
3. Roffi M, Patrono C, Collet J-P, Mueller C, Valgimigli M, Andreotti F, et al. 2015 ESC Guidelines for the management of acute coronary syndromes in patients presenting without persistent ST-segment elevationTask Force for the Management of Acute Coronary Syndromes in Patients Presenting without Persistent ST-Segment Elevation of th. Eur Heart J. 2016 Jan 14;37(3):267–315.
4. Rhinehardt J, Brady WJ, Perron AD, Mattu A. Electrocardiographic manifestations of Wellens' syndrome. Am J Emerg Med. 2002 Nov 1;20(7):638–43.
5. Douglas P. Zipes, Peter Libby, Robert O. Bonow, Douglas L. Mann, Gordon F. Tomaselli and EB. Braunwald's Heart Disease: A Textbook of Cardiovascular Medicine 11th edition. 2019;
6. Surawicz B, Childers R, Deal BJ, Gettes LS. AHA/ACCF/HRS Recommendations for the Standardization and Interpretation of the Electrocardiogram. Part III: Intraventricular Conduction Disturbances A Scientific Statement From the American Heart Association Electrocardiography and Arrhythmias Committee, . J Am Coll Cardiol. 2009 Mar 17;53(11):976–81.

Supplemental material 3 - Description of study sample

		Sex		Age group		
	All n (%)	Men n (%)	Women n (%)	≤50 n (%)	51-64 n (%)	≥65 n (%)
	2917 (100)	1465 (50.2)	1452 (49.8)	476 (16.3)	539 (18.5)	1902 (65.2)
Mean age (SD)	69 (17)	67 (18)	70 (17)	39 (9)	58 (4)	79 (8)
Median age (Q1-Q3)	72 (58-82)	70 (56-80)	73 (60-83)	41 (31-47)	58 (55-62)	79 (73-85)
Priority by EMD[a]						
Priority 1	1836 (62.9)	936 (63.9)	900 (62.0)	280 (55.5)	232 (59.9)	1233 (64.8)
Priority 2	1049 (36.0)	513 (35)	536 (36.9)	189 (39.7)	208 (38.6)	652 (34.3)
Priority 3	32 (1.1)	16 (1.1)	16 (1.1)	7 (1.5)	8 (1.5)	17 (0.9)
Priority by EMS-personnel[b]						
Priority 1	311 (10.7)	208 (14.2)	103 (7.1)	37 (7.8)	68 (12.3)	206 (10.8)
Priority 2	1969 (67.5)	981 (67.0)	988 (68)	286 (60.1)	355 (65.9)	1328 (69.8)
Priority 3	325 (11.1)	139 (9.5)	186 (12.8)	68 (14.3)	63 (11.7)	194 (10.2)
Priority according to RETTS[c] by EMS-personnel[b]						
Red	290 (9.9)	187 (12.8)	103 (7.1)	32 (6.7)	59 (10.9)	199 (10.5)
Orange	1628 (55.8)	819 (55.9)	809 (55.7)	239 (50.2)	291 (54.0)	1098 (57.7)
Yellow	887 (30.4)	419 (28.6)	468 (32.2)	170 (35.7)	170 (31.5)	547 (28.8)
Green	112 (3.8)	40 (2.7)	72 (5.0)	35 (7.4)	19 (3.5)	58 (3.0)
Transport from primary care centre	508 (17.4)	286 (19.5)	222 (15.3)	77 (16.2)	146 (27.1)	285 (15.0)
Transport to primary care centre	15 (0.5)	8 (0.5)	7 (0.5)	3 (0.6)	0 (0.0)	12 (0.6)
Transported to hospital	2600 (89.1)	1324 (90.4)	1276 (87.9)	388 (81.5)	488 (90.5)	1724 (90.6)
Admitted to hospital (missing = 1)	1409 (48.3)	812 (55.4)	597 (41.1)	123 (25.8)	235 (43.6)	1051 (55.3)
Did not convey	302 (10.4)	133 (9.1)	169 (11.6)	85 (17.9)	51 (9.5)	166 (8.7)
EMS response time (minutes)						
Median EMS dispatch to scene arrival (Q1-Q3)	10 (6-15)	9 (6-15)	10 (6-16)	10 (7-16)	10 (6-17)	9 (6-15)
Median EMS time at scene (Q1-Q3)	25 (18-31)	24 (18-30)	25 (19-32)	22 (16-27)	22 (15-27)	26 20-33)
Median transportation time (Q1-Q3)	25 (13-34)	25 (14-33)	25 (12-35)	25 (13-35)	24 (13-32)	25 13-34)
Median total EMS time (Q1-Q3)	95 (73-118)	95 (73-118)	95 (73-118)	89 (67-115)	92 (70-113)	97 75-120)

[a]Emergency dispatch centre

[b]Emergency medical services

[c]Rapid Emergency Triage and Treatment System

For peer review only

Supplemental material 4 - Prediction of high-risk conditions, univariate analyses

		Total with variable % (n)	Patients with high-risk condition % (n)	Patients without high-risk condition % (n)	High-risk vs without high-risk, p-value[a]	Odds Ratio	Confidence Interval, 95 %
All		2917 (100)	16.0 (467)	84.0 (2450)			
	Men	50.2 (1465)	63.8 (298)	47.6 (1167)	<0.001***	1.93	1.58-2.38
	Women	49.8 (1452)	36.2 (169)	52.4 (1283)			
Age		72 (median)	75 (median)	71 (median)	<0.001***	1.02	1.01-1.03
Age-group							
	Age ≤50	16.3 (476)	6.2 (29)	18.2 (447)			
	Age 51-64	18.5 (539)	17.1 (80)	18.7 (459)	<0.001***	2.68	1.72-4.19
	Age ≥65	65.2 (1902)	76.7 (358)	63.0 (1544)	<0.001***	3.57	2.41-5.29
Previous history of the following according to medical record							
Acute coronary syndrome (AMI, unstable angina)		29.3 (856)	27.8 (130)	29.6 (726)	0.435	0.91	0.74-1.14
Chronic obstructive pulmonary disease		10.5 (307)	7.5 (35)	11.2 (272)	0.021*	0.64	0.45-0.94
Angina pectoris		20.0 (582)	16.9 (79)	20.5 (503)	0.074	0.78	0.61-1.02
Hypertension		58.1 (1696)	63.2 (295)	57.2 (1401)	0.016*	1.28	1.05-1.58
Heart failure		20.6 (600)	18.0 (84)	21.1 (516)	0.132	0.82	0.64-1.06
Diabetes mellitus (type I and II)		19.8 (578)	23.6 (110)	19.1 (468)	0.027*	1.30	1.03-1.65
Stroke		8.2 (240)	7.9 (37)	8.3 (203)	0.794	0.95	0.66-1.37
Kidney disease		12.0 (350)	12.6 (59)	11.9 (291)	0.645	1.07	0.80-1.45
Atrial fibrillation/flutter		26.3 (767)	15.8 (74)	28.3 (693)	<0.001***	0.47	0.37-0.62
Rheumatism		2.3 (66)	1.5 (7)	2.4 (59)	0.230	0.61	0.28-1.36
Cancer (any type)		17.2 (502)	18.6 (87)	16.9 (415)	0.375	1.12	0.87-1.45
Psychiatric diagnosis (any type)		38.9 (1136)	27.4 (128)	41.1 (1008)	<0.001***	0.54	0.43-0.67

For peer review only

Supplemental material 4 (continues) - Prediction of high-risk conditions, univariate analyses

	Total with variable % (n)	Patients with high-risk condition % (n)	Patients without high-risk condition % (n)	High-risk vs without high-risk, p-value[a]	Odds Ratio	Confidence Interval, 95 %
Vital signs on EMS arrival (missing)						
Breathing rate ≤8 breaths/min (22)	0 (0)	0 (0)	0 (0)	–	–	–
Breathing rate ≥25 breaths/min (22)	10.2 (297)	14.7 (68)	9.4 (229)	0.001**	1.65	1.24-2.22
Oxygen saturation ≤91 % (8)	3.8 (110)	6.4 (30)	3.3 (80)	0.001**	2.02	1.32-3.12
Heart rate ≤40 beats/min (18)	0.2 (7)	0.9 (4)	0.1 (3)	–	–	–
Heart rate ≥131 beats/min (18)	3.1 (91)	2.4 (11)	3.3 (80)	0.295	0.71	0.38-1.35
Systolic blood pressure ≤90 mmHg (22)	1.0 (29)	1.5 (7)	0.9 (22)	0.230	1.69	0.72-3.98
Systolic blood pressure ≥220 mmHg (22)	1.0 (28)	0.4 (2)	1.1 (26)	–	–	–
Decreased level of consciousness or new confusion (68)	0.9 (25)	1.5 (7)	0.7 (18)	0.114	2.03	0.84-4.89
Body temperature ≤35.0 (110)	0.2 (6)	0.5 (2)	0.2 (4)	–	–	–
Body temperature >38.0 (110)	2.3 (67)	2.0 (9)	2.5 (58)	0.599	0.82	0.41-1.68
Symptoms on EMS arrival (missing)						
Pale (565)	16.4 (386)	29.1 (111)	14.0 (275)	<0.001***	2.52	1.96-3.26
Clammy (565)	8.7 (204)	14.4 (55)	7.6 (149)	<0.001***	2.05	1.48-2.86
Nausea (576)	27.1 (635)	26.8 (101)	27.2 (534)	0.873	0.98	0.76-1.26
Vomiting (576)	7.0 (165)	7.4 (28)	7.0 (137)	0.754	1.07	0.70-1.63
Affected breathing according to patient (596)	44.6 (1040)	41.9 (158)	45.1 (882)	0.248	0.87	0.70-1.10
Pain intensity according to Numeric Rating Scale. NRS 0-10 (415)						
0	20.3 (509)	17.4 (71)	20.9 (438)	<0.001***		
1	3.8 (94)	3.2 (13)	3.9 (81)	0.976	0.99	0.52-1.87
2	11.5 (287)	9.8 (40)	11.8 (247)	0.996	0.99	0.66-1.52
3	7.6 (189)	8.6 (35)	7.4 (154)	0.136	1.40	0.90-2.19
4	8.1 (202)	6.8 (28)	8.3 (174)	0.976	0.99	0.62-1.59
5	16.7 (418)	15.4 (63)	17.0 (355)	0.629	1.09	0.76-1.58
6	8.3 (207)	6.8 (28)	8.6 (179)	0.882	0.96	0.60-1.56
7	10.5 (263)	12.2 (50)	10.2 (213)	0.068	1.44	0.97-2.15
8	8.2 (206)	9.8 (40)	7.9 (166)	0.069	1.48	0.97-2.28
9	2.6 (64)	4.9 (20)	2.1 (44)	0.001**	2.80	1.56-5.03
10	2.5 (63)	5.1 (21)	2.4 (42)	<0.001***	3.08	1.73-5.51

For peer review only

Supplemental material 4 (continues) - Prediction of high-risk conditions, univariate analyses

	Total with variable % (n)	Patients with high-risk condition % (n)	Patients without high-risk condition % (n)	High-risk vs without high-risk, p-value[a]	Odds Ratio	Confidence Interval, 95 %
Time elapsed since pain onset >3 hours (1007)	45.2 (863)	40.3 (128)	46.2 (735)	0.053	0.78	0.62-1.00
Debut						
Debut during activity (752)	22.1 (479)	32.8 (116)	20.0 (363)	<0.001***	1.94	1.51-2.50
Debut while resting (752)	65.5 (1419)	59.6 (211)	66.7 (1208)	0.010*	0.73	0.58-0.93
Debut while sleeping (752)	15.8 (342)	11.3 (40)	16.7 (302)	0.012*	0.63	0.45-0.91
Sudden debut, within seconds (875)	35.7 (729)	33.0 (115)	36.3 (614)	0.239	0.86	0.68-1.10
Quick debut, within minutes (875)	35.2 (718)	42.7 (149)	33.6 (569)	0.001**	1.47	1.16-1.86
Slow debut, within hours (875)	29.1 (595)	24.4 (85)	30.1 (510)	0.031*	0.74	0.57-0.97
Constant pain (732)	55.5 (1212)	61.8 (235)	49.9 (977)	<0.001***	1.66	1.31-2.11
Fluctuating pain (732)	40.4 (883)	30.5 (116)	39.2 (767)	0.001**	0.66	0.52-0.84
Pain aggravating over time (732)	10.8 (237)	8.7 (33)	10.4 (204)	0.279	0.80	0.55-1.19
Pain in other parts of the body (1197)						
Head	2.5 (43)	0.7 (2)	2.9 (41)	–	–	–
Throat	10.3 (177)	8.2 (24)	10.7 (153)	0.202	0.74	0.48-1.17
Jaw	5.3 (92)	7.5 (22)	4.9 (70)	0.071	1.58	0.96-2.58
Neck	2.5 (43)	2.7 (8)	2.5 (35)	0.773	1.12	0.56-2.44
Between scapulars	2.2 (37)	4.1 (12)	1.8 (25)	0.014*	2.40	1.19-4.84
Back	15.2 (261)	15.4 (45)	15.1 (216)	0.902	1.02	0.72-1.45
Left shoulder	8.5 (147)	7.9 (23)	8.7 (124)	0.653	0.89	0.57-1.43
Right shoulder	4.2 (72)	6.2 (18)	3.8 (54)	0.067	1.67	0.97-2.89
Left arm	24.0 (412)	33.6 (98)	22.0 (314)	<0.001***	1.79	1.36-2.36
Right arm	8.4 (145)	16.4 (48)	6.8 (97)	<0.001***	2.69	1.86-3.92
Left hand	1.0 (17)	0.7 (2)	1.1 (15)	–	–	–
Right hand	0.3 (6)	0.3 (1)	0.4 (5)	–	–	–
Stomach	7.0 (121)	4.8 (14)	7.5 (107)	0.103	0.62	0.35-1.10
Left leg	1.7 (29)	0.7 (2)	1.9 (27)	–	–	–
Right leg	1.4 (24)	0.0 (0)	1.7 (24)	–	–	–
No other pain	39.3 (676)	36.3 (106)	39.9 (570)	0.249	0.85	0.66-1.11

Supplemental material 4 (continues) - Prediction of high-risk conditions, univariate analyses

	Total with variable % (n)	Patients with high-risk condition % (n)	Patients without high-risk condition % (n)	High-risk vs without high-risk, p-value[a]	Odds Ratio	Confidence Interval, 95 %
Pain quality (1175)						
Band-shaped	3.3 (58)	5.2 (15)	3.0 (43)	0.051	1.81	1.00-3.32
Burning	4.4 (76)	4.5 (13)	4.3 (63)	0.869	1.05	0.57-1.94
Stabbing	9.7 (169)	4.2 (12)	10.8 (157)	0.001**	0.36	0.20-0.66
Cramping	8.7 (151)	9.8 (28)	8.4 (123)	0.461	1.17	0.76-1.81
Dull pain	13.9 (242)	13.3 (38)	14.0 (204)	0.746	0.94	0.65-1.36
Fells like something is on the chest	0.7 (12)	1.0 (3)	0.6 (9)	–	–	–
Discomfort	10.2 (178)	8.7 (25)	10.5 (153)	0.368	0.81	0.52-1.27
Tingling/Stinging	5.7 (99)	1.4(4)	6.5 (95)	–	–	–
Swaying	1.7 (30)	1.4 (4)	1.8 (26)	–	–	–
Pressuring	57.9 (1008)	63.6 (182)	56.7 (826)	0.031*	1.33	1.03-1.74
Heaviness	1.0 (17)	1.4 (4)	0.9 (13)	–	–	–
Aching	2.4 (41)	3.1 (9)	2.2 (32)	0.336	1.44	0.68-3.06
Chest pain localization (640)						
Central pain	53.4 (1215)	64.5 (240)	51.2 (975)	<0.001***	1.73	1.38-2.18
Left side of chest	35.5 (809)	28.0 (104)	37.0 (705)	0.001**	0.66	0.52-0.84
Right side of chest	5.1 (116)	2.4 (9)	5.6 (107)	0.013*	0.41	0.21-0.83
Upper part of chest	6.7 (152)	7.3 (27)	6.6 (125)	0.623	1.11	0.72-1.72
Lower part of chest	9.0 (204)	5.9 (22)	9.6 (182)	0.026*	0.59	0.38-0.94
All over the chest	11.8 (269)	12.6 (47)	11.7 (222)	0.592	1.09	0.78-1.54
Size of area affected by pain (794)						
Two inch diameter	10.7 (228)	5.1 (18)	11.9 (210)	<0.001***	0.39	0.24-0.65
Size of patient's palm	58.4 (1240)	61.0 (216)	57.9 (1024)	0.275	1.13	0.90-1.44
Entire chest	30.9 (655)	33.9 (120)	30.2 (535)	0.174	1.18	0.93-1.51
Palpation tenderness (655)	22.3 (505)	15.8 (58)	23.6 (447)	0.001**	0.60	0.45-0.82
Pain affected by movement (719)	17.0 (373)	11.5 (41)	18.0 (332)	0.003**	0.59	0.42-0.83
Pain affected by breathing (692)	25.8 (573)	17.3 (63)	27.4 (510)	<0.001***	0.55	0.41-0.74

For peer review only

Supplemental material 4 (continues) - Prediction of high-risk conditions, univariate analyses

	Total with variable % (n)	Patients with high-risk condition % (n)	Patients without high-risk condition % (n)	High-risk vs without high-risk, p-value[a]	Odds Ratio	Confidence Interval, 95 %
Troponin T (missing)						
High-sensitive Troponin T, cut-off >14 ng/L (1416)	12 (median)	33 (median)	11 (median)	<0.001***	1.01	1.01-1.02
Troponin T adjusted to Roche Cobas h232 (1416)						
Tnt <50 ng/L	88.1 (1323)	62.9 (161)	93.3 (1162)	<0.001***		
Tnt 51-100 ng/L	6.6 (99)	15.6 (40)	4.7 (59)	<0.001***	4.89	3.17-7.55
Tnt 101-1000 ng/L	4.5 (67)	17.2 (44)	1.8 (23)	<0.001***	13.8	8.12-23.47
Tnt >1000 ng/L	0.8 (12)	4.3 (11)	0.1 (1)	–	–	–
ECG (missing[b])						
ECG transmitted to hospital	95.5 (2785)	97.2 (454)	95.1 (2331)	0.051	1.78	1.00-3.19
Sinus Rhythm, SR (235)	83.8 (2248)	84.7 (377)	83.6 (1871)	0.572	1.08	0.82-1.44
Sinus Bradycardia (235)	1.3 (34)	1.1 (5)	1.3 (29)	0.766	0.86	0.33-2.25
Sinus Tachycardia (235)	8.9 (240)	12.4 (55)	8.3 (185)	0.006	1.56	1.14-2.15
Supraventricular Tachycardia, SVT (235)	0.5 (14)	0.2 (1)	0.6 (3)	–	–	–
Atrial Fibrillation/Flutter, AF (235)	14.3 (383)	13.9 (62)	14.3 (321)	0.818	0.96	0.72-1.30
Ventricular Tachycardia, VT (235)	0.0 (1)	0.0 (0)	0.2 (1)	–	–	–
Atrial Pacing (235)	1.0 (29)	0.4 (2)	1.2 (27)	–	–	–
Ventricular Pacing (150)	3.1 (85)	1.8 (8)	3.3 (77)	0.083	0.52	0.25-1.09
AV-block II type 1 (235)	0.0 (1)	0.2 (1)	0.0 (0)	–	–	–
AV-block II type 2 (235)	0.0 (1)	0.0 (0)	0.0 (1)	–	–	–
AV-Block III (235)	0.1 (2)	0.4 (2)	0.0 (0)	–	–	–
ST-Elevation (235)	6.1 (164)	22.9 (102)	2.8 (62)	<0.001***	10.4	7.46-14.59
ST-Depression (235)	7.6 (204)	23.8 (106)	4.4 (98)	<0.001***	5.82	5.07-9.19
T-wave Inversion (235)	14.5 (388)	22.7 (101)	12.8 (287)	<0.001***	1.99	1.55-2.57
Q-wave (235)	4.8 (130)	6.5 (29)	4.5 (101)	0.074	1.47	0.96-2.26
Premature Ventricular Contraction, PVC (235)	5.5 (147)	7.9 (35)	5.0 (112)	0.016*	1.62	1.09-2.40
Premature Atrial Contractions, PAC (235)	3.5 (94)	6.1 (27)	3.0 (67)	0.002**	2.09	1.32-3.31
Left Bundle Branch Block, LBBB (235)	6.3 (168)	6.5 (29)	6.2 (139)	0.810	1.05	0.70-1.59
Right Bundle Branch Block, RBBB (235)	6.3 (170)	9.2 (41)	5.8 (129)	0.007**	1.65	1.15-2.39
Sinus rhythm and none of abnormalities above (150)	44.6 (1233)	26.5 (120)	48.1 (1113)	<0.001***	0.38	0.31-0.49
QRS-duration (150)	92 (median)	96 (median)	92 (median)	<0.001***	1.00	1.01-1.01
Uninterpretable ECG (132)	0.6 (18)	0.2 (1)	0.7 (17)	–	–	–

For peer review only

Supplemental material 4 (continues) - Prediction of high-risk conditions, univariate analyses

*$p < 0.05$

**$p < 0.01$

***$p < 0.001$

[a]Logistic regression

[b]When ventricular pacing or uninterpretable remaining ECG interpretation has been treated as missing.

For peer review only

Supplemental material 5 - Prediction of low-risk conditions, univariate analyses

		Total with variable % (n)	Patients with low-risk condition % (n)	Patients without low-risk condition % (n)	Low-risk vs without low-risk, p-value[a]	Odds Ratio	Confidence Interval, 95 %
All		2917 (100)	68.4 (1995)	31.6 (922)			
	Men	50.2 (1465)	46.2 (922)	41.1 (379)	<0.001***	0.60	0.51-0.70
	Women	49.8 (1452)	53.8 (1073)	58.9 (543)			
Age		72 (median)	70 (median)	76 (median)	<0.001***	0.98	0.97-0.98
Age-group							
	Age ≤50	16.3 (476)	20.4 (406)	7.6 (70)			
	Age 51-64	18.5 (539)	20.1 (401)	15.0 (138)	<0.001***	0.50	0.36-0.69
	Age ≥65	65.2 (1902)	59.5 (1188)	77.4 (714)	<0.001***	0.29	0.22-0.38
Previous history of the following according to medical record							
Acute coronary syndrome (AMI, unstable angina)		29.3 (856)	29.0 (578)	30.2 (278)	0.515	0.95	0.80-1.12
Chronic obstructive pulmonary disease		10.5 (307)	9.7 (194)	12.3 (113)	0.039*	0.77	0.60-0.99
Angina pectoris		20.0 (582)	20.5 (409)	18.8 (173)	0.275	1.12	0.92-1.36
Hypertension		58.1 (1696)	55.6 (1110)	63.6 (586)	<0.001***	0.72	0.61-0.84
Heart failure		20.6 (600)	18.6 (371)	24.8 (229)	<0.001***	0.69	0.57-0.83
Diabetes mellitus (type I and II)		19.8 (578)	19.4 (388)	20.6 (190)	0.465	0.93	0.77-1.13
Stroke		8.2 (240)	7.5 (150)	9.8 (90)	0.041*	0.75	0.57-0.99
Kidney disease		12.0 (350)	11.0 (219)	14.2 (131)	0.013*	0.75	0.59-0.94
Atrial fibrillation/flutter		26.3 (767)	25.0 (498)	29.2 (269)	0.016*	0.81	0.68-0.96
Rheumatism		2.3 (66)	2.3 (46)	2.2 (20)	0.818	1.06	0.63-1.81
Cancer (any type)		17.2 (502)	15.2 (303)	21.6 (199)	<0.001***	0.65	0.53-0.79
Psychiatric diagnosis (any type)		38.9 (1136)	42.5 (848)	31.2 (288)	<0.001***	1.63	1.38-1.92

For peer review only

Supplemental material 5 (continues) - Prediction of low-risk conditions, univariate analyses

	Total with variable % (n)	Patients with low-risk condition % (n)	Patients without low-risk condition % (n)	Low-risk vs without low-risk, p-value[a]	Odds Ratio	Confidence Interval, 95 %
Vital signs on EMS arrival (missing)						
Breathing rate ≤8 breaths/min (22)	0 (0)	0 (0)	0 (0)	–	–	–
Breathing rate ≥25 breaths/min (22)	10.2 (297)	6.2 (122)	19.1 (175)	<0.001***	0.28	0.22-0.36
Oxygen saturation ≤91 % (8)	3.8 (110)	1.2 (23)	9.4 (87)	<0.001***	0.11	0.07-0.18
Heart rate ≤40 beats/min (18)	0.2 (7)	0.1 (1)	0.7 (6)	–	–	–
Heart rate ≥131 beats/min (18)	3.1 (91)	0.9 (18)	7.9 (73)	<0.001***	0.11	0.06-0.18
Systolic blood pressure ≤90 mmHg (22)	1.0 (29)	0.6 (12)	1.9 (17)	0.003**	0.32	0.15-0.67
Systolic blood pressure ≥220 mmHg (22)	1.0 (28)	0.8 (16)	1.3 (12)	0.198	0.61	0.29-1.30
Decreased level of consciousness or new confusion (68)	0.9 (25)	0.5 (9)	1.8 (16)	0.001**	0.26	0.12-0.60
Body temperature ≤35.0 (110)	0.2 (6)	0.2 (3)	0.3 (3)	–	–	–
Body temperature >38.0 (110)	2.3 (67)	1.4 (27)	4.5 (40)	<0.001***	0.30	0.18-0.49
Symptoms on EMS arrival (missing)						
Pale (565)	16.4 (386)	11.5 (182)	26.6 (204)	<0.001***	0.36	0.29-0.45
Clammy (565)	8.7 (204)	6.1 (97)	14.0 (107)	<0.001***	0.40	0.30-0.54
Nausea (576)	27.1 (635)	26.6 (421)	28.2 (214)	0.405	0.92	0.76-1.12
Vomiting (576)	7.0 (165)	5.8 (92)	9.6 (73)	0.001**	0.58	0.42-0.80
Affected breathing according to patient (596)	44.6 (1040)	40.7 (640)	52.6 (400)	<0.001***	0.62	0.52-0.74
Pain intensity according to Numeric Rating Scale. NRS 0-10 (415)						
0	20.3 (509)	21.8 (367)	17.4 (142)	0,004*		
1	3.8 (94)	4.3 (72)	2.7 (22)	0.369	1.27	0.76-2.12
2	11.5 (287)	11.9 (200)	10.7 (87)	0.470	0.89	0.65-1.22
3	7.6 (189)	7.3 (123)	8.1 (66)	0.072	0.72	0.51-1.03
4	8.1 (202)	8.7 (147)	6.7 (55)	0.857	1.03	0.72-1.49
5	16.7 (418)	16.1 (272)	17.9 (146)	0.022*	0.72	0.55-0.95
6	8.3 (207)	8.3 (140)	8.2 (67)	0.233	0.81	0.57-1.15
7	10.5 (263)	10.0 (169)	11.5 (94)	0.025*	0.70	0.51-0.96
8	8.2 (206)	7.5 (126)	9.8 (80)	0.004**	0.61	0.43-0.86
9	2.6 (64)	2.2 (37)	3.3 (27)	0.020*	0.53	0.31-0.90
10	2.5 (63)	2.0 (34)	3.6 (29)	0.004**	0.45	0.27-0.77

For peer review only

Supplemental material 5 (continues) - Prediction of low-risk conditions, univariate analyses

	Total with variable % (n)	Patients with low-risk condition % (n)	Patients without low-risk condition % (n)	Low-risk vs without low-risk, p-value[a]	Odds Ratio	Confidence Interval, 95 %
Time elapsed since pain onset >3 hours (1007)	45.2 (863)	45.9 (592)	43.8 (271)	0.394	1.09	0.90-1.32
Debut						
Debut during activity (752)	22.1 (479)	21.0 (306)	24.4 (173)	0.071	0.82	0.67-1.02
Debut while resting (752)	65.5 (1419)	65.6 (956)	65.4 (463)	0.920	1.01	0.84-1.22
Debut while sleeping (752)	15.8 (342)	16.9 (246)	13.6 (96)	0.047*	1.30	1.00-1.67
Sudden debut, within seconds (875)	35.7 (729)	37.5 (513)	32.1 (216)	0.017*	1.27	1.04-1.54
Quick debut, within minutes (875)	35.2 (718)	33.3 (456)	38.9 (262)	0.012*	0.78	0.65-0.95
Slow debut, within hours (875)	29.1 (595)	29.2 (400)	29.0 (195)	0.909	1.01	0.83-1.24
Constant pain (732)	55.5 (1212)	52.9 (778)	60.9 (434)	<0.001***	0.72	0.60-0.86
Fluctuating pain (732)	40.4 (883)	42.7 (629)	35.6 (254)	0.002**	1.35	1.12-1.62
Pain aggravating over time (732)	10.8 (237)	11.0 (162)	10.5 (75)	0.732	1.05	0.79-1.41
Pain in other parts of the body (1197)						
Head	2.5 (43)	3.0 (35)	1.4 (8)	0.051	2.16	0.10-4.70
Throat	10.3 (177)	10.5 (121)	9.9 (56)	0.743	1.06	0.76-1.48
Jaw	5.3 (92)	5.0 (58)	6.0 (34)	0.375	0.82	0.53-1.27
Neck	2.5 (43)	2.2 (26)	3.0 (17)	0.337	0.74	0.40-1.37
Between scapulars	2.2 (37)	1.9 (22)	2.7 (15)	0.308	0.71	0.36-1.38
Back	15.2 (261)	15.5 (179)	14.6 (82)	0.623	1.07	0.81-1.43
Left shoulder	8.5 (147)	8.6 (99)	8.5 (48)	0.983	1.00	0.70-1.44
Right shoulder	4.2 (72)	3.5 (41)	5.5 (31)	0.580	0.63	0.39-1.02
Left arm	24.0 (412)	22.9 (265)	26.1 (147)	0.144	0.84	0.67-1.06
Right arm	8.4 (145)	6.2 (72)	13.0 (73)	<0.001***	0.45	0.32-0.63
Left hand	1.0 (17)	1.2 (14)	0.5 (3)	–	–	–
Right hand	0.3 (6)	0.3 (4)	0.4 (2)	–	–	–
Stomach	7.0 (121)	6.9 (80)	7.3 (41)	0.779	0.95	0.54-1.40
Left leg	1.7 (29)	2.0 (23)	1.1 (6)	0.170	1.88	0.76-4.65
Right leg	1.4 (24)	1.8 (21)	0.5 (3)	–	–	–
No other pain	39.3 (676)	39.8 (461)	38.2 (215)	0.509	1.07	0.87-1.32

Supplemental material 5 (continues) - Prediction of low-risk conditions, univariate analyses

		Total with variable % (n)	Patients with low-risk condition % (n)	Patients without low-risk condition % (n)	Low-risk vs without low-risk, p-value[a]	Odds Ratio	Confidence Interval, 95 %
Pain quality (1175)							
	Band-shaped	3.3 (58)	3.0 (35)	4.0 (23)	0.250	0.73	0.43-1.25
	Burning	4.4 (76)	4.8 (56)	3.5 (20)	0.229	1.38	0.82-2.32
	Stabbing	9.7 (169)	10.9 (128)	7.2 (41)	0.015*	1.58	1.09-2.28
	Cramping	8.7 (151)	9.0 (1255)	8.1 (46)	0.547	1.12	0.78-1.61
	Dull pain	13.9 (242)	13.8 (162)	14.1 (80)	0.888	0.98	0.73-1.31
	Fells like something is on the chest	0.7 (12)	0.6 (7)	0.9 (5)	0.507	0.68	0.21-2.14
	Discomfort	10.2 (178)	10.7 (125)	9.3 (53)	0.386	1.16	0.83-1.63
	Tingling/Stinging	5.7 (99)	7.1 (83)	2.8 (16)	<0.001***	2.63	1.53-4.54
	Swaying	1.7 (30)	1.8 (21)	1.6 (9)	0.754	1.13	0.52-2.49
	Pressuring	57.9 (1008)	55.8 (654)	62.2 (354)	0.011*	0.77	0.62-0.94
	Heaviness	1.0 (17)	0.9 (11)	1.1 (6)	0.816	0.89	0.33-2.41
	Aching	2.4 (41)	2.0 (24)	3.0 (17)	0.227	0.69	0.36-1.27
Chest pain localization (640)							
	Central pain	53.4 (1215)	50.4 (776)	59.5 (439)	<0.001***	0.69	0.58-0.83
	Left side of chest	35.5 (809)	39.3 (605)	27.6 (204)	<0.001***	1.70	1.40-2.05
	Right side of chest	5.1 (116)	5.7 (87)	3.9 (29)	0.082	1.47	0.95-2.25
	Upper part of chest	6.7 (152)	6.8 (104)	6.5 (48)	0.821	1.01	0.93-1.10
	Lower part of chest	9.0 (204)	9.0 (138)	8.9 (66)	0.985	1.00	0.74-1.36
	All over the chest	11.8 (269)	11.0 (169)	13.6 (100)	0.076	0.79	0.60-1.03
Size of area affected by pain (794)							
	Two inch diameter	10.7 (228)	12.2 (175)	7.7 (53)	0.002**	1.68	1.22-2.32
	Size of patient's palm	58.4 (1240)	59.3 (848)	56.6 (392)	0.253	1.11	0.93-1.34
	Entire chest	30.9 (655)	28.5 (408)	35.7 (247)	0.001**	0.72	0.59-0.87
Palpation tenderness (655)		22.3 (505)	24.3 (371)	18.3 (134)	0.001**	1.44	1.15-1.79
Pain affected by movement (719)		17.0 (373)	18.7 (277)	13.4 (96)	0.002**	1.49	1.16-1.91
Pain affected by breathing (692)		25.8 (573)	26.5 (398)	24.2 (175)	0.258	1.13	0.92-1.38

For peer review only - http://bmjopen.bmj.com/site/about/guidelines.xhtml

Supplemental material 5 (continues) - Prediction of low-risk conditions, univariate analyses

	Total with variable % (n)	Patients with low-risk condition % (n)	Patients without low-risk condition % (n)	Low-risk vs without low-risk, p-value[a]	Odds Ratio	Confidence Interval, 95 %
Troponin T (missing)						
High-sensitive Troponin T, cut-off >14 ng/L (1416)	12 (median)	9 (median)	25 (median)	<0.001***	0.98	0.98-0.98
Troponin T adjusted to Roche Cobas h232 (1416)					0.21	
Tnt <50 ng/L	88.1 (1323)	96.1 (932)	73.6 (391)	<0.001***		
Tnt 51-100 ng/L	6.6 (99)	3.2 (31)	12.8 (68)	<0.001***	0.19	0.12-0.30
Tnt 101-1000 ng/L	4.5 (67)	0.6 (6)	11.5 (61)	0.002**	0.04	0.02-0.10
Tnt >1000 ng/L	0.8 (12)	0.1 (1)	2.1 (11)	–	–	–
ECG (missing[b])						
ECG transmitted to hospital	95.5 (2785)	95.0 (1895)	96.5 (890)	0.064	0.68	0.45-1.02
Sinus Rhythm, SR (235)	83.8 (2248)	88.7 (1622)	73.3 (626)	<0.001***	2.87	2.33-3.54
Sinus Bradycardia (235)	1.3 (34)	1.3 (23)	1.3 (11)	0.949	0.98	0.47-2.01
Sinus Tachycardia (235)	8.9 (240)	7.3 (133)	12.5 (107	<0.001***	0.55	0.42-0.72
Supraventricular Tachycardia, SVT (235)	0.5 (14)	0.1 (1)	1.5 (13)	–	–	–
Atrial Fibrillation/Flutter, AF (235)	14.3 (383)	9.7 (177)	24.1 (206)	<0.001***	0.34	0.27-0.42
Ventricular Tachycardia, VT (235)	0.0 (1)	0.0 (0)	0.1 (1)	–	–	–
Atrial Pacing (235)	1.0 (29)	1.2 (22)	0.8 (7)	0.372	1.48	0.63-3.47
Ventricular Pacing (150)	3.1 (85)	3.0 (57)	3.2 (28)	0.830	0.95	0.60-1.51
AV-block II type 1 (235)	0.0 (1)	0.0 (0)	0.1 (1)	–	–	–
AV-block II type 2 (235)	0.0 (1)	0.1 (1)	0.0 (0)	–	–	–
AV-Block III (235)	0.1 (2)	0 (0.0)	0.2 (2)	–	–	–
ST-Elevation (235)	6.1 (164)	2.2 (41)	14.4 (123)	<0.001***	0.14	0.10-0.20
ST-Depression (235)	7.6 (204)	2.4 (44)	18.7 (160)	<0.001***	0.11	0.08-0.15
T-wave Inversion (235)	14.5 (388)	11.9 (217)	20.0 (171)	<0.001***	0.54	0.43-0.67
Q-wave (235)	4.8 (130)	4.9 (89)	4.8 (41)	0.939	1.02	0.70-1.48
Premature Ventricular Contraction, PVC (235)	5.5 (147)	5.5 (147)	6.6 (56)	0.095	0.75	0.53-1.05
Premature Atrial Contractions, PAC (235)	3.5 (94)	2.9 (53)	4.8 (41)	0.014*	0.59	0.39-0.90
Left Bundle Branch Block, LBBB (235)	6.3 (168)	5.2 (95)	8.5 (73)	0.001**	0.59	0.43-0.81
Right Bundle Branch Block, RBBB (235)	6.3 (170)	5.1 (93)	9.0 (77)	<0.001***	0.54	0.40-0.74
Sinus rhythm and none of abnormalities above (150)	44.6 (1233)	54.0 (1017)	24.5 (216)	<0.001***	3.61	3.02-4.32
QRS-duration (150)	92 (median)	92 (median)	96 (median)	<0.001***	0.99	0.99-0.99
Uninterpretable ECG (132)	0.6 (18)	0.5 (10)	0.9 (8)	0.260	0.59	0.23-1.49

Supplemental material 5 (continues) - Prediction of low-risk conditions, univariate analyses

*p < 0.05
**p < 0.01
***p < 0.001
[a]Logistic regression
[b]When ventricular pacing or uninterpretable remaining ECG interpretation has been treated as missing.

For peer review only

STROBE 2007 (v4) Statement—Checklist of items that should be included in reports of *cohort studies*

Section/Topic	Item #	Recommendation	Reported on page #
**Title and abstract**	1	(*a*) Indicate the study's design with a commonly used term in the title or the abstract	Title
		(*b*) Provide in the abstract an informative and balanced summary of what was done and what was found	Abstract
**Introduction**			
Background/rationale	2	Explain the scientific background and rationale for the investigation being reported	2-3
Objectives	3	State specific objectives, including any prespecified hypotheses	3
**Methods**			
Study design	4	Present key elements of study design early in the paper	3 + title
Setting	5	Describe the setting, locations, and relevant dates, including periods of recruitment, exposure, follow-up, and data collection	3-5
Participants	6	(*a*) Give the eligibility criteria, and the sources and methods of selection of participants. Describe methods of follow-up	3-5
		(*b*) For matched studies, give matching criteria and number of exposed and unexposed	Not applicable
Variables	7	Clearly define all outcomes, exposures, predictors, potential confounders, and effect modifiers. Give diagnostic criteria, if applicable	3-5 + supplement 1 and 2 + table 1
Data sources/ measurement	8*	For each variable of interest, give sources of data and details of methods of assessment (measurement). Describe comparability of assessment methods if there is more than one group	*3-5 + supplement 1 and 2 + table 1*
Bias	9	Describe any efforts to address potential sources of bias	4-5
Study size	10	Explain how the study size was arrived at	4
Quantitative variables	11	Explain how quantitative variables were handled in the analyses. If applicable, describe which groupings were chosen and why	5 + supplement 4+5
Statistical methods	12	(*a*) Describe all statistical methods, including those used to control for confounding	5
		(*b*) Describe any methods used to examine subgroups and interactions	Not applicable
		(*c*) Explain how missing data were addressed	Supplement 4+5
		(*d*) If applicable, explain how loss to follow-up was addressed	3 + Figure 1
		(*e*) Describe any sensitivity analyses	Not applicable
**Results**			

For peer review only

Participants	13*	(a) Report numbers of individuals at each stage of study—eg numbers potentially eligible, examined for eligibility, confirmed eligible, included in the study, completing follow-up, and analysed	5
		(b) Give reasons for non-participation at each stage	Figure 1
		(c) Consider use of a flow diagram	Figure 1
Descriptive data	14*	(a) Give characteristics of study participants (eg demographic, clinical, social) and information on exposures and potential confounders	Supplement 1, table 1
		(b) Indicate number of participants with missing data for each variable of interest	Supplement 4 + 5
		(c) Summarise follow-up time (eg, average and total amount)	Not applicable
Outcome data	15*	Report numbers of outcome events or summary measures over time	Supplement 4 + 5
Main results	16	(*a*) Give unadjusted estimates and, if applicable, confounder-adjusted estimates and their precision (eg, 95% confidence interval). Make clear which confounders were adjusted for and why they were included	Supplement 4 + 5 and Table 2
		(*b*) Report category boundaries when continuous variables were categorized	Supplement 4 + 5
		(*c*) If relevant, consider translating estimates of relative risk into absolute risk for a meaningful time period	Not applicable
Other analyses	17	Report other analyses done—eg analyses of subgroups and interactions, and sensitivity analyses	Not applicable
**Discussion**			
Key results	18	Summarise key results with reference to study objectives	10-11
**Limitations**			
Interpretation	20	Give a cautious overall interpretation of results considering objectives, limitations, multiplicity of analyses, results from similar studies, and other relevant evidence	11-12
Generalisability	21	Discuss the generalisability (external validity) of the study results	11
**Other information**			
Funding	22	Give the source of funding and the role of the funders for the present study and, if applicable, for the original study on which the present article is based	13

*Give information separately for cases and controls in case-control studies and, if applicable, for exposed and unexposed groups in cohort and cross-sectional studies.

Note: An Explanation and Elaboration article discusses each checklist item and gives methodological background and published examples of transparent reporting. The STROBE checklist is best used in conjunction with this article (freely available on the Web sites of PLoS Medicine at http://www.plosmedicine.org/, Annals of Internal Medicine at http://www.annals.org/, and Epidemiology at http://www.epidem.com/). Information on the STROBE Initiative is available at www.strobe-statement.org.

For peer review only

Prehospital stratification in acute chest pain patient into high- and low-risk by emergency medical service – a prospective cohort study

Journal:	*BMJ Open*
Manuscript ID	bmjopen-2020-044938.R2
Article Type:	Original research
Date Submitted by the Author:	01-Apr-2021
Complete List of Authors:	Wibring, Kristoffer; Sahlgrenska Academy, Institute of Health and Care Sciences, Gothenburg University; Halland County, Department of Ambulance and Prehospital Care<br>Lingman, Markus; Sahlgrenska Academy, Department of molecular and clinical Medicine/Cardiology; Halland County, Department of development, Halland Hospital<br>Herlitz, Johan; University of Borås, The Prehospital Research Center Western Sweden<br>Amin, Sinan; Halland County, Department of Cardiology, Halland Hospital<br>Bång , Angela; Sahlgrenska Academy, Institute of Health and Care Sciences, Gothenburg University
**Primary Subject Heading**:	Emergency medicine
Secondary Subject Heading:	Cardiovascular medicine
Keywords:	ACCIDENT & EMERGENCY MEDICINE, Myocardial infarction < CARDIOLOGY, Cardiology < INTERNAL MEDICINE

SCHOLARONE™
Manuscripts

I, the Submitting Author has the right to grant and does grant on behalf of all authors of the Work (as defined in the below author licence), an exclusive licence and/or a non-exclusive licence for contributions from authors who are: i) UK Crown employees; ii) where BMJ has agreed a CC-BY licence shall apply, and/or iii) in accordance with the terms applicable for US Federal Government officers or employees acting as part of their official duties; on a worldwide, perpetual, irrevocable, royalty-free basis to BMJ Publishing Group Ltd ("BMJ") its licensees and where the relevant Journal is co-owned by BMJ to the co-owners of the Journal, to publish the Work in this journal and any other BMJ products and to exploit all rights, as set out in our licence.

The Submitting Author accepts and understands that any supply made under these terms is made by BMJ to the Submitting Author unless you are acting as an employee on behalf of your employer or a postgraduate student of an affiliated institution which is paying any applicable article publishing charge ("APC") for Open Access articles. Where the Submitting Author wishes to make the Work available on an Open Access basis (and intends to pay the relevant APC), the terms of reuse of such Open Access shall be governed by a Creative Commons licence – details of these licences and which Creative Commons licence will apply to this Work are set out in our licence referred to above.

Other than as permitted in any relevant BMJ Author's Self Archiving Policies, I confirm this Work has not been accepted for publication elsewhere, is not being considered for publication elsewhere and does not duplicate material already published. I confirm all authors consent to publication of this Work and authorise the granting of this licence.

For peer review only

Prehospital stratification in acute chest pain patient into high- and low-risk by emergency medical service – a prospective cohort study

Authors
Kristoffer Wibring[1, 2] kristoffer.wibring@gu.se
Markus Lingman[3, 4] markus.lingman@regionhalland.se
Johan Herlitz[5] johan.herlitz@hb.se
Sinan Amin[6] sinan.amin@regionhalland.se
Angela Bång[2] angela.bang@gu.se

1. Department of Ambulance and Prehospital Care, Region Halland, Sweden
2. Institute of Health and Care Sciences, Sahlgrenska Academy, University of Gothenburg, Göteborg, Sweden
3. Department of Molecular and Clinical Medicine/Cardiology, Institute of Medicine, Sahlgrenska Academy, University of Gothenburg, Göteborg, Sweden
4. Department of development, Halland Hospital, Sweden
5. The Prehospital Research Center Western Sweden, University of Borås, Borås, Sweden.
6. Department of cardiology, Halland Hospital, Varberg, Sweden

All authors takes responsibility for all aspects of the reliability and freedom from bias of the data presented and their discussed interpretation.

Corresponding author
Kristoffer Wibring
Ambulanssjukvården Kungsbacka
Varlabergsvägen 29
434 39 Kungsbacka
E-mail: kristoffer.wibring@gu.se
Phone: 0046701757679

For peer review only - http://bmjopen.bmj.com/site/about/guidelines.xhtml

For peer review only

ABSTRACT

Objectives: To describe contemporary characteristics and diagnoses in prehospital patients with chest pain and to identify factors suitable for the early recognition of high- and low-risk conditions.

Design: Prospective observational cohort study

Setting: Two centre study in a Swedish county EMS organisation.

Participants: Unselected inclusion of 2917 patients with chest pain contacting the EMS due to chest pain during 2018.

Primary outcome measures: Low- or high-risk condition, i.e. occurrence of time-sensitive diagnosis on hospital discharge.

Results: Of included EMS missions, 68 % concerned patients with a low-risk condition without medical need of acute hospital treatment in hindsight. Sixteen percent concerned patients with a high-risk condition in need of rapid transport to hospital care. Numerous variables with significant association with low- or high-risk conditions were found. In total high- and low-risk prediction models shared six predictive variables of which ST-depression on ECG and age were most important. Previously known risk factors such as history of acute coronary syndrome, diabetes and hypertension had no predictive value in the multivariate analyses. Some aspects of the symptoms such as pain intensity, pain in the right arm and paleness did on the other hand appear to be helpful. The AUC for prediction of low-risk candidates was 0.786 and for high-risk candidates 0.796. The addition of Troponin in a subset increased the AUC to >0.8 for both.

Conclusions: A majority of patients with chest pain cared for by the EMS suffer from a low-risk condition and have no prognostic reason for acute hospital care given their diagnosis on hospital discharge. A smaller proportion has a high-risk condition and is in need of prompt specialist care. Building models with good accuracy for prehospital identification of these groups is possible. The use of risk stratification models could make a more personalised care possible with increased patient safety.

For peer review only

STRENGTHS AND LIMITATIONS OF THIS STUDY

- Unselected inclusion of a close to complete county population of patients with chest pain contacting the emergency medical services.
- Low rates of missing considering the prehospital nature of data.
- Well examined cohort including data on demographics, previous medical history, symptoms, vital signs, ECG, biochemical markers and diagnosis on discharge.
- Some variables are entailed with high rates of missing when compared to studies conducted in the hospital setting.
- Study conducted in one county reduces generalisability

Keywords
Chest Pain; Emergency Medical Services; Acute Coronary Syndrome; Signs and Symptoms; Triage; Risk Assessment

For peer review only - http://bmjopen.bmj.com/site/about/guidelines.xhtml

BACKGROUND

This report is the first presenting quantitative results in a larger prospective research project (the BRIAN research programme) on improved prehospital risk assessment of patients with chest pain.

Chest pain is one of the most common chief complaints when contacting the emergency medical services (EMS). About 10-15 percent of all EMS missions concern patients with chest pain.[1 2] The role of the EMS when caring for these patients has changed since the introduction of telemedicine solutions for transmission of electrocardiograms (ECG) and direct transport to percutaneous intervention (PCI) centres for patients with ST-elevation myocardial infarction (STEMI).[3] However, these cases constitute only three percent of all patients with chest pain and one third of all patients with acute myocardial infarction (AMI).[2]

Previous studies[4] report that 15 percent of all EMS patients with chest pain have a life-threatening condition. It has been suggested that a large proportion of the patients transported by the EMS due to chest pain could be cared for outside the hospital.[5] Thus we can conclude that patients seen by the EMS before arrival in hospital due to chest pain form a mixed group with different care needs. Guidelines have focused only to a limited extent on personalising the care of patients with chest pain of other origins than STEMI despite the fact that the proportion of patients with AMI has decreased in recent decades from 30 %[6] of all patients seen by the EMS due to chest pain to 10 %.[2 4]

A more differentiated and personalised care for these chest pain patients has the potential of improving efficiency and outcome. For example, patients with non-ST elevation myocardial infarction (NSTEMI) may benefit from bypassing hospitals without PCI capabilities and bypassing the ED for direct transport to cardiac care units (CCU).[7-10] Furthermore, the undifferentiated care of the large group of patients with chest pain contributes to the problematic crowding of EDs. [11 12] Waiting in the ED increases the risk of an adverse outcome for these patients.[13 14] Consistently transporting patients with low-risk conditions to the ED also entails large costs [15] and occupy limited EMS resources. Referral to other destinations than the ED or non-conveyance for low-risk patients could provide improvements in all these aspects.

Hospital research on risk stratification is extensive but research based on data acquired in the prehospital setting is limited.[16] It is important to base prehospital clinical practice on prehospital data since patients who are seen in the prehospital setting differ from those seen at the ED.[6 17-19] This difference might affect pre-test probabilities hence diminishing the validity of the models that have been created in the ED [20-22] when applied in a new (prehospital) setting.

Previous research shows that prehospital risk assessment of patients with chest pain may be possible.[2 16 23 24] However, research is still sparse and often has methodological shortcomings, mainly regarding patient selection and using AMI as the primary endpoint. [20-22] This results in the neglect of other high-risk conditions, such as pulmonary embolism or aortic dissection, and reduces the possibility to identify low-risk patients suitable for non-conveyance. A risk assessment tool for chest pain patients, to be used in the prehospital setting, has been called for in previous research. [25-27] Such a tool might reduce arbitrariness in patient assessment and excess utilisation of emergency care.

For peer review only - http://bmjopen.bmj.com/site/about/guidelines.xhtml

The present report is mainly descriptive and investigates contemporary patient characteristics, outcome and how outcome can be predicted by information accessible in the EMS context. Future reports within this project will focus on how such information could be used and combined in a refined risk assessment tool to be used in the prehospital setting in order to stratify risks and refer patients with chest pain to an appropriate level of care.

Objectives

- To describe contemporary characteristics and diagnoses among prehospital patients with high/low risk conditions presenting with chest pain.
- To identify factors suitable for the early recognition of:
    - patients with time-sensitive conditions in need of immediate care (high-risk conditions)
    - patients with no medical need of hospital treatment, suitable for non-conveyance to hospital (low-risk conditions)
    - present data that can inform the development of a prediction tool

METHODS

Study population

This prospective cohort study was conducted in the county of Halland, Sweden. In all, 3,121 EMS missions were carried out in 2018 concerning patients, ≥18 years old, with a chief complaint of chest pain. All these missions were eligible for inclusion. After excluding patients declining to participate and patients who were lost to follow-up, 2,917 EMS missions were included in data analyses (Figure 1).

Healthcare system

The county of Halland covers an area of 5,500 km^2 and had 329,000 inhabitants in 2018. These are served by two emergency hospitals, including one with PCI capabilities. The EMS consists of eight ambulance stations with 19 ambulance vehicles. In 2018, a total of 30,672 missions were carried out by the EMS (inter-hospital site transports excluded). The EMS is staffed mainly by nurses. An ECG is routinely collected prehospitally and stored digitally using telecommunications.

Data collection

The unique personal identity number assigned to all inhabitants in Sweden made it possible to track each patient throughout the healthcare chain, from EMS mission to hospital discharge and beyond. A novel questionnaire containing fifteen items concerning patient symptoms (Supplemental file 1) was integrated in the digital journal system used by the EMS. The questionnaire was developed by the research group using the findings from two earlier reports within the research project [16 28] along with results and methodology of numerous other studies concerning patients with chest pain. By using electronic tablets, both the questionnaire and the EMS record were available at the bedside during the entire EMS mission.

The questionnaire (Supplemental file 1) contained items mainly focusing on the patients' pain narratives identifying onset, provocation/palliation, quality, radiation, severity etc. Intensity was measured using the Numerical Rating Scale (NRS) ranging from 0-10.[29] If the patient was not able to use the NRS, a Verbal Rating Scale (VRS) ranging from "no pain" to "unbearable pain" was used instead.[30] The answers from the VRS were then transformed into NRS values as follows: "no pain" = 0, "mild pain" = 2 "moderate pain" = 5, "severe pain =

For peer review only - http://bmjopen.bmj.com/site/about/guidelines.xhtml

7" and "unbearable pain" = 9.[31] The questionnaire also contained items regarding nausea/vomiting, dyspnoea, paleness and clamminess.

From the EMS record, data regarding first measured vital signs was collected. From the hospital and primary care medical records, data on diagnosis on discharge from hospital and previous medical history were retrieved.

During the EMS mission a blood sample was obtained and brought to the ED. This blood sample was analysed in hospital for high-sensitive troponin T using Roche Cobas e 601, detecting values ≥ 5 ng/l, cut-off was set to >14 ng/L. Retrieved high-sensitive troponin T-values were also converted into the following intervals <50, 51-100, 101-1000 and >1000 ng/l. In this way, we could stipulate data on troponin T as it would have been presented if Roche's device for bedside troponin T analysis, Cobas h 232, had been used by the EMS.

All registered ECGs were interpreted by either KW or SA using a pre-set ECG interpretation template (Supplemental file 2). Both were blinded to all patient data except age and sex. In cases of uncertainty, interpretation was discussed with senior research cardiologist ML and JH until consensus was reached. Two hundred ECGs were interpreted separately by both KW and SA concerning identification of ST-elevation, ST-depression, right or left bundle branch block. The Kappa coefficient for agreement was 0.810, implying almost perfect agreement.[32] The specific ECG abnormalities included in the Kappa coefficient calculation were chosen based on being most clinically relevant for identification of myocardial ischaemia according to the European Society of Cardiology (ESC).[33 34]

All data except ECG and diagnosis on discharge from hospital was extracted automatically using digital software. Diagnosis on discharge from hospital, according to ICD 10, was collected manually from the medical record by KW. Automatically retrieved data was checked extensively against the original data sources without finding any discrepancies.

Sample size
The planned sample size was 1,500 EMS missions. For a factor present at 10 % of all observations, this would entail that a relative difference of 64 % and an absolute difference of 9 % would be statistically detected. For a factor present at 20 % of all observations, this would entail that a relative difference of 49 % and an absolute difference of 7 % would be detected. For these calculations, 80 % power, significance level of 5 % (two-sided) and a 15 % incidence rate of high-risk conditions are applied. Sample size was later increased to offset the higher rates of missing than predicted in respect of specific variables that were seen early in the inclusion process.

Endpoint
The primary endpoint was a risk-classification group, in terms of low- or high-risk condition. All patients included were classified as having either a low-, intermediate- or high-risk condition as the cause of their chest pain. The adjudication was based on diagnosis on discharge from hospital according to the physician in charge. A high-risk condition was defined as a time-sensitive condition with an increased risk of death and in need of immediate care from a medical point of view in hindsight, for which transport to hospital with highest priority was called upon. An intermediate-risk condition was defined as a diagnosis probably in need of hospital care, but for which time was not judged as a critical factor. A low-risk condition was defined as a diagnosis with no medical need of hospital treatment, suitable for non-conveyance to hospital. This group also included patients with chest pain who remained

For peer review only - http://bmjopen.bmj.com/site/about/guidelines.xhtml

at home and did not visit the ED within 72 hours and who did not die within 30 days. The risk-classification of each diagnosis was carried out independently by KW, JH, ML and AB using a pre-set definition for each risk group blinded to patient characteristics. Thereafter, differences that had arisen were discussed until consensus was reached. Risk-classification was also carried out by an external cardiologist. When comparing this external risk-classification with that of the article authors, a Kappa coefficient of 0,631, substantial agreement, was reached.[32]

Statistical analysis

All variables were analysed using univariate logistic regression. Each variable was analysed twice, once to test association with high-risk conditions and once to test association with low-risk conditions. If a variable was present in fewer than six patients in any of the groups compared, no analysis was carried out. P-values below 0.05 were considered statistically significant. Variables with a statistically significant association were then included in a forward stepwise logistic regression to retain the final independent predictive variables for high- respectively low-risk conditions. These multivariate analyses were executed on a data set of 714 complete cases. Patients with ST-elevation on ECG were excluded given that there are already well-established fast-tracks for these patients. Tnt were excluded from the multivariate analyses due to high rates of missing. Additional multivariate analyses where Tnt was included were also conducted. However, the study was not powered for these specific analyses and the results should therefore be interpreted with caution. All analyses were carried out using IBM SPSS Statistics 26.

Ethical considerations

In this study, all patients eligible for inclusion were subject to an opt-out procedure. The study was approved by the Regional Ethical Review Board in Lund (Dnr 2017/212).

Patient and public involvement

Patients have not been directly involved in planning or conducting this study. Design of the questionnaire (Supplemental file 1) was partly based on patient narratives from a previous study within this research project[28] and other studies based on patient interviews. Furthermore, KW had personal contact with several patients contacting him by phone or e-mail due to the opt-out procedure, both patients wanting to opt-out and those who wanted to remain within the study. Results of the study will presented directly to those patients who requested this when contacting KW.

RESULTS

In total, 139 patients representing 170 EMS missions declined to participate. These patients represent six percent of eligible patients or five percent of eligible EMS missions. In total 2,917 EMS missions concerning 2,352 unique patients with non-traumatic chest pain were included in the data analysis. These constitute 10 % of all patient-related EMS missions during this time period (inter-hospital transports excluded). Priority 1 (highest priority) was given by the emergency medical dispatcher to 63 % of all missions included, and these constitute 11 % of all priority 1-missions within the region. The EMS nurse then triaged patients on scene using the Rapid Triage and Treatment System (RETTS) [35,36] to the highest priority in ten percent of cases and to the lowest priority in four percent. EMS missions were equally distributed regarding sex. Median age was 72 years (Q25-Q75, 58-82) (Supplemental file 3).

For peer review only - http://bmjopen.bmj.com/site/about/guidelines.xhtml

In total, 16 % were classified with a high-risk condition, 16 % had an intermediate-risk condition and 68 % had a low-risk condition (Table 1). The all-cause mortality rate within 30 days was 2.9 % (each unique patient counted only once). In the high-risk group mortality rate within 30 days was 8.9 % and in the low-risk group 0.5 %.

Table 1 - Distribution of risk classification and diagnoses by sex and age

		**Sex**		**Age group**		
	**All % (n)**	**Men % (n)**	**Women % (n)**	**≤50 % (n)**	**51-64 % (n)**	**≥65 % (n)**
	100 (2917)	50.2 (1465)	49.8 (1452)	16.3 (476)	18.5 (539)	65.2 (1902)
High-risk conditions	16.0 (467)	20.3 (298)	11.6 (169)	6.1 (29)	14.8 (80)	18.8 (358)
NSTEMI	6.7 (194)	8.2 (120)	5.1 (74)	2.1 (10)	5.0 (27)	8.3 (157)
STEMI	4.3 (127)	5.9 (87)	2.8 (40)	1.3 (6)	6.5 (35)	4.5 (86)
Unstable angina pectoris	2.1 (60)	2.8 (41)	1.3 (19)	0.4 (2)	1.5 (8)	2.6 (50)
Pulmonary embolism	0.8 (24)	1.0 (14)	0.7 (10)	0.8 (4)	0.7 (4)	0.8 (16)
Undefined AMI*	0.4 (11)	0.7 (10)	0.1 (1)	0.0 (0)	0.2 (1)	0.5 (10)
MINCA/MINOCA	0.2 (7)	0.1 (1)	0.4 (6)	0.0 (0)	0.9 (5)	0.1 (2)
Aortic dissection/aneurysm	0.2 (7)	0.4 (6)	0.1 (1)	0.0 (0)	0.0 (0)	0.4 (7)
Severe arrhythmias and conducting disorders**	0.2 (7)	0.3 (5)	0.1 (2)	0.2 (1)	0.0 (0)	0.3 (6)
Takotsubo	0.2 (5)	0.1 (1)	0.3 (4)	0.4 (2)	0.0 (0)	0.2 (3)
Sepsis	0.1 (4)	0.2 (3)	0.1 (1)	0.2 (1)	0.0 (0)	0.2 (3)
Stroke/TIA	0.1 (4)	0.1 (2)	0.1 (2)	0.4 (2)	0.0 (0)	0.1 (2)
Gastric ulcer with perforation/bleeding	0.1 (4)	0.1 (1)	0.2 (3)	0.0 (0)	0.0 (0)	0.2 (4)
Pulmonary oedema	0.1 (2)	0.0 (0)	0.1 (2)	0.0 (0)	0.0 (0)	0.1 (2)
Other, high risk	0.4 (11)	0.5 (7)	4 0.3	0.2 (1)	0.0 (0)	0.5 (10)
Intermediate-risk conditions	15.6 (455)	16.7 (245)	14.5 (210)	8.6 (41)	10.8 (58)	18.7 (356)

For peer review only - http://bmjopen.bmj.com/site/about/guidelines.xhtml

Atrial fibrillation/flutter	3.8 (112)	3.9 (57)	3.8 (55)	0.6 (3)	3.0 (16)	4.9 (93)
Heart failure (without pulmonary oedema)	2.1 (60)	2.3 (34)	1.8 (26)	0.0 (0)	0.2 (1)	3.1 (59)
Pneumonia	1.8 (52)	1.7 (25)	1.9 (27)	0.2 (1)	0.7 (4)	2.5 (47)
Myocarditis, pericarditis, endocarditis	1.1 (31)	1.6 (23)	0.6 (8)	2.5 (12)	1.3 (7)	0.6 (12)
Syncope and collapse	0.6 (18)	0.5 (7)	0.8 (11)	0.4 (2)	0.7 (4)	0.6 (12)
Aortic valve stenosis	0.6 (17)	0.7 (10)	0.5 (7)	0.0 (0)	0.2 (1)	0.8 (16)
Tumour	0.6 (17)	0.5 (7)	0.7 (10)	0.2 (1)	0.4 (2)	0.7 (14)
Chronic obstructive pulmonary disease (COPD)	0.5 (14)	0.6 (9)	0.3 (5)	0.0 (0)	0.0 (0)	0.7 (14)
Infection, intermediate risk	0.5 (14)	0.5 (7)	0.5 (7)	0.4 (2)	0.6 (3)	0.5 (9)
Supraventricular tachycardia	0.4 (13)	0.3 (5)	0.1 (2)	0.6 (3)	0.7 (4)	0.3 (6)
Cholelithiasis	0.4 (13)	0.5 (7)	0.4 (6)	0.4 (2)	0.4 (2)	0.5 (9)
Pancreatitis	0.3 (9)	0.3 (5)	0.3 (4)	0.6 (3)	0.2 (1)	0.3 (5)
Electrolyte disturbance	0.2 (7)	0.5 (7)	0.0 (0)	0.2 (1)	0.0 (0)	0.3 (6)
Convulsions and seizures	0.2 (6)	0.3 (4)	0.1 (2)	0.6 (3)	0.4 (2)	0.1 (1)
Diverticulitis	0.1 (4)	0.3 (4)	0.0 (0)	0.0 (0)	0.0 (0)	0.2 (4)
Other, intermediate risk	2.3 (67)	2.5 (37)	2.1 (30)	1.7 (8)	2.0 (11)	2.5 (48)
Low-risk conditions	68.4 (1995)	62.9 (922)	73.9 (1073)	85.3 (406)	74.4 (401)	62.5 (1188)
Chest pain, unspecified	41.5 (1211)	39.0 (572)	44.0 (639)	52.7 (251)	50.8 (274)	36.1 (686)
Did not convey (No related ED visit in 72 h or death within 30 days)	9.5 (276)	8.1 (118)	0.3 (158)	16.4 (78)	8.7 (47)	7.9 (151)
Angina pectoris (unstable and spasm induced angina excluded)	3.1 (90)	3.3 (48)	2.9 (42)	0.4 (2)	1.5 (8)	4.2 (80)
Abdominal and pelvic pain	2.1 (62)	1.4 (21)	41 (2.8)	2.7 (13)	1.7 (9)	2.1 (40)
Infection, low risk	1.6 (47)	1.3 (19)	1.9 (28)	1.1 (5)	1.9 (10)	1.7 (32)

For peer review only - http://bmjopen.bmj.com/site/about/guidelines.xhtml

Gastritis/Gastro-oesophageal reflux disease	1.4 (41)	1.2 (18)	1.6 (23)	2.1 (10)	1.3 (7)	1.3 (24)
Dyspnoea and coughing	1.4 (40)	1.5 (22)	1.2 (18)	1.7 (8)	1.1 (6)	1.4 (26)
Palpitation and benign arrhythmias	1.2 (35)	0.8 (11)	1.7 (24)	1.7 (8)	1.1 (6)	1.1 (21)
Anxiety and other mental disorders	0.9 (25)	0.6 (9)	1.1 (16)	2.7 (13)	0.2 (1)	0.6 (11)
Other pain	0.7 (19)	0.6 (9)	0.7 (10)	0.2 (1)	1.3 (7)	0.6 (11)
Anaemia	0.5 (16)	0.5 (8)	0.6 (8)	0.2 (1)	0.7 (4)	0.6 (11)
Vertigo	0.5 (14)	0.5 (7)	0.5 (7)	0.4 (2)	0.2 (1)	0.6 (11)
Back pain	0.3 (10)	0.2 (3)	0.5 (7)	0.2 (1)	0.2 (1)	0.4 (8)
Orthostatic hypotension	0.3 (10)	0.5 (8)	0.1 (2)	0.0 (0)	0.0 (0)	0.5 (10)
Hypertension	0.3 (10)	0.1 (2)	0.6 (8)	0.2 (1)	0.2 (1)	0.4 (8)
Mental and behavioural disorders due to alcohol	0.3 (10)	0.6 (9)	0.1 (1)	0.4 (2)	0.7 (4)	0.2 (4)
Headache	0.2 (6)	0.1 (1)	0.3 (5)	0.6 (3)	0.2 (1)	0.1 (2)
Other, low risk	2.5 (74)	2.6 (38)	2.5 (36)	1.5 (7)	2.6 (14)	2.8 (53)

*Type of myocardial infarction not stated in patient medical record
**Ventricular tachycardia, AV-block III etc.

The cohort was diagnosed with a wide range of medical conditions involving most of the body's organ systems. Most common was musculoskeletal or unspecified chest pain. This diagnosis was given in 41.5 % of all EMS missions. Twelve percent were given an AMI diagnosis on discharge from hospital. In almost ten percent of the EMS missions, the patient was not conveyed to the ED and did not have a related ED visit within 72 hours or died within 30 days (Table 1).

Patient characteristics and patient presentation differ substantially within the cohort. However, there are some dominating aspects. Patients are old and have a substantial comorbidity – most commonly hypertension or mental disorders. They commonly describe central, constant, pressuring pain about the size of a palm starting several hours earlier during rest. Vital signs are most commonly unaffected. In most cases, the ECG presents alterations known to be associated with underlying pathological conditions. These are the most common findings in patients with both low- and high-risk conditions (Supplemental file 4 and 5).

Risk prediction
In total, 26 variables showed significantly increased odds and 16 variables showed significantly decreased odds ratio of having a high-risk condition in the univariate analyses (Supplemental file 4). When predicting low-risk conditions, eleven variables showed significantly increased odds ratio for a low-risk condition whereas 37 variables showed a

For peer review only - http://bmjopen.bmj.com/site/about/guidelines.xhtml

significant increase in the ability to predict the absence of a low-risk condition in the univariate analyses (Supplemental file 5).

When including these variables in a complete cases multivariate analyses 13 and 14 variables remained for high-risk and low-risk prediction respectively. The predictive variables were largely common to both low- and high-risk prediction, with old age and ST-depression as the most important predictors. Previous medical history, except psychiatric disorders, atrial fibrillation/flutter and chronic obstructive pulmonary disease (COPD), had no predictive value in neither high- or low-risk models. In total high- and low-risk prediction models shared six predictive variables, which are written in italics in Table 2. These variables were; age, premature atrial contractions, ST-depression, pain in right arm, paleness and previous medical history of atrial fibrillation/flutter. The area under the receiving operating characteristic curve for prediction of low-risk conditions was 0.786 and 0.796 for high-risk conditions (Figure 2). The additional analyses where Tnt were included resulted in a high-risk model with an AUC of 0.847 and a low-risk model with an AUC of 0.884. Accuracy, in terms of AUC, when adding Tnt thereby increased by 8 % for high-risk prediction and 11 % for low-risk prediction.

Table 2 - Predictors of high- and low-risk conditions after multivariate analysis

	Odds Ratio	CI 95 %	p-value*
**High-risk predictors**			
Age ≤50	-	-	<0.001
Age 51-64	18.98	5.47-65.95	0,000
Age ≥65	7.87	2.12-29.24	0.002
ECG Premature Atrial Contractions, PAC	4.52	1.67-12.25	0.003
ECG ST-depression	3.52	1.63-7.58	0.001
Pain in right arm	2.80	1.48-5.32	0.001
Paleness	2.44	1.44-4.14	<0.001
Male	2.01	1.29-3.14	0.002
Debut during activity	2.01	1.22-3.32	0.006
Pain intensity according to Numeric Rating Scale >8	2.38	1.10-5.13	0.026
ECG T-wave inversion	2.01	1.06-3.82	0.032
Time elapsed since pain onset >3 hours	0.49	0.31-0.77	0.001
Previous atrial fibrillation/flutter	0.42	0.25-0.72	0.001
Previous COPD	0.41	0.18-0.94	0.035
Size of area affected by pain; two inch diameter	0.35	0.13-0.93	0.035
**Low-risk predictors**			
Previous atrial fibrillation/flutter	2.46	1.47-4.11	<0.001
Right sided chest pain	3.88	1.30-11.59	0.015
Previous psychiatric diagnosis (any type)	1.70	1.14-2.54	0.009
Sinus rhythm without abnormalities	1.70	1.11-2.60	0.014
Paleness	0.39	0.25-0.63	<0.001
Breathing rate ≥25 breaths/min	0.45	0.24-0.82	0.009
ECG Atrial Fibrillation/Flutter	0.42	0.23-0.76	0.004

For peer review only - http://bmjopen.bmj.com/site/about/guidelines.xhtml

Age ≤50	-	-	<0.001
Age 51-64	0.40	0.18-0.87	0.021
Age ≥65	0.22	0.11-0.45	<0.001
Pain in right arm	0.28	0.15-0.50	<0.001
Pain in right shoulder	0.33	0.14-0.80	0.014
ECG Premature Atrial Contractions, PAC	0.36	0.14-0.94	0.036
Oxygen saturation ≤91 %	0.32	0.12-0.84	0.020
ECG ST-depression	0.18	0.08-0.42	<0.001
Body temperature >38.0	0.15	0.04-0.63	0.009
*Stepwise forward logistic regression, complete cases, entery p < 0.05, removal p < 0.10			
Italics = Variable common for both high- and low-risk condition prediction models			

For peer review only

DISCUSSION

To our best knowledge, this is the first prospective population wide study of unselected prehospital chest pain patients beyond acute coronary syndromes. This is important since the rule out of acute myocardial infarction alone does not allow EMS to alter conveyance. We present models that can identify high- and low-risk underlying conditions with high accuracy and show that most of these patients suffered from a low-risk condition while sixteen percent had an underlying condition with high risk, such as AMI, pulmonary embolism, aortic dissection, arrhythmias, sepsis and bleeding gastric ulcer. This variety of diagnoses stresses the incentive to have broad inclusion criteria in contrast with previous reports.

Accuracy in terms of AUC and ROC curve shape is quite similar when comparing low- and high-risk prediction models. Both models also seem to improve substantially if adding Tnt.

The present paper aims to inform future risk assessment tools with capabilities to rule in high-risk conditions and rule out low-risk separately. To that end and opposite to expectations classic symptoms of AMI like radiation to left arm or jaws, constant pain, clamminess and dyspnoea did not differ between high- and low-risk conditions in the multivariate analyses. The same was true for previously known risk factors such as history of hypertension, ACS and diabetes. [34] These findings are surprising. It may be explained by our study using a wider range of diagnoses as endpoint compared with previous studies focusing on AMI. [5 37] This is strengthened by ad-hoc analyses showing increased significance for above-mentioned factors when using AMI as endpoint instead of high-risk condition. It may also be explained by prehospital patients having a higher comorbidity compared to patients at the ED [6] and that previous medical history therefore is less useful to discriminate high- from low-risk conditions in the prehospital setting. However, most of these factors differ in the univariate analyses but not in the multivariate ones. Thereby, these factors may still have a predictive value, but when combined with others predictive factors they become less important. Future risk models may need to cover other variables, with ability to contribute to accuracy, than traditional risk factors for AMI. The use of artificial intelligence may be one way to identify such variables.

In both the low- and high-risk models, premature atrial complex (PAC) on ECG have a predictive value. This finding was not expected. Once again, this may be explained by not using AMI as endpoint. This is strengthened by ad-hoc analyses using AMI as endpoint where PAC did not turn out significantly. Given the multiple analyses and the quite wide confidence intervals, this may also be a chance finding.

Vital signs seem to have little predictive value when identifying high-risk conditions. This finding is somewhat surprising, however not unique. For example Frisch et al. [38] reported no association between prehospital heart rate/blood pressure and need of "advanced hospital cardiac care". To some extent, this may be explained by strongly deviating vital signs were quite rare within the cohort, i.e. the vast majority of patients with chest present with normal vital signs. However, tachypnoea, low oxygen saturation and fever all reduced the odds for a low-risk condition. Deviating vital signs thereby seem more useful when ruling out patients from the low-risk group rather than to predict high-risk conditions.

One main finding of this study, and maybe also most relevant in clinical care, is that more than two thirds of all EMS missions concerning patients with chest pain were classified as low-risk, i.e. having no medical need for acute transportation to hospital given the diagnosis on discharge. This implies the substantial magnitude of the impact of a predictive model

For peer review only - http://bmjopen.bmj.com/site/about/guidelines.xhtml

identifying them with high accuracy early in the care chain. Diverting only a proportion of these low-risk patients away from the ED to less resource intensive venues would probably reduce healthcare costs, EMS workload and ED crowding. However, one must have in mind that such a predictive model only refers to the strict medical aspects of being in need of acute hospital care. There can be other circumstances that promote transportation to hospital by ambulance. For example intense anxiety, patient being unable to handle the situation, co-morbidities such as dementia. Such a prediction model should be used to guide the EMS personnel but other factors than medical risk-status must always be considered when assessing patient care needs. It is also of importance to include the patient in the decision making process and take the patient perspective into account.

Among chest pain patients, ten percent remained at home, despite EMS personnel having neither mandate nor instruments to provide such recommendations. This finding indicates that even today, EMS personnel or the patients themselves sometimes deem transport to the ED as inappropriate, despite the patients' chest pain. However, little is known about the appropriateness of EMS decision-making when suggesting this type of triage.

Among the sixteen percent of EMS missions with patients with high-risk conditions, ACS constituted the major part. The portion of high-risk conditions is in line with previous findings.[2 4] Importantly STEMI constitute less than one third of all missions concerning high-risk conditions, confirming work by Terkelsen et al.[2] The fairly low incidence of STEMI shows the importance of taking other high-risk diagnoses into account when risk-assessing patients with chest pain. This would for example enable direct transport to CCU, hospitals with PCI laboratory or capabilities for thoracic surgery.[10] However, referring patients to such instances should be done with great caution given the inclusion of several non-cardiac diagnoses in the high-risk group. A refined model focusing on identification of cardiac patients could be a feasible way to improve the possibilities for such direct transportation patient management.

Seeking to discriminate between high- and low-risk conditions, we also looked for information that would *not* support this aim. In this report, we therefore acknowledge that symptoms typically associated with myocardial ischemia, such as central pain, pressuring pain quality, left arm radiation and affected breathing, are common in both groups. Notably most patients with such symptoms were found in the low risk group. This may limit the usefulness of some symptoms previously reported to be of value in risk assessment and triage. However, other aspects of symptoms such as pain intensity, pain in the right arm and the presence of paleness were helpful in the discrimination of high versus low risk.

Risk assessment in the prehospital setting may serve several purposes. Conveyance decision based on the possibility to identify both low-risk patients suitable for non-hospital care/non-conveyance and high-risk patients in need of prompt specialist care are separate issues.
To further inform a future model supporting the conveyance decision a multivariate analysis was reported. The results of the multivariate analyses presented in this study could form the basis for such a decision support tool. The c-statistics indicate that such a tool could reach a level of accuracy appropriate for clinical use. The fact that several classic symptoms and previously known risk factors of AMI turned out to be of little predictive value indicates that previous risk prediction tools developed in the hospital setting such as the HEART-score and RETTS may be less accurate and invalid when used in the prehospital setting. It is also important to acknowledge that a single factor in itself can constitute grounds for prompt transport to hospital, such as strongly deviating vital signs.

For peer review only - http://bmjopen.bmj.com/site/about/guidelines.xhtml

The models presented in this study need to be refined before tested further. Adding Tnt to the analyses seems to improve accuracy substantially and needs to be investigated further. Analyses on imputed data or a new data set with more complete data regarding Tnt is needed. It would also be preferable to develop a combined model predicting both low- and high-risk conditions, given that good c-statistics may be retained. It would also be of interest to examine the accuracy of reduced models with fewer variables. A combined model including fewer variables would ease clinical usage. The result of such a refined model development will be presented in upcoming reports within the research programme. Prediction models developed using artificial intelligence will also be presented. External validation of the final prediction model(s) is planned to be carried out in multi-centre studies if upcoming results indicate that clinical usage would be appropriate. The prediction model(s) could be integrated in a mobile application or other electronic device to improve clinical usability.

Strengths and limitations

A key strength is the complete picture of the study sample as data provides information on demographics, medical history, a wide range of symptoms, vital signs, ECG, biochemical markers and diagnosis on discharge. However, some variables included entail rather high rates of missing information (Supplemental file 4 and 5). When analysing the distribution of missing data, no skewness of clinical relevance could be identified. This is also confirmed by the fact that the results of the univariate analyses to a large extent confirm previous findings concerning which variables were associated with high-risk conditions. This holds true also for variables with the highest rates of missing e.g. pain intensity [39], pain radiation [39], quality of pain [21] and biochemical markers (Tnt).[40]

Data collection in the prehospital setting is known to be challenging. The situation is often perceived as stressful by the patient, the patient may be physically or mentally unable to provide the information requested, personnel resources are limited and protocol compliance among EMS personnel has been reported to be low.[41 42] All these components contribute to the finding that prehospital research often suffers from high rates of missing information. In this study, we observed that missing rates were highest regarding data where the questionnaire (Supplemental file 1) required the user to write the answer rather than tick a box. The rates of missing data in these cases can thereby probably mainly be explained by limited protocol compliance among EMS personnel.

The high rate of missing data regarding Tnt is a major limitation, since this complicates the inclusion of Tnt in the multivariate analyses. Upcoming reports, using data that are more complete on Tnt, will present more comprehensive prediction models. There are two main reasons for the high rates of missing data on Tnt. Firstly, no blood sample analysis was done if the patient remained at home. Secondly, a blood sample could not be analysed if the patient was transported directly to a hospital outside the county. However, these reasons for not obtaining data on Tnt are not a real-life problem if using a device for prehospital bedside troponin T analysis.

Considering that this was a study based on prehospital data, the rates of missing information must be regarded as low and therefore data may be looked upon as comparatively comprehensive. The varying rates of missing information show possible difficulties using certain variables in a prehospital context. For this reason, it is also important to be careful when selecting variables to be included in a planned decision support tool. A tool requiring

For peer review only - http://bmjopen.bmj.com/site/about/guidelines.xhtml

data on variables which are difficult to collect in the prehospital setting will reduce personnel compliance and thereby be of less clinical value.

This study use risk-classification group as end-point. The classification was based on extensive discussions among experienced colleagues trying to reach a consensus. Risk-classification was also carried out by an external cardiologist, with substantial Cohen's kappa agreement. However, there are not absolute distinctions between risk-classification groups. For example, aortic stenosis and acute pancreatitis can present in different ways. Patients with these diagnoses can be in need of prompt hospital care but in other cases, time is of little importance. Abnormal vital signs may be helpful to discern such cases. Furthermore, there may be other reasons (for example frailty) or intensive pain, which may force the EMS personnel to transport a patient with a low-risk condition to hospital. The objective of the predictions models is to support the EMS personnel in their decision making, not to replace clinical judgement.

Generalisability

The study is strengthened by the nearly complete and unselected inclusion of all EMS missions within the county concerning patients with chest pain during 2018. This increases the generalisability of the results. However, the use of data from only one EMS organisation negatively impacts the external validity. We have no reason to believe that our result is not applicable in other counties in Sweden. The current county includes both urban and rural areas, with a wide range of socio-economic conditions and ethnical diversity. However, we don't have access to specific data on these factors for included patients which is a limitation given that those factors are reported to affect the prehospital care of patients with chest pain. [43] Generalisation of the results beyond Sweden should be done with care, at least outside the western world, given differences in EMS organisations, care financing and EMS contact behaviour.

The level of competence among EMS personnel and equipment available differ between different EMS organisations and countries. This should be considered when discussing future clinical implications. With a minimum of training, included variables are easy to obtain, at least if using external ECG interpretation via telemedicine solutions. Future prediction models should therefore be possible to use by EMS personnel with different competence.

Some of the included variables are subjective, either from a patient or personnel perspective. For example, what is regarded as pain in right shoulder may differ among patients. Different EMS personnel may assess paleness differently, etc. The use of such variables should therefore be limited. Instead, more objective parameters such as, ECG, previous medical history, age and sex should be promoted in future models to improve reliability.

CONCLUSIONS

A majority of patients with chest pain cared for by the EMS suffer from a low-risk condition and have no prognostic reason for acute hospital care given their diagnosis on hospital discharge. A smaller proportion has a high-risk condition and is in need of prompt specialist care. Building models with good accuracy for prehospital identification of these groups is possible. Models optimising rule out will differ from models optimising rule in. However, ECG findings and age are cornerstones in both. Importantly previously known risk factors such as history of acute coronary syndrome, diabetes or hypertension did not have a predictive value in such models. On the other hand previous history of psychiatric disorders and atrial fibrillation/flutter are of importance when risk stratifying prehospital patients with chest pain.

For peer review only - http://bmjopen.bmj.com/site/about/guidelines.xhtml

The use of risk stratification models will make a more personalised care possible with increased patient safety. More research on the value of adding Tnt and how to refine such risk stratification models is needed before clinical testing.

List of abbreviations

ACS - Acute coronary syndrome
AMI - Acute myocardial infarction
CABG - Coronary artery bypass graft
CCU - Cardiac care units
COPD – Chronic obstructive pulmonary disease
ECG - Electrocardiogram
ED - Emergency department
EMS - Emergency medical services
ESC - European Society of Cardiology
NSTEMI - Non-ST elevation myocardial infarction
PAC - Premature atrial complex
PCI - Percutaneous intervention
RETTS - Rapid Emergency Triage and Treatment System
STEMI - ST-elevation myocardial infarction
TNT - Troponin T

Contributors

KW, ML, JH, SA and AB designed the study and planned the data collection. KW, ML, JH and SA interpreted the ECG's. Data analysis was carried out by KW. KW, ML, JH and AB contributed in writing the manuscript. All authors read and approved the final manuscript.

Funding

This study has been funded by the Department of Ambulance and Prehospital Care, Region Halland and the Scientific Council of Region Halland (HALLAND-209901). The funding bodies had no role in the design, conduct, interpretation or writing of the report on this research.

Competing interests

The authors declare that they have no competing interests.

Patient consent for publication

Not required.

Ethics approval

The study was approved by the Regional Ethical Review Board in Lund (Dnr 2017/212). All patients were given the opportunity to withdraw their participation using an opt-out procedure.

Data availability statement

The datasets generated and analysed during the current study are not publicly available due the integrity of patient privacy but are available from the corresponding author on reasonable request and if approved by the Regional Ethical Review Board in Lund.

For peer review only - http://bmjopen.bmj.com/site/about/guidelines.xhtml

Acknowledgements

We want to thank the following persons:

- The EMS personnel of Region Halland for their help with data collection.
- Thomas Thorsson, Vinh Norberg Nguyen and Sasa Pejicic for data extraction.
- Anders Holmén and Ulf Strömberg for statistical support.

Tables, figures and supplemental material

Figure 1 - Flow chart of inclusion process

Figure 2 - Schematic presentation of prediction models accuracy

Table 1 - Distribution of risk classification and diagnoses by sex and age

Table 2 - Prediction models for high- and low-risk conditions

Supplemental material 1 - Analog version of study questionnaire

Supplemental material 2 - ECG interpretation manual

Supplemental material 3 - Description of study sample

Supplemental material 4 - Prediction of high-risk conditions, univariate analyses

Supplemental material 5 - Prediction of low-risk conditions, univariate analyses

For peer review only

REFERENCES

1. Clawson J, Olola C, Heward A, et al. The Medical Priority Dispatch System's ability to predict cardiac arrest outcomes and high acuity pre-hospital alerts in chest pain patients presenting to 9-9-9. *Resuscitation* 2008;78(3):298-306. doi: 10.1016/j.resuscitation.2008.03.229
2. Pedersen CK, Stengaard C, Friesgaard K, et al. Chest pain in the ambulance; prevalence, causes and outcome - a retrospective cohort study. *Scand J Trauma Resusc Emerg Med* 2019;27(1):84. doi: 10.1186/s13049-019-0659-6 [published Online First: 2019/08/29]
3. Hagiwara MA, Bremer A, Claesson A, et al. The impact of direct admission to a catheterisation lab/CCU in patients with ST-elevation myocardial infarction on the delay to reperfusion and early risk of death: results of a systematic review including meta-analysis. *Scandinavian Journal of Trauma Resuscitation & Emergency Medicine* 2014;22 doi: 10.1186/s13049-014-0067-x
4. Rawshani A, Larsson A, Gelang C, et al. Characteristics and outcome among patients who dial for the EMS due to chest pain. *International Journal of Cardiology* 2014;176(3):859-65. doi: 10.1016/j.ijcard.2014.08.004
5. Ishak M, Ali D, Fokkert MJ, et al. Fast assessment and management of chest pain patients without ST-elevation in the pre-hospital gateway (FamouS Triage): ruling out a myocardial infarction at home with the modified HEART score. *Eur Heart J Acute Cardiovasc Care* 2017:2048872616687116. doi: 10.1177/2048872616687116 [published Online First: 2017/01/01]
6. Thang ND, Karlson BW, Bergman B, et al. Characteristics of and outcome for patients with chest pain in relation to transport by the emergency medical services in a 20-year perspective. *American Journal of Emergency Medicine* 2012;30(9):1788-95. doi: 10.1016/j.ajem.2012.02.014
7. Ravn-Fischer A, Karlsson T, Johanson P, et al. Prehospital ECG signs of acute coronary occlusion are associated with reduced one-year mortality. *Int J Cardiol* 2013;168(4):3594-8. doi: 10.1016/j.ijcard.2013.05.064 [published Online First: 2013/05/30]
8. Beygui F, Castren M, Brunetti ND, et al. Pre-hospital management of patients with chest pain and/or dyspnoea of cardiac origin. A position paper of the Acute Cardiovascular Care Association (ACCA) of the ESC. *Eur Heart J Acute Cardiovasc Care* 2015 doi: 10.1177/2048872615604119 [published Online First: 2015/08/27]
9. Rasmussen MB, Stengaard C, Sørensen JT, et al. Predictive value of routine point-of-care cardiac troponin T measurement for prehospital diagnosis and risk-stratification in patients with suspected acute myocardial infarction. *Eur Heart J Acute Cardiovasc Care* 2017:2048872617745893. doi: 10.1177/2048872617745893 [published Online First: 2017/12/01]
10. Stengaard C, Sørensen JT, Rasmussen MB, et al. Editor's Choice-Acute versus subacute angiography in patients with non-ST-elevation myocardial infarction - the NONSTEMI trial phase I. *Eur Heart J Acute Cardiovasc Care* 2017;6(6):490-99. doi: 10.1177/2048872616648468 [published Online First: 2016/05/06]
11. Carter EJ, Pouch SM, Larson EL. The relationship between emergency department crowding and patient outcomes: a systematic review. *J Nurs Scholarsh* 2014;46(2):106-15. doi: 10.1111/jnu.12055 [published Online First: 2013/12/19]
12. Patton GG, Thakore S. Reducing inappropriate emergency department attendances--a review of ambulance service attendances at a regional teaching hospital in Scotland. *Emergency medicine journal : EMJ* 2013;30(6):459-61. doi: 10.1136/emermed-2012-201116 [published Online First: 2012/07/16]

For peer review only - http://bmjopen.bmj.com/site/about/guidelines.xhtml

13. Pines JM, Pollack CV, Diercks DB, et al. The Association Between Emergency Department Crowding and Adverse Cardiovascular Outcomes in Patients with Chest Pain. *Academic Emergency Medicine* 2009;16(7):617-25. doi: 10.1111/j.1553-2712.2009.00456.x
14. Kulstad EB, Kelley KM. Overcrowding is associated with delays in percutaneous coronary intervention for acute myocardial infarction. *Int J Emerg Med* 2009;2(3):149-54. doi: 10.1007/s12245-009-0107-x [published Online First: 2009/06/05]
15. van Dongen DN, Ottervanger JP, Tolsma R, et al. In-Hospital Healthcare Utilization, Outcomes, and Costs in Pre-Hospital-Adjudicated Low-Risk Chest-Pain Patients. *Appl Health Econ Health Policy* 2019 doi: 10.1007/s40258-019-00502-6 [published Online First: 2019/08/06]
16. Wibring K, Herlitz J, Christensson L, et al. Prehospital factors associated with an acute life-threatening condition in non-traumatic chest pain patients - A systematic review. *Int J Cardiol* 2016;219:373-9. doi: 10.1016/j.ijcard.2016.06.066
17. Lavery T, Greenslade JH, Parsonage WA, et al. Factors influencing choice of pre-hospital transportation of patients with potential acute coronary syndrome: An observational study. *Emerg Med Australas* 2017;29(2):210-16. doi: 10.1111/1742-6723.12735 [published Online First: 2017/01/25]
18. Boothroyd LJ, Lambert LJ, Segal E, et al. Comparison of outcomes of ambulance users and nonusers in ST elevation myocardial infarction. *Am J Cardiol* 2014;114(9):1289-94. doi: 10.1016/j.amjcard.2014.07.060 [published Online First: 2014/09/10]
19. Thuresson M, Jarlöv MB, Lindahl B, et al. Factors that influence the use of ambulance in acute coronary syndrome. *Am Heart J* 2008;156(1):170-6. doi: 10.1016/j.ahj.2008.01.020 [published Online First: 2008/03/14]
20. Ishak M, Ali D, Fokkert MJ, et al. Fast assessment and management of chest pain without ST-elevation in the pre-hospital gateway: rationale and design. *Eur Heart J Acute Cardiovasc Care* 2015;4(2):129-36. doi: 10.1177/2048872614549738 [published Online First: 2014/09/08]
21. Stopyra JP, Harper WS, Higgins TJ, et al. Prehospital Modified HEART Score Predictive of 30-Day Adverse Cardiac Events. *Prehosp Disaster Med* 2018;33(1):58-62. doi: 10.1017/S1049023X17007154 [published Online First: 2018/01/10]
22. Alghamdi A, Howard L, Reynard C, et al. Enhanced triage for patients with suspected cardiac chest pain: the History and Electrocardiogram-only Manchester Acute Coronary Syndromes (HE-MACS) decision aid. *Eur J Emerg Med* 2018 doi: 10.1097/MEJ.0000000000000575 [published Online First: 2018/10/04]
23. Rawshani N, Rawshani A, Gelang C, et al. Could ten questions asked by the dispatch center predict the outcome for patients with chest discomfort? *Int J Cardiol* 2016;209:223-5. doi: 10.1016/j.ijcard.2016.02.011 [published Online First: 2016/02/22]
24. van Dongen DN, Tolsma RT, Fokkert MJ, et al. Pre-hospital risk assessment in suspected non-ST-elevation acute coronary syndrome: A prospective observational study. *Eur Heart J Acute Cardiovasc Care* 2018:2048872618813846. doi: 10.1177/2048872618813846 [published Online First: 2018/11/23]
25. Thang ND, Sundstrom BW, Karlsson T, et al. ECG signs of acute myocardial ischemia in the prehospital setting of a suspected acute coronary syndrome and its association with outcomes. *American Journal of Emergency Medicine* 2014;32(6):601-05. doi: 10.1016/j.ajem.2014.03.006
26. Saddichha S, Saxena MK. Is every chest pain a cardiac event? : an audit of patients with chest pain presenting to emergency services in India. *Internal and emergency*

For peer review only - http://bmjopen.bmj.com/site/about/guidelines.xhtml

medicine 2009;4(3):235-9. doi: 10.1007/s11739-009-0246-3 [published Online First: 2009/04/16]
27. Nehme Z, Boyle MJ, Brown T. Diagnostic accuracy of prehospital clinical prediction models to identify short-term outcomes in patients with acute coronary syndromes: a systematic review. *J Emerg Med* 2013;44(5):946-54.e6. doi: 10.1016/j.jemermed.2012.07.078 [published Online First: 2013/01/13]
28. Wibring K, Herlitz J, Lingman M, et al. Symptom description in patients with chest pain - a qualitative analysis of emergency medical calls involving high-risk conditions. *J Clin Nurs* 2019 doi: 10.1111/jocn.14867 [published Online First: 2019/04/02]
29. Hawker GA, Mian S, Kendzerska T, et al. Measures of adult pain: Visual Analog Scale for Pain (VAS Pain), Numeric Rating Scale for Pain (NRS Pain), McGill Pain Questionnaire (MPQ), Short-Form McGill Pain Questionnaire (SF-MPQ), Chronic Pain Grade Scale (CPGS), Short Form-36 Bodily Pain Scale (SF-36 BPS), and Measure of Intermittent and Constant Osteoarthritis Pain (ICOAP). *Arthritis Care Res (Hoboken)* 2011;63 Suppl 11:S240-52. doi: 10.1002/acr.20543
30. Pesonen A, Kauppila T, Tarkkila P, et al. Evaluation of easily applicable pain measurement tools for the assessment of pain in demented patients. *Acta anaesthesiologica Scandinavica* 2009;53(5):657-64. doi: 10.1111/j.1399-6576.2009.01942.x
31. Brunelli C, Zecca E, Martini C, et al. Comparison of numerical and verbal rating scales to measure pain exacerbations in patients with chronic cancer pain. *Health Qual Life Outcomes* 2010;8:42. doi: 10.1186/1477-7525-8-42 [published Online First: 2010/04/22]
32. McHugh ML. Interrater reliability: the kappa statistic. *Biochem Med (Zagreb)* 2012;22(3):276-82.
33. Ibanez B, James S, Agewall S, et al. 2017 ESC Guidelines for the management of acute myocardial infarction in patients presenting with ST-segment elevation: The Task Force for the management of acute myocardial infarction in patients presenting with ST-segment elevation of the European Society of Cardiology (ESC). *Eur Heart J* 2018;39(2):119-77. doi: 10.1093/eurheartj/ehx393
34. Collet JP, Thiele H, Barbato E, et al. 2020 ESC Guidelines for the management of acute coronary syndromes in patients presenting without persistent ST-segment elevation. *Eur Heart J* 2020 doi: 10.1093/eurheartj/ehaa575 [published Online First: 2020/08/29]
35. Widgren BR, Jourak M. Medical Emergency Triage and Treatment System (METTS): a new protocol in primary triage and secondary priority decision in emergency medicine. *J Emerg Med* 2011;40(6):623-8. doi: 10.1016/j.jemermed.2008.04.003 [published Online First: 2008/10/18]
36. Magnusson C, Herlitz J, Axelsson C. Patient characteristics, triage utilisation, level of care, and outcomes in an unselected adult patient population seen by the emergency medical services: a prospective observational study. *BMC Emerg Med* 2020;20(1):7. doi: 10.1186/s12873-020-0302-x [published Online First: 2020/01/30]
37. Roffi M, Patrono C, Collet JP, et al. 2015 ESC Guidelines for the management of acute coronary syndromes in patients presenting without persistent ST-segment elevation: Task Force for the Management of Acute Coronary Syndromes in Patients Presenting without Persistent ST-Segment Elevation of the European Society of Cardiology (ESC). *Eur Heart J* 2016;37(3):267-315. doi: 10.1093/eurheartj/ehv320 [published Online First: 2015/08/29]
38. Frisch A, Heidle KJ, Frisch SO, et al. Factors associated with advanced cardiac care in prehospital chest pain patients. *Am J Emerg Med* 2017 doi: 10.1016/j.ajem.2017.12.003 [published Online First: 2017/12/05]

For peer review only - http://bmjopen.bmj.com/site/about/guidelines.xhtml

39. Reuter PG, Pradeau C, Huo Yung Kai S, et al. Predicting acute coronary syndrome in males and females with chest pain who call an emergency medical communication centre. *Scand J Trauma Resusc Emerg Med* 2019;27(1):92. doi: 10.1186/s13049-019-0670-y [published Online First: 2019/10/17]
40. van Dongen DN, Fokkert MJ, Tolsma RT, et al. Value of Prehospital Troponin Assessment in Suspected Non-ST-Elevation Acute Coronary Syndrome. *Am J Cardiol* 2018 doi: 10.1016/j.amjcard.2018.07.037 [published Online First: 2018/08/20]
41. Maurin Söderholm H, Andersson H, Andersson Hagiwara M, et al. Research challenges in prehospital care: the need for a simulation-based prehospital research laboratory. *Adv Simul (Lond)* 2019;4:3. doi: 10.1186/s41077-019-0090-0 [published Online First: 2019/02/13]
42. Dickert NW, Brown J, Cairns CB, et al. Confronting Ethical and Regulatory Challenges of Emergency Care Research With Conscious Patients. *Ann Emerg Med* 2016;67(4):538-45. doi: 10.1016/j.annemergmed.2015.10.026 [published Online First: 2015/12/18]
43. Asghar Z, Phung VH, Siriwardena AN. Ethnicity and pre-hospital care for people with suspected cardiac pain: cross-sectional study. *J Eval Clin Pract* 2016;22(5):721-5. doi: 10.1111/jep.12523 [published Online First: 2016/03/10]

For peer review only

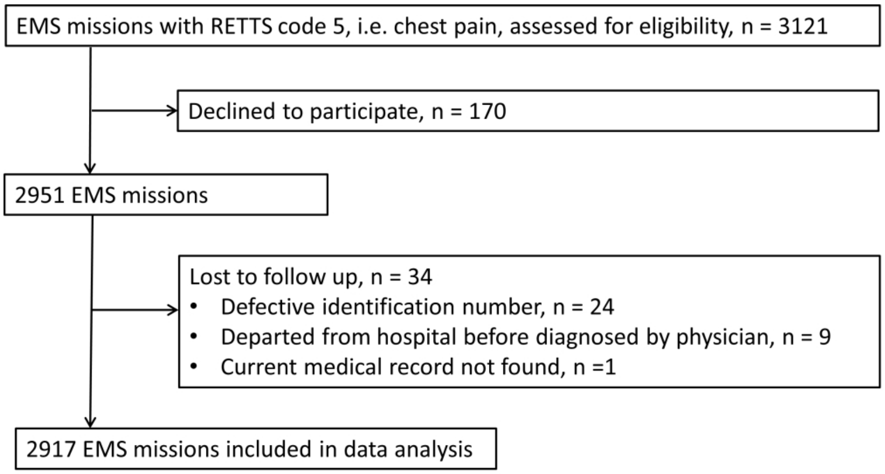

Figure 1 - Flow chart of inclusion process

254x190mm (300 x 300 DPI)

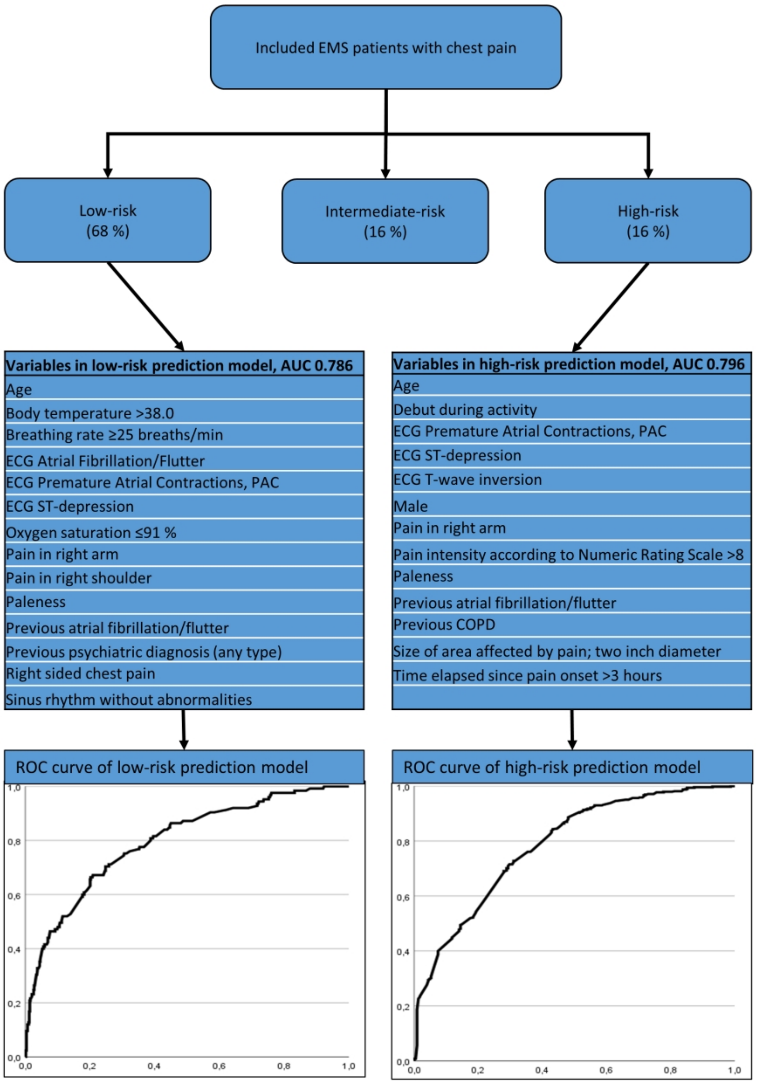

Figure 2 - Schematic presentation of prediction models accuracy

190x275mm (300 x 300 DPI)

Supplemental material 1 - Analog version of study questionnaire

1. Patient is (several options possible)?
□Pale □Clammy □Neither □ Can't tell

2. Patient is nauseas, is vomiting or has been vomiting (several options possible)?
□Nausea □Vomiting/has been vomiting □Neither □ Can't tell

3. The patient experiences his/her breathing as affected?
□Affected □Unaffected □ Can't tell

4. Pain intensity (when resting) on EMS arrival according to NRS (0-10, 0 = "no pain", 10 = "worst imaginable pain").
NRS: 0 1 2 3 4 5 6 7 8 9 10

If NRS is not possible to use, ask the patient to rate his/her pain as follows:
□No pain □Mild pain □Moderate pain □Severe pain □Unbearable pain

5. At what time and date did the pain, inducing the EMS contact, start?
Time: □ Can't tell
Date:

6. How did the pain start?
□Suddenly (seconds) □Quickly (minutes) □Slowly (hours) □ Can't tell

7. Did the pain start in conjunction with (several options possible)?
□Activity □While resting □In pain when waking up □ Can't tell

8. Development of pain (several options possible).
□Pain is constant □Pain come and go □Pain increases gradually □ Can't tell

9. How does the patient describe his/her pain (several options possible)?
Do the patient describe his/her pain as pressuring, cramping, burning, cutting, stabbing, dull, discomfort, swaying, bursting etc. Please write below.

10. Chest pain localisation (several options possible)?
□Central □Left side of chest □Right side of chest □Upper half of chest
□Lower half of chest □Entire chest □ Can't tell

11. Do the patient have pain somewhere else other than in their chest (several different areas/body parts can be specified).
Please write below which area/body part that hurts or if the chest pain is radiating somewhere. If no other pain than chest pain, write: "No other pain".

12. Size of area affected by pain?
□Size of a 5 sek coin □Size of a palm □Entire chest □ Can't tell

13. Chest pain affected by palpation?
□Yes □No □ Can't tell

14. Is pain affected by movement?
□Yes □No □ Can't tell

15. Is pain affected by breathing?
□Yes □No □ Can't tell

Supplemental material 2 - ECG interpretation manual

1. Heart rate: Provided by computer.
2. Sinus rhythm: Positive P wave in lead II with a constant morphology, rate between 50 and 100/min, constant PQ interval and normal QRS morphology.(2)
3. Sinus Bradycardia: Positive P-wave in lead II with a constant morphology, rate <50/min, constant PQ interval and normal QRS morphology.(2)
4. Sinus Tachycardia: Positive P wave in lead II with a constant morphology, rate >100/min, constant PQ interval and normal QRS morphology.(2)
5. Supraventricular Tachycardia: Fast and regular ventricular rhythm usually between 150-200/min. Episodic presentation with sudden onset. Normal QRS-complexes.(2)
6. Atrial Fibrillation: No evident P-waves instead the ECG reveals low-amplitude baseline oscillations (fibrillary or f waves from the fibrillating atria) and an irregular ventricular rhythm. Normal QRS complex duration during AV conduction.(2, 5)
7. Atrial Flutter: In typical atrial flutter, the ECG reveals identically recurring, regular, sawtooth flutter waves, and evidence of continual electrical activity (lack of an isoelectric interval between flutter waves), often best visualized in leads II, III, aVF, or V1. The atrial rate during typical atrial flutter is usually 250 to 350 beats/min with variations in the degree of ventricular conduction, usually 2:1 conduction. Normal QRS complex duration during AV conduction.(2, 5)
8. Atrial pacing: Pacing spike precedes the p wave. Morphology of p wave dependent of lead placement but may appear normal.
9. Ventricular pacing: Pacing spike precedes the QRS complex.
10. Second Degree AV block – II type 1: Progressive prolongation of the PR interval culminating in a non-conducted P wave. The PR interval is longest immediately before the dropped beat. The PR interval is shortest immediately after the dropped beat.(5)
11. Second Degree AV block – II type 2: Intermittent non-conducted P waves without progressive prolongation of the PR interval. The PR interval in the conducted beats remains constant. The P waves continue at a constant rate. The RR interval surrounding the dropped beat(s) is an exact multiple of the preceding RR interval.(5)
12. Third Degree AV block – III: Normal P waves with regular rhythm. Complete absence of AV conduction – none of the supraventricular impulses are conducted to the ventricles. Perfusing rhythm is maintained by a junctional or ventricular escape rhythm.(5)
13. Suspected STEMI: ST-elevation of the J-point in two contiguous leads with ≥ 2.5 mm in men < 40 years, ≥ 2 mm in men > 40 years, or ≥ 1,5 mm in women in V2-V3 and/or ≥ 1 mm in the other leads.(1)
14. ST-Depression: ST-depression ≥ 0.5 mm in at least two contiguous leads. (3)
15. T-wave inversion: Negative electrical axis of the T wave. Positive T wave is always expected at least in the precordial leads V2-V6 and in AVL where the R wave is at least 5mm, deviation from this rule is pathologic.
16. Q-wave: Pathologic q-wave ≥ 25% of R-wave or duration of ≥0,03 s in two contiguous leads. Q-wave or QS-komplex in V2 or V3 is always pathologic. (2)
17. QRS-duration: Provided by computer.
18. Premature Ventricular Complex-PVC: Characterized by the premature occurrence of a QRS complex that is abnormal in shape and has a duration usually exceeding the dominant QRS complex, generally longer than 120 milliseconds. The T wave is usually large and opposite in direction to the major deflection of the QRS. The QRS complex is not preceded by a premature P wave but can be preceded by a nonconducted sinus P wave occurring at its expected time.(5)
19. PAC-SVES: Premature Atrial Complexes: Premature beat with or without a premature P wave that generally differs from a normal sinus P wave. Normal QRS complex. (2,5)

1. Left Bundle Branch Block – LBBB: QRS duration of ≥ 120 ms. Dominant S wave in V1-V3. Broad M-shaped QRS complexes in lateral leads (V5-V6), ST and T waves usually opposite in direction to QRS. Absence of Q waves in lateral leads (I, V5-V6; small Q waves are still allowed in aVL). Prolonged R wave peak time > 60ms in left precordial leads (V5-6).(5,6)
2. Right Bundle Branch Block – RBBB: QRS duration ≥ 120 msec. rsr′, rsR′, or rSR′, patterns in leads V1 and V2. S waves in leads I and V6 ≥ 40 msec wide. Normal time to peak R wave in leads V5 and V6 but > 50 msec in V1.(5)
3. Ventricular Tachycardia: VT is suggested by the occurrence of a series of three or more consecutive, abnormally shaped QRS complexes longer than 120 milliseconds, with the ST-T vector pointing opposite the major QRS deflection. The R-R interval can be regular or varying. Atrial activity can be independent of ventricular activity (AV dissociation), or the atria can be depolarized retrogradely (ventriculoatrial [VA] association). Depending on the particular type of VT, rates range from 70 to 250 beats/min, usually > 100 beats/min.(2,5)
4. Uninterpretable ECG: Meaningful ECG interpretation not possible due to low ECG quality as judged by interpreter.

References

1. Ibanez B, James S, Agewall S, Antunes MJ, Bucciarelli-Ducci C, Bueno H, et al. 2017 ESC Guidelines for the management of acute myocardial infarction in patients presenting with ST-segment elevationThe Task Force for the management of acute myocardial infarction in patients presenting with ST-segment elevation of the European Society. Eur Heart J. 2018 Jan 7;39(2):119–77.
2. Jern Sverker. Klinisk EKG-Diagnostik. 2010;
3. Roffi M, Patrono C, Collet J-P, Mueller C, Valgimigli M, Andreotti F, et al. 2015 ESC Guidelines for the management of acute coronary syndromes in patients presenting without persistent ST-segment elevationTask Force for the Management of Acute Coronary Syndromes in Patients Presenting without Persistent ST-Segment Elevation of th. Eur Heart J. 2016 Jan 14;37(3):267–315.
4. Rhinehardt J, Brady WJ, Perron AD, Mattu A. Electrocardiographic manifestations of Wellens' syndrome. Am J Emerg Med. 2002 Nov 1;20(7):638–43.
5. Douglas P. Zipes, Peter Libby, Robert O. Bonow, Douglas L. Mann, Gordon F. Tomaselli and EB. Braunwald's Heart Disease: A Textbook of Cardiovascular Medicine 11th edition. 2019;
6. Surawicz B, Childers R, Deal BJ, Gettes LS. AHA/ACCF/HRS Recommendations for the Standardization and Interpretation of the Electrocardiogram. Part III: Intraventricular Conduction Disturbances A Scientific Statement From the American Heart Association Electrocardiography and Arrhythmias Committee, . J Am Coll Cardiol. 2009 Mar 17;53(11):976–81.

Supplemental material 3 - Description of study sample

		Sex		Age group		
	All n (%)	Men n (%)	Women n (%)	≤50 n (%)	51-64 n (%)	≥65 n (%)
	2917 (100)	1465 (50.2)	1452 (49.8)	476 (16.3)	539 (18.5)	1902 (65.2)
Mean age (SD)	69 (17)	67 (18)	70 (17)	39 (9)	58 (4)	79 (8)
Median age (Q1-Q3)	72 (58-82)	70 (56-80)	73 (60-83)	41 (31-47)	58 (55-62)	79 (73-85)
Priority by EMD[a]						
Priority 1	1836 (62.9)	936 (63.9)	900 (62.0)	280 (55.5)	232 (59.9)	1233 (64.8)
Priority 2	1049 (36.0)	513 (35)	536 (36.9)	189 (39.7)	208 (38.6)	652 (34.3)
Priority 3	32 (1.1)	16 (1.1)	16 (1.1)	7 (1.5)	8 (1.5)	17 (0.9)
Priority by EMS-personnel[b]						
Priority 1	311 (10.7)	208 (14.2)	103 (7.1)	37 (7.8)	68 (12.3)	206 (10.8)
Priority 2	1969 (67.5)	981 (67.0)	988 (68)	286 (60.1)	355 (65.9)	1328 (69.8)
Priority 3	325 (11.1)	139 (9.5)	186 (12.8)	68 (14.3)	63 (11.7)	194 (10.2)
Priority according to RETTS[c] by EMS-personnel[b]						
Red	290 (9.9)	187 (12.8)	103 (7.1)	32 (6.7)	59 (10.9)	199 (10.5)
Orange	1628 (55.8)	819 (55.9)	809 (55.7)	239 (50.2)	291 (54.0)	1098 (57.7)
Yellow	887 (30.4)	419 (28.6)	468 (32.2)	170 (35.7)	170 (31.5)	547 (28.8)
Green	112 (3.8)	40 (2.7)	72 (5.0)	35 (7.4)	19 (3.5)	58 (3.0)
Transport from primary care centre	508 (17.4)	286 (19.5)	222 (15.3)	77 (16.2)	146 (27.1)	285 (15.0)
Transport to primary care centre	15 (0.5)	8 (0.5)	7 (0.5)	3 (0.6)	0 (0.0)	12 (0.6)
Transported to hospital	2600 (89.1)	1324 (90.4)	1276 (87.9)	388 (81.5)	488 (90.5)	1724 (90.6)
Admitted to hospital (missing = 1)	1409 (48.3)	812 (55.4)	597 (41.1)	123 (25.8)	235 (43.6)	1051 (55.3)
Did not convey	302 (10.4)	133 (9.1)	169 (11.6)	85 (17.9)	51 (9.5)	166 (8.7)
EMS response time (minutes)						
Median EMS dispatch to scene arrival (Q1-Q3)	10 (6-15)	9 (6-15)	10 (6-16)	10 (7-16)	10 (6-17)	9 (6-15)
Median EMS time at scene (Q1-Q3)	25 (18-31)	24 (18-30)	25 (19-32)	22 (16-27)	22 (15-27)	26 20-33)
Median transportation time (Q1-Q3)	25 (13-34)	25 (14-33)	25 (12-35)	25 (13-35)	24 (13-32)	25 13-34)
Median total EMS time (Q1-Q3)	95 (73-118)	95 (73-118)	95 (73-118)	89 (67-115)	92 (70-113)	97 75-120)

[a]Emergency dispatch centre

[b]Emergency medical services

[c]Rapid Emergency Triage and Treatment System

For peer review only

Supplemental material 4 - Prediction of high-risk conditions, univariate analyses

		Total with variable % (n)	Patients with high-risk condition % (n)	Patients without high-risk condition % (n)	High-risk vs without high-risk, p-value[a]	Odds Ratio	Confidence Interval, 95 %
All		2917 (100)	16.0 (467)	84.0 (2450)			
	Men	50.2 (1465)	63.8 (298)	47.6 (1167)	<0.001***	1.93	1.58-2.38
	Women	49.8 (1452)	36.2 (169)	52.4 (1283)			
Age		72 (median)	75 (median)	71 (median)	<0.001***	1.02	1.01-1.03
Age-group							
	Age ≤50	16.3 (476)	6.2 (29)	18.2 (447)			
	Age 51-64	18.5 (539)	17.1 (80)	18.7 (459)	<0.001***	2.68	1.72-4.19
	Age ≥65	65.2 (1902)	76.7 (358)	63.0 (1544)	<0.001***	3.57	2.41-5.29
Previous history of the following according to medical record							
Acute coronary syndrome (AMI, unstable angina)		29.3 (856)	27.8 (130)	29.6 (726)	0.435	0.91	0.74-1.14
Chronic obstructive pulmonary disease		10.5 (307)	7.5 (35)	11.2 (272)	0.021*	0.64	0.45-0.94
Angina pectoris		20.0 (582)	16.9 (79)	20.5 (503)	0.074	0.78	0.61-1.02
Hypertension		58.1 (1696)	63.2 (295)	57.2 (1401)	0.016*	1.28	1.05-1.58
Heart failure		20.6 (600)	18.0 (84)	21.1 (516)	0.132	0.82	0.64-1.06
Diabetes mellitus (type I and II)		19.8 (578)	23.6 (110)	19.1 (468)	0.027*	1.30	1.03-1.65
Stroke		8.2 (240)	7.9 (37)	8.3 (203)	0.794	0.95	0.66-1.37
Kidney disease		12.0 (350)	12.6 (59)	11.9 (291)	0.645	1.07	0.80-1.45
Atrial fibrillation/flutter		26.3 (767)	15.8 (74)	28.3 (693)	<0.001***	0.47	0.37-0.62
Rheumatism		2.3 (66)	1.5 (7)	2.4 (59)	0.230	0.61	0.28-1.36
Cancer (any type)		17.2 (502)	18.6 (87)	16.9 (415)	0.375	1.12	0.87-1.45
Psychiatric diagnosis (any type)		38.9 (1136)	27.4 (128)	41.1 (1008)	<0.001***	0.54	0.43-0.67

For peer review only

Supplemental material 4 (continues) - Prediction of high-risk conditions, univariate analyses

	Total with variable % (n)	Patients with high-risk condition % (n)	Patients without high-risk condition % (n)	High-risk vs without high-risk, p-value[a]	Odds Ratio	Confidence Interval, 95 %
Vital signs on EMS arrival (missing)						
Breathing rate ≤8 breaths/min (22)	0 (0)	0 (0)	0 (0)	–	–	–
Breathing rate ≥25 breaths/min (22)	10.2 (297)	14.7 (68)	9.4 (229)	0.001**	1.65	1.24-2.22
Oxygen saturation ≤91 % (8)	3.8 (110)	6.4 (30)	3.3 (80)	0.001**	2.02	1.32-3.12
Heart rate ≤40 beats/min (18)	0.2 (7)	0.9 (4)	0.1 (3)	–	–	–
Heart rate ≥131 beats/min (18)	3.1 (91)	2.4 (11)	3.3 (80)	0.295	0.71	0.38-1.35
Systolic blood pressure ≤90 mmHg (22)	1.0 (29)	1.5 (7)	0.9 (22)	0.230	1.69	0.72-3.98
Systolic blood pressure ≥220 mmHg (22)	1.0 (28)	0.4 (2)	1.1 (26)	–	–	–
Decreased level of consciousness or new confusion (68)	0.9 (25)	1.5 (7)	0.7 (18)	0.114	2.03	0.84-4.89
Body temperature ≤35.0 (110)	0.2 (6)	0.5 (2)	0.2 (4)	–	–	–
Body temperature >38.0 (110)	2.3 (67)	2.0 (9)	2.5 (58)	0.599	0.82	0.41-1.68
Symptoms on EMS arrival (missing)						
Pale (565)	16.4 (386)	29.1 (111)	14.0 (275)	<0.001***	2.52	1.96-3.26
Clammy (565)	8.7 (204)	14.4 (55)	7.6 (149)	<0.001***	2.05	1.48-2.86
Nausea (576)	27.1 (635)	26.8 (101)	27.2 (534)	0.873	0.98	0.76-1.26
Vomiting (576)	7.0 (165)	7.4 (28)	7.0 (137)	0.754	1.07	0.70-1.63
Affected breathing according to patient (596)	44.6 (1040)	41.9 (158)	45.1 (882)	0.248	0.87	0.70-1.10
Pain intensity according to Numeric Rating Scale. NRS 0-10 (415)						
0	20.3 (509)	17.4 (71)	20.9 (438)	<0.001***		
1	3.8 (94)	3.2 (13)	3.9 (81)	0.976	0.99	0.52-1.87
2	11.5 (287)	9.8 (40)	11.8 (247)	0.996	0.99	0.66-1.52
3	7.6 (189)	8.6 (35)	7.4 (154)	0.136	1.40	0.90-2.19
4	8.1 (202)	6.8 (28)	8.3 (174)	0.976	0.99	0.62-1.59
5	16.7 (418)	15.4 (63)	17.0 (355)	0.629	1.09	0.76-1.58
6	8.3 (207)	6.8 (28)	8.6 (179)	0.882	0.96	0.60-1.56
7	10.5 (263)	12.2 (50)	10.2 (213)	0.068	1.44	0.97-2.15
8	8.2 (206)	9.8 (40)	7.9 (166)	0.069	1.48	0.97-2.28
9	2.6 (64)	4.9 (20)	2.1 (44)	0.001**	2.80	1.56-5.03
10	2.5 (63)	5.1 (21)	2.4 (42)	<0.001***	3.08	1.73-5.51

For peer review only - http://bmjopen.bmj.com/site/about/guidelines.xhtml

Supplemental material 4 (continues) - Prediction of high-risk conditions, univariate analyses

	Total with variable % (n)	Patients with high-risk condition % (n)	Patients without high-risk condition % (n)	High-risk vs without high-risk, p-value[a]	Odds Ratio	Confidence Interval, 95 %
Time elapsed since pain onset >3 hours (1007)	45.2 (863)	40.3 (128)	46.2 (735)	0.053	0.78	0.62-1.00
Debut						
Debut during activity (752)	22.1 (479)	32.8 (116)	20.0 (363)	<0.001***	1.94	1.51-2.50
Debut while resting (752)	65.5 (1419)	59.6 (211)	66.7 (1208)	0.010*	0.73	0.58-0.93
Debut while sleeping (752)	15.8 (342)	11.3 (40)	16.7 (302)	0.012*	0.63	0.45-0.91
Sudden debut, within seconds (875)	35.7 (729)	33.0 (115)	36.3 (614)	0.239	0.86	0.68-1.10
Quick debut, within minutes (875)	35.2 (718)	42.7 (149)	33.6 (569)	0.001**	1.47	1.16-1.86
Slow debut, within hours (875)	29.1 (595)	24.4 (85)	30.1 (510)	0.031*	0.74	0.57-0.97
Constant pain (732)	55.5 (1212)	61.8 (235)	49.9 (977)	<0.001***	1.66	1.31-2.11
Fluctuating pain (732)	40.4 (883)	30.5 (116)	39.2 (767)	0.001**	0.66	0.52-0.84
Pain aggravating over time (732)	10.8 (237)	8.7 (33)	10.4 (204)	0.279	0.80	0.55-1.19
Pain in other parts of the body (1197)						
Head	2.5 (43)	0.7 (2)	2.9 (41)	–	–	–
Throat	10.3 (177)	8.2 (24)	10.7 (153)	0.202	0.74	0.48-1.17
Jaw	5.3 (92)	7.5 (22)	4.9 (70)	0.071	1.58	0.96-2.58
Neck	2.5 (43)	2.7 (8)	2.5 (35)	0.773	1.12	0.56-2.44
Between scapulars	2.2 (37)	4.1 (12)	1.8 (25)	0.014*	2.40	1.19-4.84
Back	15.2 (261)	15.4 (45)	15.1 (216)	0.902	1.02	0.72-1.45
Left shoulder	8.5 (147)	7.9 (23)	8.7 (124)	0.653	0.89	0.57-1.43
Right shoulder	4.2 (72)	6.2 (18)	3.8 (54)	0.067	1.67	0.97-2.89
Left arm	24.0 (412)	33.6 (98)	22.0 (314)	<0.001***	1.79	1.36-2.36
Right arm	8.4 (145)	16.4 (48)	6.8 (97)	<0.001***	2.69	1.86-3.92
Left hand	1.0 (17)	0.7 (2)	1.1 (15)	–	–	–
Right hand	0.3 (6)	0.3 (1)	0.4 (5)	–	–	–
Stomach	7.0 (121)	4.8 (14)	7.5 (107)	0.103	0.62	0.35-1.10
Left leg	1.7 (29)	0.7 (2)	1.9 (27)	–	–	–
Right leg	1.4 (24)	0.0 (0)	1.7 (24)	–	–	–
No other pain	39.3 (676)	36.3 (106)	39.9 (570)	0.249	0.85	0.66-1.11

Supplemental material 4 (continues) - Prediction of high-risk conditions, univariate analyses

	Total with variable % (n)	Patients with high-risk condition % (n)	Patients without high-risk condition % (n)	High-risk vs without high-risk, p-value[a]	Odds Ratio	Confidence Interval, 95 %
Pain quality (1175)						
Band-shaped	3.3 (58)	5.2 (15)	3.0 (43)	0.051	1.81	1.00-3.32
Burning	4.4 (76)	4.5 (13)	4.3 (63)	0.869	1.05	0.57-1.94
Stabbing	9.7 (169)	4.2 (12)	10.8 (157)	0.001**	0.36	0.20-0.66
Cramping	8.7 (151)	9.8 (28)	8.4 (123)	0.461	1.17	0.76-1.81
Dull pain	13.9 (242)	13.3 (38)	14.0 (204)	0.746	0.94	0.65-1.36
Fells like something is on the chest	0.7 (12)	1.0 (3)	0.6 (9)	–	–	–
Discomfort	10.2 (178)	8.7 (25)	10.5 (153)	0.368	0.81	0.52-1.27
Tingling/Stinging	5.7 (99)	1.4(4)	6.5 (95)	–	–	–
Swaying	1.7 (30)	1.4 (4)	1.8 (26)	–	–	–
Pressuring	57.9 (1008)	63.6 (182)	56.7 (826)	0.031*	1.33	1.03-1.74
Heaviness	1.0 (17)	1.4 (4)	0.9 (13)	–	–	–
Aching	2.4 (41)	3.1 (9)	2.2 (32)	0.336	1.44	0.68-3.06
Chest pain localization (640)						
Central pain	53.4 (1215)	64.5 (240)	51.2 (975)	<0.001***	1.73	1.38-2.18
Left side of chest	35.5 (809)	28.0 (104)	37.0 (705)	0.001**	0.66	0.52-0.84
Right side of chest	5.1 (116)	2.4 (9)	5.6 (107)	0.013*	0.41	0.21-0.83
Upper part of chest	6.7 (152)	7.3 (27)	6.6 (125)	0.623	1.11	0.72-1.72
Lower part of chest	9.0 (204)	5.9 (22)	9.6 (182)	0.026*	0.59	0.38-0.94
All over the chest	11.8 (269)	12.6 (47)	11.7 (222)	0.592	1.09	0.78-1.54
Size of area affected by pain (794)						
Two inch diameter	10.7 (228)	5.1 (18)	11.9 (210)	<0.001***	0.39	0.24-0.65
Size of patient's palm	58.4 (1240)	61.0 (216)	57.9 (1024)	0.275	1.13	0.90-1.44
Entire chest	30.9 (655)	33.9 (120)	30.2 (535)	0.174	1.18	0.93-1.51
Palpation tenderness (655)	22.3 (505)	15.8 (58)	23.6 (447)	0.001**	0.60	0.45-0.82
Pain affected by movement (719)	17.0 (373)	11.5 (41)	18.0 (332)	0.003**	0.59	0.42-0.83
Pain affected by breathing (692)	25.8 (573)	17.3 (63)	27.4 (510)	<0.001***	0.55	0.41-0.74

Supplemental material 4 (continues) - Prediction of high-risk conditions, univariate analyses

	Total with variable % (n)	Patients with high-risk condition % (n)	Patients without high-risk condition % (n)	High-risk vs without high-risk, p-value[a]	Odds Ratio	Confidence Interval, 95 %
Troponin T (missing)						
High-sensitive Troponin T, cut-off >14 ng/L (1416)	12 (median)	33 (median)	11 (median)	<0.001***	1.01	1.01-1.02
Troponin T adjusted to Roche Cobas h232 (1416)						
Tnt <50 ng/L	88.1 (1323)	62.9 (161)	93.3 (1162)	<0.001***		
Tnt 51-100 ng/L	6.6 (99)	15.6 (40)	4.7 (59)	<0.001***	4.89	3.17-7.55
Tnt 101-1000 ng/L	4.5 (67)	17.2 (44)	1.8 (23)	<0.001***	13.8	8.12-23.47
Tnt >1000 ng/L	0.8 (12)	4.3 (11)	0.1 (1)	–	–	–
ECG (missing[b])						
ECG transmitted to hospital	95.5 (2785)	97.2 (454)	95.1 (2331)	0.051	1.78	1.00-3.19
Sinus Rhythm, SR (235)	83.8 (2248)	84.7 (377)	83.6 (1871)	0.572	1.08	0.82-1.44
Sinus Bradycardia (235)	1.3 (34)	1.1 (5)	1.3 (29)	0.766	0.86	0.33-2.25
Sinus Tachycardia (235)	8.9 (240)	12.4 (55)	8.3 (185)	0.006	1.56	1.14-2.15
Supraventricular Tachycardia, SVT (235)	0.5 (14)	0.2 (1)	0.6 (3)	–	–	–
Atrial Fibrillation/Flutter, AF (235)	14.3 (383)	13.9 (62)	14.3 (321)	0.818	0.96	0.72-1.30
Ventricular Tachycardia, VT (235)	0.0 (1)	0.0 (0)	0.2 (1)	–	–	–
Atrial Pacing (235)	1.0 (29)	0.4 (2)	1.2 (27)	–	–	–
Ventricular Pacing (150)	3.1 (85)	1.8 (8)	3.3 (77)	0.083	0.52	0.25-1.09
AV-block II type 1 (235)	0.0 (1)	0.2 (1)	0.0 (0)	–	–	–
AV-block II type 2 (235)	0.0 (1)	0.0 (0)	0.0 (1)	–	–	–
AV-Block III (235)	0.1 (2)	0.4 (2)	0.0 (0)	–	–	–
ST-Elevation (235)	6.1 (164)	22.9 (102)	2.8 (62)	<0.001***	10.4	7.46-14.59
ST-Depression (235)	7.6 (204)	23.8 (106)	4.4 (98)	<0.001***	5.82	5.07-9.19
T-wave Inversion (235)	14.5 (388)	22.7 (101)	12.8 (287)	<0.001***	1.99	1.55-2.57
Q-wave (235)	4.8 (130)	6.5 (29)	4.5 (101)	0.074	1.47	0.96-2.26
Premature Ventricular Contraction, PVC (235)	5.5 (147)	7.9 (35)	5.0 (112)	0.016*	1.62	1.09-2.40
Premature Atrial Contractions, PAC (235)	3.5 (94)	6.1 (27)	3.0 (67)	0.002**	2.09	1.32-3.31
Left Bundle Branch Block, LBBB (235)	6.3 (168)	6.5 (29)	6.2 (139)	0.810	1.05	0.70-1.59
Right Bundle Branch Block, RBBB (235)	6.3 (170)	9.2 (41)	5.8 (129)	0.007**	1.65	1.15-2.39
Sinus rhythm and none of abnormalities above (150)	44.6 (1233)	26.5 (120)	48.1 (1113)	<0.001***	0.38	0.31-0.49
QRS-duration (150)	92 (median)	96 (median)	92 (median)	<0.001***	1.00	1.01-1.01
Uninterpretable ECG (132)	0.6 (18)	0.2 (1)	0.7 (17)	–	–	–

Supplemental material 4 (continues) - Prediction of high-risk conditions, univariate analyses

*p < 0.05

**p < 0.01

***p < 0.001

[a]Logistic regression

[b]When ventricular pacing or uninterpretable remaining ECG interpretation has been treated as missing.

For peer review only

Supplemental material 5 - Prediction of low-risk conditions, univariate analyses

		Total with variable % (n)	Patients with low-risk condition % (n)	Patients without low-risk condition % (n)	Low-risk vs without low-risk, p-value[a]	Odds Ratio	Confidence Interval, 95 %
All		2917 (100)	68.4 (1995)	31.6 (922)			
	Men	50.2 (1465)	46.2 (922)	41.1 (379)	<0.001***	0.60	0.51-0.70
	Women	49.8 (1452)	53.8 (1073)	58.9 (543)			
Age		72 (median)	70 (median)	76 (median)	<0.001***	0.98	0.97-0.98
Age-group							
	Age ≤50	16.3 (476)	20.4 (406)	7.6 (70)			
	Age 51-64	18.5 (539)	20.1 (401)	15.0 (138)	<0.001***	0.50	0.36-0.69
	Age ≥65	65.2 (1902)	59.5 (1188)	77.4 (714)	<0.001***	0.29	0.22-0.38
Previous history of the following according to medical record							
Acute coronary syndrome (AMI, unstable angina)		29.3 (856)	29.0 (578)	30.2 (278)	0.515	0.95	0.80-1.12
Chronic obstructive pulmonary disease		10.5 (307)	9.7 (194)	12.3 (113)	0.039*	0.77	0.60-0.99
Angina pectoris		20.0 (582)	20.5 (409)	18.8 (173)	0.275	1.12	0.92-1.36
Hypertension		58.1 (1696)	55.6 (1110)	63.6 (586)	<0.001***	0.72	0.61-0.84
Heart failure		20.6 (600)	18.6 (371)	24.8 (229)	<0.001***	0.69	0.57-0.83
Diabetes mellitus (type I and II)		19.8 (578)	19.4 (388)	20.6 (190)	0.465	0.93	0.77-1.13
Stroke		8.2 (240)	7.5 (150)	9.8 (90)	0.041*	0.75	0.57-0.99
Kidney disease		12.0 (350)	11.0 (219)	14.2 (131)	0.013*	0.75	0.59-0.94
Atrial fibrillation/flutter		26.3 (767)	25.0 (498)	29.2 (269)	0.016*	0.81	0.68-0.96
Rheumatism		2.3 (66)	2.3 (46)	2.2 (20)	0.818	1.06	0.63-1.81
Cancer (any type)		17.2 (502)	15.2 (303)	21.6 (199)	<0.001***	0.65	0.53-0.79
Psychiatric diagnosis (any type)		38.9 (1136)	42.5 (848)	31.2 (288)	<0.001***	1.63	1.38-1.92

For peer review only

For peer review only - http://bmjopen.bmj.com/site/about/guidelines.xhtml

Supplemental material 5 (continues) - Prediction of low-risk conditions, univariate analyses

	Total with variable % (n)	Patients with low-risk condition % (n)	Patients without low-risk condition % (n)	Low-risk vs without low-risk, p-value[a]	Odds Ratio	Confidence Interval, 95 %
Vital signs on EMS arrival (missing)						
Breathing rate ≤8 breaths/min (22)	0 (0)	0 (0)	0 (0)	–	–	–
Breathing rate ≥25 breaths/min (22)	10.2 (297)	6.2 (122)	19.1 (175)	<0.001***	0.28	0.22-0.36
Oxygen saturation ≤91 % (8)	3.8 (110)	1.2 (23)	9.4 (87)	<0.001***	0.11	0.07-0.18
Heart rate ≤40 beats/min (18)	0.2 (7)	0.1 (1)	0.7 (6)	–	–	–
Heart rate ≥131 beats/min (18)	3.1 (91)	0.9 (18)	7.9 (73)	<0.001***	0.11	0.06-0.18
Systolic blood pressure ≤90 mmHg (22)	1.0 (29)	0.6 (12)	1.9 (17)	0.003**	0.32	0.15-0.67
Systolic blood pressure ≥220 mmHg (22)	1.0 (28)	0.8 (16)	1.3 (12)	0.198	0.61	0.29-1.30
Decreased level of consciousness or new confusion (68)	0.9 (25)	0.5 (9)	1.8 (16)	0.001**	0.26	0.12-0.60
Body temperature ≤35.0 (110)	0.2 (6)	0.2 (3)	0.3 (3)	–	–	–
Body temperature >38.0 (110)	2.3 (67)	1.4 (27)	4.5 (40)	<0.001***	0.30	0.18-0.49
Symptoms on EMS arrival (missing)						
Pale (565)	16.4 (386)	11.5 (182)	26.6 (204)	<0.001***	0.36	0.29-0.45
Clammy (565)	8.7 (204)	6.1 (97)	14.0 (107)	<0.001***	0.40	0.30-0.54
Nausea (576)	27.1 (635)	26.6 (421)	28.2 (214)	0.405	0.92	0.76-1.12
Vomiting (576)	7.0 (165)	5.8 (92)	9.6 (73)	0.001**	0.58	0.42-0.80
Affected breathing according to patient (596)	44.6 (1040)	40.7 (640)	52.6 (400)	<0.001***	0.62	0.52-0.74
Pain intensity according to Numeric Rating Scale. NRS 0-10 (415)						
0	20.3 (509)	21.8 (367)	17.4 (142)	0,004*		
1	3.8 (94)	4.3 (72)	2.7 (22)	0.369	1.27	0.76-2.12
2	11.5 (287)	11.9 (200)	10.7 (87)	0.470	0.89	0.65-1.22
3	7.6 (189)	7.3 (123)	8.1 (66)	0.072	0.72	0.51-1.03
4	8.1 (202)	8.7 (147)	6.7 (55)	0.857	1.03	0.72-1.49
5	16.7 (418)	16.1 (272)	17.9 (146)	0.022*	0.72	0.55-0.95
6	8.3 (207)	8.3 (140)	8.2 (67)	0.233	0.81	0.57-1.15
7	10.5 (263)	10.0 (169)	11.5 (94)	0.025*	0.70	0.51-0.96
8	8.2 (206)	7.5 (126)	9.8 (80)	0.004**	0.61	0.43-0.86
9	2.6 (64)	2.2 (37)	3.3 (27)	0.020*	0.53	0.31-0.90
10	2.5 (63)	2.0 (34)	3.6 (29)	0.004**	0.45	0.27-0.77

For peer review only

Supplemental material 5 (continues) - Prediction of low-risk conditions, univariate analyses

	Total with variable % (n)	Patients with low-risk condition % (n)	Patients without low-risk condition % (n)	Low-risk vs without low-risk, p-value[a]	Odds Ratio	Confidence Interval, 95 %
Time elapsed since pain onset >3 hours (1007)	45.2 (863)	45.9 (592)	43.8 (271)	0.394	1.09	0.90-1.32
Debut						
Debut during activity (752)	22.1 (479)	21.0 (306)	24.4 (173)	0.071	0.82	0.67-1.02
Debut while resting (752)	65.5 (1419)	65.6 (956)	65.4 (463)	0.920	1.01	0.84-1.22
Debut while sleeping (752)	15.8 (342)	16.9 (246)	13.6 (96)	0.047*	1.30	1.00-1.67
Sudden debut, within seconds (875)	35.7 (729)	37.5 (513)	32.1 (216)	0.017*	1.27	1.04-1.54
Quick debut, within minutes (875)	35.2 (718)	33.3 (456)	38.9 (262)	0.012*	0.78	0.65-0.95
Slow debut, within hours (875)	29.1 (595)	29.2 (400)	29.0 (195)	0.909	1.01	0.83-1.24
Constant pain (732)	55.5 (1212)	52.9 (778)	60.9 (434)	<0.001***	0.72	0.60-0.86
Fluctuating pain (732)	40.4 (883)	42.7 (629)	35.6 (254)	0.002**	1.35	1.12-1.62
Pain aggravating over time (732)	10.8 (237)	11.0 (162)	10.5 (75)	0.732	1.05	0.79-1.41
Pain in other parts of the body (1197)						
Head	2.5 (43)	3.0 (35)	1.4 (8)	0.051	2.16	0.10-4.70
Throat	10.3 (177)	10.5 (121)	9.9 (56)	0.743	1.06	0.76-1.48
Jaw	5.3 (92)	5.0 (58)	6.0 (34)	0.375	0.82	0.53-1.27
Neck	2.5 (43)	2.2 (26)	3.0 (17)	0.337	0.74	0.40-1.37
Between scapulars	2.2 (37)	1.9 (22)	2.7 (15)	0.308	0.71	0.36-1.38
Back	15.2 (261)	15.5 (179)	14.6 (82)	0.623	1.07	0.81-1.43
Left shoulder	8.5 (147)	8.6 (99)	8.5 (48)	0.983	1.00	0.70-1.44
Right shoulder	4.2 (72)	3.5 (41)	5.5 (31)	0.580	0.63	0.39-1.02
Left arm	24.0 (412)	22.9 (265)	26.1 (147)	0.144	0.84	0.67-1.06
Right arm	8.4 (145)	6.2 (72)	13.0 (73)	<0.001***	0.45	0.32-0.63
Left hand	1.0 (17)	1.2 (14)	0.5 (3)	–	–	–
Right hand	0.3 (6)	0.3 (4)	0.4 (2)	–	–	–
Stomach	7.0 (121)	6.9 (80)	7.3 (41)	0.779	0.95	0.54-1.40
Left leg	1.7 (29)	2.0 (23)	1.1 (6)	0.170	1.88	0.76-4.65
Right leg	1.4 (24)	1.8 (21)	0.5 (3)	–	–	–
No other pain	39.3 (676)	39.8 (461)	38.2 (215)	0.509	1.07	0.87-1.32

For peer review only - http://bmjopen.bmj.com/site/about/guidelines.xhtml

**Supplemental material 5 (continues) - Prediction of low-risk conditions, univariate analyses**							
		Total with variable % (n)	Patients with low-risk condition % (n)	Patients without low-risk condition % (n)	Low-risk vs without low-risk, p-value[a]	Odds Ratio	Confidence Interval, 95 %
Pain quality (1175)							
	Band-shaped	3.3 (58)	3.0 (35)	4.0 (23)	0.250	0.73	0.43-1.25
	Burning	4.4 (76)	4.8 (56)	3.5 (20)	0.229	1.38	0.82-2.32
	Stabbing	9.7 (169)	10.9 (128)	7.2 (41)	0.015*	1.58	1.09-2.28
	Cramping	8.7 (151)	9.0 (1255)	8.1 (46)	0.547	1.12	0.78-1.61
	Dull pain	13.9 (242)	13.8 (162)	14.1 (80)	0.888	0.98	0.73-1.31
	Fells like something is on the chest	0.7 (12)	0.6 (7)	0.9 (5)	0.507	0.68	0.21-2.14
	Discomfort	10.2 (178)	10.7 (125)	9.3 (53)	0.386	1.16	0.83-1.63
	Tingling/Stinging	5.7 (99)	7.1 (83)	2.8 (16)	<0.001***	2.63	1.53-4.54
	Swaying	1.7 (30)	1.8 (21)	1.6 (9)	0.754	1.13	0.52-2.49
	Pressuring	57.9 (1008)	55.8 (654)	62.2 (354)	0.011*	0.77	0.62-0.94
	Heaviness	1.0 (17)	0.9 (11)	1.1 (6)	0.816	0.89	0.33-2.41
	Aching	2.4 (41)	2.0 (24)	3.0 (17)	0.227	0.69	0.36-1.27
Chest pain localization (640)							
	Central pain	53.4 (1215)	50.4 (776)	59.5 (439)	<0.001***	0.69	0.58-0.83
	Left side of chest	35.5 (809)	39.3 (605)	27.6 (204)	<0.001***	1.70	1.40-2.05
	Right side of chest	5.1 (116)	5.7 (87)	3.9 (29)	0.082	1.47	0.95-2.25
	Upper part of chest	6.7 (152)	6.8 (104)	6.5 (48)	0.821	1.01	0.93-1.10
	Lower part of chest	9.0 (204)	9.0 (138)	8.9 (66)	0.985	1.00	0.74-1.36
	All over the chest	11.8 (269)	11.0 (169)	13.6 (100)	0.076	0.79	0.60-1.03
Size of area affected by pain (794)							
	Two inch diameter	10.7 (228)	12.2 (175)	7.7 (53)	0.002**	1.68	1.22-2.32
	Size of patient's palm	58.4 (1240)	59.3 (848)	56.6 (392)	0.253	1.11	0.93-1.34
	Entire chest	30.9 (655)	28.5 (408)	35.7 (247)	0.001**	0.72	0.59-0.87
Palpation tenderness (655)		22.3 (505)	24.3 (371)	18.3 (134)	0.001**	1.44	1.15-1.79
Pain affected by movement (719)		17.0 (373)	18.7 (277)	13.4 (96)	0.002**	1.49	1.16-1.91
Pain affected by breathing (692)		25.8 (573)	26.5 (398)	24.2 (175)	0.258	1.13	0.92-1.38

For peer review only

Supplemental material 5 (continues) - Prediction of low-risk conditions, univariate analyses

	Total with variable % (n)	Patients with low-risk condition % (n)	Patients without low-risk condition % (n)	Low-risk vs without low-risk, p-value[a]	Odds Ratio	Confidence Interval, 95 %
Troponin T (missing)						
High-sensitive Troponin T, cut-off >14 ng/L (1416)	12 (median)	9 (median)	25 (median)	<0.001***	0.98	0.98-0.98
Troponin T adjusted to Roche Cobas h232 (1416)					0.21	
Tnt <50 ng/L	88.1 (1323)	96.1 (932)	73.6 (391)	<0.001***		
Tnt 51-100 ng/L	6.6 (99)	3.2 (31)	12.8 (68)	<0.001***	0.19	0.12-0.30
Tnt 101-1000 ng/L	4.5 (67)	0.6 (6)	11.5 (61)	0.002**	0.04	0.02-0.10
Tnt >1000 ng/L	0.8 (12)	0.1 (1)	2.1 (11)	–	–	–
ECG (missing[b])						
ECG transmitted to hospital	95.5 (2785)	95.0 (1895)	96.5 (890)	0.064	0.68	0.45-1.02
Sinus Rhythm, SR (235)	83.8 (2248)	88.7 (1622)	73.3 (626)	<0.001***	2.87	2.33-3.54
Sinus Bradycardia (235)	1.3 (34)	1.3 (23)	1.3 (11)	0.949	0.98	0.47-2.01
Sinus Tachycardia (235)	8.9 (240)	7.3 (133)	12.5 (107	<0.001***	0.55	0.42-0.72
Supraventricular Tachycardia, SVT (235)	0.5 (14)	0.1 (1)	1.5 (13)	–	–	–
Atrial Fibrillation/Flutter, AF (235)	14.3 (383)	9.7 (177)	24.1 (206)	<0.001***	0.34	0.27-0.42
Ventricular Tachycardia, VT (235)	0.0 (1)	0.0 (0)	0.1 (1)	–	–	–
Atrial Pacing (235)	1.0 (29)	1.2 (22)	0.8 (7)	0.372	1.48	0.63-3.47
Ventricular Pacing (150)	3.1 (85)	3.0 (57)	3.2 (28)	0.830	0.95	0.60-1.51
AV-block II type 1 (235)	0.0 (1)	0.0 (0)	0.1 (1)	–	–	–
AV-block II type 2 (235)	0.0 (1)	0.1 (1)	0.0 (0)	–	–	–
AV-Block III (235)	0.1 (2)	0 (0.0)	0.2 (2)	–	–	–
ST-Elevation (235)	6.1 (164)	2.2 (41)	14.4 (123)	<0.001***	0.14	0.10-0.20
ST-Depression (235)	7.6 (204)	2.4 (44)	18.7 (160)	<0.001***	0.11	0.08-0.15
T-wave Inversion (235)	14.5 (388)	11.9 (217)	20.0 (171)	<0.001***	0.54	0.43-0.67
Q-wave (235)	4.8 (130)	4.9 (89)	4.8 (41)	0.939	1.02	0.70-1.48
Premature Ventricular Contraction, PVC (235)	5.5 (147)	5.5 (147)	6.6 (56)	0.095	0.75	0.53-1.05
Premature Atrial Contractions, PAC (235)	3.5 (94)	2.9 (53)	4.8 (41)	0.014*	0.59	0.39-0.90
Left Bundle Branch Block, LBBB (235)	6.3 (168)	5.2 (95)	8.5 (73)	0.001**	0.59	0.43-0.81
Right Bundle Branch Block, RBBB (235)	6.3 (170)	5.1 (93)	9.0 (77)	<0.001***	0.54	0.40-0.74
Sinus rhythm and none of abnormalities above (150)	44.6 (1233)	54.0 (1017)	24.5 (216)	<0.001***	3.61	3.02-4.32
QRS-duration (150)	92 (median)	92 (median)	96 (median)	<0.001***	0.99	0.99-0.99
Uninterpretable ECG (132)	0.6 (18)	0.5 (10)	0.9 (8)	0.260	0.59	0.23-1.49

Supplemental material 5 (continues) - Prediction of low-risk conditions, univariate analyses

*p < 0.05
**p < 0.01
***p < 0.001
[a]Logistic regression
[b]When ventricular pacing or uninterpretable remaining ECG interpretation has been treated as missing.

For peer review only

STROBE 2007 (v4) Statement—Checklist of items that should be included in reports of *cohort studies*

Section/Topic	Item #	Recommendation	Reported on page #
**Title and abstract**	1	(*a*) Indicate the study's design with a commonly used term in the title or the abstract	Title
		(*b*) Provide in the abstract an informative and balanced summary of what was done and what was found	Abstract
**Introduction**			
Background/rationale	2	Explain the scientific background and rationale for the investigation being reported	2-3
Objectives	3	State specific objectives, including any prespecified hypotheses	3
**Methods**			
Study design	4	Present key elements of study design early in the paper	3 + title
Setting	5	Describe the setting, locations, and relevant dates, including periods of recruitment, exposure, follow-up, and data collection	3-5
Participants	6	(*a*) Give the eligibility criteria, and the sources and methods of selection of participants. Describe methods of follow-up	3-5
		(*b*) For matched studies, give matching criteria and number of exposed and unexposed	Not applicable
Variables	7	Clearly define all outcomes, exposures, predictors, potential confounders, and effect modifiers. Give diagnostic criteria, if applicable	3-5 + supplement 1 and 2 + table 1
Data sources/ measurement	8*	For each variable of interest, give sources of data and details of methods of assessment (measurement). Describe comparability of assessment methods if there is more than one group	*3-5 + supplement 1 and 2 + table 1*
Bias	9	Describe any efforts to address potential sources of bias	4-5
Study size	10	Explain how the study size was arrived at	4
Quantitative variables	11	Explain how quantitative variables were handled in the analyses. If applicable, describe which groupings were chosen and why	5 + supplement 4+5
Statistical methods	12	(*a*) Describe all statistical methods, including those used to control for confounding	5
		(*b*) Describe any methods used to examine subgroups and interactions	Not applicable
		(*c*) Explain how missing data were addressed	Supplement 4+5
		(*d*) If applicable, explain how loss to follow-up was addressed	3 + Figure 1
		(*e*) Describe any sensitivity analyses	Not applicable
**Results**			

For peer review only

Participants	13*	(a) Report numbers of individuals at each stage of study—eg numbers potentially eligible, examined for eligibility, confirmed eligible, included in the study, completing follow-up, and analysed	5
		(b) Give reasons for non-participation at each stage	Figure 1
		(c) Consider use of a flow diagram	Figure 1
Descriptive data	14*	(a) Give characteristics of study participants (eg demographic, clinical, social) and information on exposures and potential confounders	Supplement 1, table 1
		(b) Indicate number of participants with missing data for each variable of interest	Supplement 4 + 5
		(c) Summarise follow-up time (eg, average and total amount)	Not applicable
Outcome data	15*	Report numbers of outcome events or summary measures over time	Supplement 4 + 5
Main results	16	(*a*) Give unadjusted estimates and, if applicable, confounder-adjusted estimates and their precision (eg, 95% confidence interval). Make clear which confounders were adjusted for and why they were included	Supplement 4 + 5 and Table 2
		(*b*) Report category boundaries when continuous variables were categorized	Supplement 4 + 5
		(*c*) If relevant, consider translating estimates of relative risk into absolute risk for a meaningful time period	Not applicable
Other analyses	17	Report other analyses done—eg analyses of subgroups and interactions, and sensitivity analyses	Not applicable
**Discussion**			
Key results	18	Summarise key results with reference to study objectives	10-11
**Limitations**			
Interpretation	20	Give a cautious overall interpretation of results considering objectives, limitations, multiplicity of analyses, results from similar studies, and other relevant evidence	11-12
Generalisability	21	Discuss the generalisability (external validity) of the study results	11
**Other information**			
Funding	22	Give the source of funding and the role of the funders for the present study and, if applicable, for the original study on which the present article is based	13

For peer review only

*Give information separately for cases and controls in case-control studies and, if applicable, for exposed and unexposed groups in cohort and cross-sectional studies.

Note: An Explanation and Elaboration article discusses each checklist item and gives methodological background and published examples of transparent reporting. The STROBE checklist is best used in conjunction with this article (freely available on the Web sites of PLoS Medicine at http://www.plosmedicine.org/, Annals of Internal Medicine at http://www.annals.org/, and Epidemiology at http://www.epidem.com/). Information on the STROBE Initiative is available at www.strobe-statement.org.
